# Genome-wide transcriptional analyses in *Anopheles* mosquitoes reveal an unexpected association between salivary gland gene expression and insecticide resistance

**DOI:** 10.1186/s12864-018-4605-1

**Published:** 2018-03-27

**Authors:** Alison T. Isaacs, Henry D. Mawejje, Sean Tomlinson, Daniel J. Rigden, Martin J. Donnelly

**Affiliations:** 1 Department of Vector Biology, Liverpool School of Tropical Medicine, Liverpool, UK; 2 Infectious Diseases Research Collaboration, Kampala, Uganda; 3 Institute of Integrative Biology, University of Liverpool, Liverpool, UK; 4 Malaria Programme, Wellcome Trust Sanger Institute, Cambridge, UK

**Keywords:** *Anopheles gambiae*, Whole-genome microarray, Salivary gland proteins, Indoor residual spraying, Bendiocarb, Pyrethroids, Long-lasting insecticidal nets, Malaria control

## Abstract

**Background:**

To combat malaria transmission, the Ugandan government has embarked upon an ambitious programme of indoor residual spraying (IRS) with a carbamate class insecticide, bendiocarb. In preparation for this campaign, we characterized bendiocarb resistance and associated transcriptional variation among *Anopheles gambiae s.s.* mosquitoes from two sites in Uganda.

**Results:**

Gene expression in two mosquito populations displaying some resistance to bendiocarb (95% and 79% *An. gambiae s.l.* WHO tube bioassay mortality in Nagongera and Kihihi, respectively) was investigated using whole-genome microarrays. Significant overexpression of several genes encoding salivary gland proteins, including *D7r2* and *D7r4*, was detected in mosquitoes from Nagongera. In Kihihi, *D7r4*, two detoxification-associated genes (*Cyp6m2* and *Gstd3*) and an epithelial serine protease were among the genes most highly overexpressed in resistant mosquitoes. Following the first round of IRS in Nagongera, bendiocarb-resistant mosquitoes were collected, and real-time quantitative PCR analyses detected significant overexpression of *D7r2* and *D7r4* in resistant mosquitoes. A single nucleotide polymorphism located in a non-coding transcript downstream of the D7 genes was found at a significantly higher frequency in resistant individuals. In silico modelling of the interaction between D7r4 and bendiocarb demonstrated similarity between the insecticide and serotonin, a known ligand of D7 proteins. A meta-analysis of published microarray studies revealed a recurring association between D7 expression and insecticide resistance across *Anopheles* species and locations.

**Conclusions:**

A whole-genome microarray approach identified an association between novel insecticide resistance candidates and bendiocarb resistance in Uganda. In addition, a single nucleotide polymorphism associated with this resistance mechanism was discovered. The use of such impartial screening methods allows for discovery of resistance candidates that have no previously-ascribed function in insecticide binding or detoxification. Characterizing these novel candidates will broaden our understanding of resistance mechanisms and yield new strategies for combatting widespread insecticide resistance among malaria vectors.

**Electronic supplementary material:**

The online version of this article (10.1186/s12864-018-4605-1) contains supplementary material, which is available to authorized users.

## Background

Malaria interventions deployed across sub-Saharan Africa over the past 15 years have had an extraordinary impact upon disease transmission, halving the prevalence of *Plasmodium falciparum* infection in endemic regions [1]. Insecticide-based control approaches, such as long-lasting insecticidal nets (LLINs) and indoor residual spraying (IRS) were by far the largest contributors to this outcome. As the use of these vector control tools has increased, so too has the emergence of insecticide-resistant mosquito populations. Mosquito populations resistant to pyrethroids, the only class of insecticide approved for use in LLINs, are starting to outnumber susceptible populations [2]. Furthermore, resistance to other insecticide classes, such as carbamates and organophosphates, is increasingly reported [3, 4].

Malaria is a critical public health challenge in Uganda, with the estimated number of annual cases ranking fourth highest in the world [5]. The investigation described herein is part of a comprehensive malaria surveillance program conducted since 2011 in three areas of Uganda: Walukuba, Kihihi, and Nagongera [6]. During this time, an LLIN distribution campaign has been successful in increasing bed net ownership. A prospective observational study was performed to measure the impact of this campaign upon malaria transmission [7]. In 2013, the proportion of households in Nagongera with at least one LLIN increased from 71 to 95.5%. In 2014, the proportion of households in Kihihi with at least one LLIN increased from 37.5 to 86.5%. Throughout the study period, no evidence of a shift in vector species composition was observed, with *An. gambiae s.s.* remaining the primary vector in these communities. Insecticide resistance testing in both Kihihi and Nagongera revealed high levels of pyrethroid resistance in this species (24–40% mortality), and lower levels of bendiocarb resistance (70 and 83% mortality, respectively) in 2014. This high level of pyrethroid resistance may partially explain why only modest declines in malaria metrics were observed following the universal LLIN distribution campaign.

To combat high levels of malaria transmission in Nagongera, an IRS campaign using the carbamate class insecticide bendiocarb was initiated in December 2014. We collected mosquito specimens prior to IRS deployment to measure pre-existing bendiocarb resistance (95 and 79% *An. gambiae s.l.* WHO tube bioassay mortality in Nagongera and Kihihi, respectively) and identify associated mechanisms present in *An. gambiae s.s*. We then collected samples in the months following the first round of IRS to detect whether these insecticide resistance expression phenotypes persisted. Bendiocarb-resistant *An. gambiae s.s.* in Nagongera did not display any of the commonly observed resistance mechanisms, such as mutations in the insecticide target site (*Ace1*-119S) or overexpression of detoxification enzymes. Instead, a significant association between bendiocarb resistance and overexpression of two salivary gland genes, *D7r2* and *D7r4*, was observed both before and after IRS deployment. Further research is needed to elucidate the role of D7 proteins in bendiocarb resistance, however in silico models support the hypothesis that these proteins are capable of binding insecticide, suggesting that they may play a role in transport or sequestration.

## Results

### Insecticide resistance detection

**Fig. 1:**
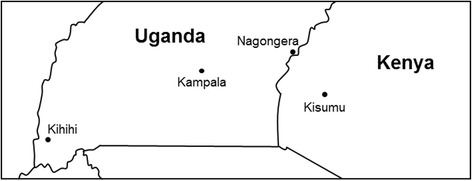
Map of Uganda showing study sites. Larval collections were completed in both Kihihi and Nagongera in January–May 2014. Larval collections were completed in Nagongera in January–May 2015. The geographical origin of the Kisumu laboratory strain of susceptible mosquito is also indicated. The figure was made by the authors using Google Maps (maps.google.com)

WHO tube bioassays were performed between January and May 2014 using *An. gambiae s.l.* mosquitoes collected as larvae in Nagongera and Kihihi (Fig. 1). *An. gambiae s.s.* mortality levels for these DDT, deltamethrin, permethrin, bendiocarb, fenitrothion, and malathion WHO tube bioassays have been previously reported [7]. Species identification PCR revealed differing species compositions in the two sites: 68% of mosquitoes in Nagongera (*n* = 925) were identified as *An. gambiae s.s.* and 32% were identified as *Anopheles arabiensis*, while those from Kihihi were exclusively *An. gambiae s.s* (*n* = 556). All survivors of bendiocarb exposure whose species could be identified were *An. gambiae s.s*.

To investigate the observed bendiocarb resistance phenotype, resistant and unexposed individuals were tested for a mutation in the target of the insecticide, acetylcholinesterase-1. This G119S mutation is widespread in West African *An. gambiae s.s* [8, 9]. A TaqMan assay detected no instance of this mutation in resistant females from either location (*n* = 40), however one heterozygous 119G:119S female from Nagongera was detected among unexposed samples (*n* = 40). Its genotype was confirmed by Sanger sequencing.

### Differential gene expression associated with bendiocarb resistance

Whole-genome microarray analyses were performed to compare gene expression among resistant and unexposed *An. gambiae s.s.* mosquitoes from Nagongera and Kihihi as well as the Kisumu laboratory strain of insecticide-susceptible mosquito originating from western Kenya (Fig. 1). Comparison between sympatric resistant and unexposed mosquitoes controls for geographic origin and life history, while incorporation of the Kisumu strain permits comparison to a fully susceptible strain. Four biological replicates of each treatment were hybridized to whole-genome microarrays using an interwoven loop design. This strategy allowed for identification of genes whose expression was highest in resistant mosquitoes, intermediate in unexposed mosquitoes (of which an estimated 79–95% are susceptible), and lowest in the fully susceptible Kisumu strain.

Several genes met these criteria in addition to displaying greater than 1.2-fold overexpression in resistant vs. unexposed control mosquitoes and an ANOVA F-test *P* value < 0.05 (Table 1). This filtering strategy relied primarily upon directionality, as the ANOVA F-test P value yielded a high number of significant hits. In samples from Kihihi, the transcripts most overexpressed in resistant mosquitoes were those encoding an epithelial serine protease (ESP) (AGAP010240), the cytochrome P450 Cyp6m2, the glutathione S transferase Gstd3, and the salivary gland protein D7r4. In contrast, resistant mosquitoes from Nagongera displayed overexpression of genes encoding a cuticular protein and several salivary gland proteins. Four genes were overexpressed in mosquitoes from both locations: two of unknown function (AGAP009918 and AGAP004528), *apyrase*, and *D7r4*. Resistance-associated overexpression of *D7r2* was also detected in both sites, however it was excluded from the Kihihi candidate gene list by our filtering strategy (2.1 fold change resistant vs. unexposed, ANOVA F-test *P* value = 0.08).

**Table 1:** Genes highly overexpressed in bendiocarb-resistant *An. gambiae s.s.* mosquitoes, as measured by whole-genome microarrays

Location	Transcript ID	Gene name	Fold change resistant vs. unexposed	*p*-value	Fold change resistant vs. Kisumu	*p*-value
Nagongera	AGAP003334-RA	*CPLCX2*	2.4	2.97E-03	2.4	2.92E-03
	AGAP008282-RA	*D7r2*	2.4	4.95E-02	7.2	9.22E-05
	AGAP002198-RA	*glycine N-methyltransferase*	2.1	6.79E-03	2.5	1.34E-03
	AGAP000603-RA	Unknown	1.9	3.32E-02	2.5	3.84E-03
	AGAP008281-RA	*D7r4*	1.9	1.97E-02	2.8	3.98E-04
	AGAP000611-RA	gSG1a	1.7	2.49E-04	1.8	8.17E-05
	AGAP011971-RA	*apyrase*	1.7	2.01E-02	3.1	3.28E-05
	AGAP009623-RA	*glyceraldehyde 3-phosphate dehydrogenase*	1.7	4.63E-03	2.0	8.13E-04
	AGAP008216-RA	*GSG7*	1.7	4.99E-04	1.8	2.34E-04
	AGAP011368-RA	*OBP57*	1.5	4.18E-02	1.9	4.97E-03
Kihihi	AGAP010240-RA	*ESP*	2.5	1.06E-04	4.6	6.77E-08
	AGAP008212-RA	*Cyp6m2*	2.3	1.75E-03	3.7	1.01E-05
	AGAP004382-RA	*Gstd3*	2.2	1.25E-03	3.0	4.82E-05
	AGAP008281-RA	*D7r4*	2.1	6.84E-03	2.8	3.24E-04
	AGAP008358-RA	*Cyp4h17*	2.0	2.01E-03	2.9	1.86E-05
	AGAP001956-RA	*Niemann-Pick Type C-2*	2.0	7.01E-03	5.1	2.93E-07
	AGAP005501-RA	*dehydrogenase/reductase SDR family member 11 precursor*	2.0	1.08E-03	3.4	6.42E-07
	AGAP005498-RB	*phospholipid scramblase 2*	1.9	2.78E-06	3.0	2.87E-10
	AGAP005645-RA	*dehydrogenase/reductase SDR family member 11 precursor*	1.9	7.48E-04	2.8	2.03E-06
	AGAP010066-RA	Unknown	1.9	1.38E-05	2.4	6.83E-08

Microarray results were filtered to select genes whose expression was highest in resistant mosquitoes, intermediate in unexposed mosquitoes (of which an estimated 79–95% are susceptible), and lowest in the fully susceptible strain. Furthermore, genes with less than 1.2-fold overexpression in resistant vs. unexposed control mosquitoes or those with an ANOVA F-test *P* value > 0.05 were excluded. The 10 most highly overexpressed genes (resistant vs. unexposed) from each location are displayed above.

**Fig. 2:**
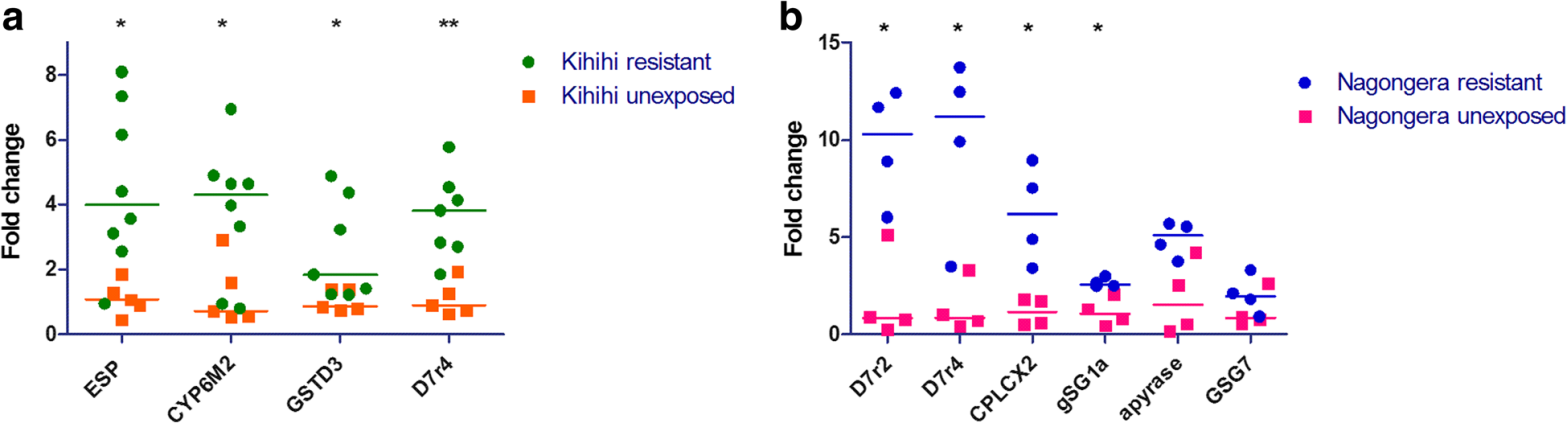
Expression of insecticide resistance candidates in *An. gambiae s.s.* mosquitoes collected in 2014, measured by real-time quantitative PCR. Gene expression in mosquitoes collected from Kihihi (**a**) and Nagongera (**b**). Bendiocarb-resistant mosquitoes were selected using a standard WHO tube bioassay. Unexposed mosquitoes were placed in a tube with a control paper. Each RNA sample was extracted from pools of five mosquitoes. The y-axis depicts the level of transcript in each sample relative to the unexposed control group. The median value for each treatment is indicated by a line. Two-tailed Mann-Whitney *p* < 0.05 (*) or *p* < 0.01 (**)

To validate these findings, real-time quantitative PCR (qPCR) was used to measure expression of nine resistance candidates in the same pooled RNA samples that were assayed by whole-genome microarray (Fig. 2). For each collection site, the five genes mostly highly overexpressed in bendiocarb-resistant mosquitoes were evaluated. When functional primers could not be identified or gene function was unknown, the analyses were expanded to include additional candidates (*gSG1a*, *apyrase*, *GSG7*). Additional resistant mosquitoes from the Kihihi collections were also included: one additional unexposed control pool and either 3 additional resistant pools (GSTD3, D7r4) or 4 additional resistant pools (ESP, CYP6M2). Among the Kihihi candidates, overexpression of all four genes was confirmed (Two-tailed Mann-Whitney, *ESP*, *Cyp6m2, Gstd3 p* < 0.05, *D7r4 p* < 0.01). Resistant mosquitoes from Nagongera displayed overexpression of *D7r2*, *D7r4*, *gSG1a*, and *CPLCX2* relative to unexposed controls (Two-tailed Mann-Whitney *p* < 0.05). Overexpression of *apyrase* and *GSG7* was not validated by qPCR (Two-tailed Mann-Whitney, *p* > 0.05).

### Bendiocarb resistance post-IRS

**Fig. 3:**
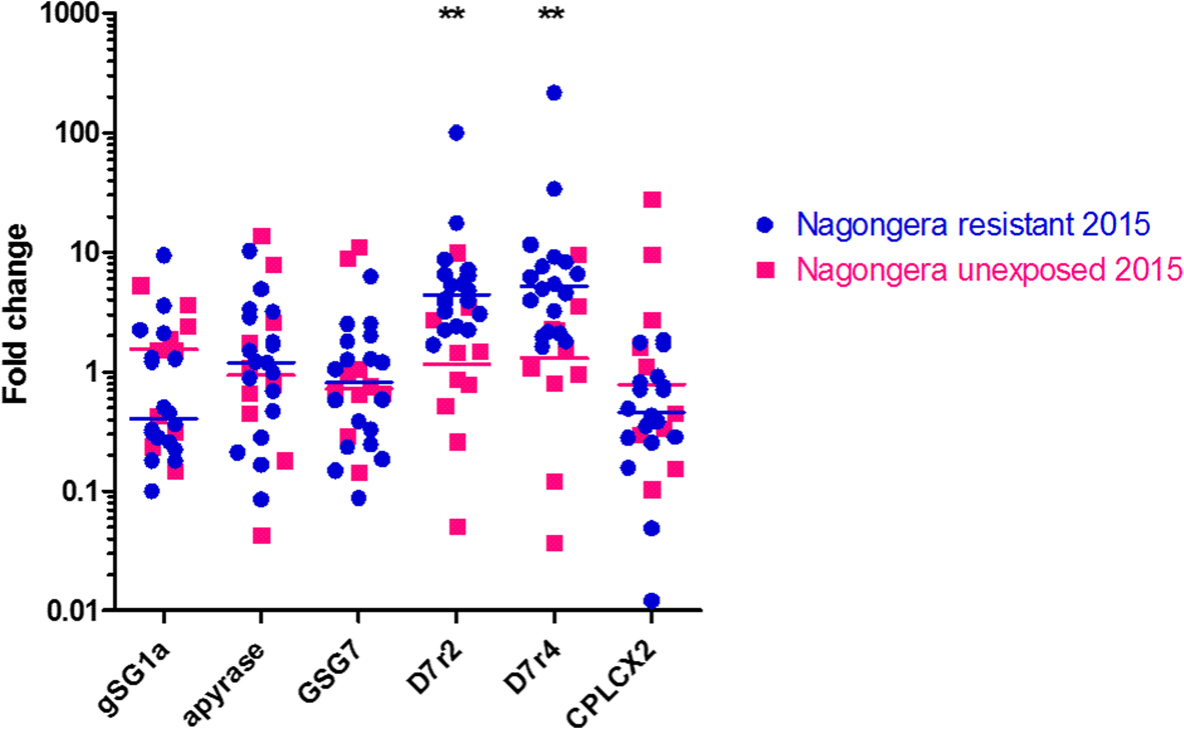
Expression of insecticide resistance candidates in *An. gambiae s.s.* mosquitoes collected in Nagongera in January–May 2015, measured by real-time quantitative PCR. Resistant and unexposed mosquitoes were selected as described in Fig. 2. Each RNA sample was extracted from an individual mosquito. The y-axis depicts the level of transcript in each sample relative to the unexposed control group. The median value for each treatment is indicated by a line. Two-tailed Mann-Whitney *p* < 0.01 (**)

Additional bendiocarb WHO tube bioassays were performed following the bendiocarb IRS campaign implemented between December 2014 and February 2015 in Nagongera. Species identification PCR revealed a decrease in the proportion of *An. gambiae s.s*. mosquitoes, with only 27% identified as *An. gambiae s.s.* (*n* = 89) (Fisher’s exact test, two-sided *p* < 0.0001). Surprisingly, bioassay mortality observed in May 2015 (98%) was significantly higher than that observed in May 2014 (95%) (Fisher’s exact test, two-sided *p* = 0.004, May 2014 *n* = 411, May 2015 *n* = 520). A TaqMan assay detected no instance of the G119S target-site mutation in either resistant or unexposed control mosquitoes (*n* = 27). Real-time quantitative PCR analyses performed on individual mosquitoes detected overexpression of both *D7r2* and *D7r4* in resistant individuals (Two-tailed Mann-Whitney, *D7r2 p* = 0.002, *D7r4 p* = 0.005) (Fig. 3). Comparison of *D7r2* and *D7r4* expression in these individuals revealed a highly significant correlation (Spearman *r* = 0.9075, *p* < 0.0001), which supports the hypothesis that these genes are co-regulated. Real-time quantitative PCR analyses did not detect overexpression of *gSG1a*, *apyrase*, *GSG7*, or *CPLCX2* in resistant mosquitoes (Two-tailed Mann-Whitney, *p* > 0.05),

### Silencing of D7r2 and D7r4

Members of the D7 family are among the most highly-expressed salivary proteins in *An. gambiae*, and aid in blood feeding by scavenging host amines that induce vasoconstriction, platelet aggregation, and pain [10, 11]. In *An. gambiae*, three long (*D7L1–3*) and five short D7 (*D7r1–5*) have been identified, and their expression is limited to female mosquitoes [10, 12]. RNAi-induced knockdown of *D7r2* and *D7r4* expression via intrathoracic injection was performed to test whether these transcripts could be silenced. Kisumu strain mosquitoes were injected with either double-stranded RNA (dsRNA) targeting the *D7r2* transcript (dsD7r2), the *D7r4* transcript (dsD7r4) or control dsRNA containing green fluorescent protein sequence (dsGFP). Attempts to silence these genes yielded highly variable results (32–89% reduction in transcript abundance) as well as off-target silencing of other D7 genes, and thus were not pursued further.

### Identification of resistance- and overexpression-associated single nucleotide polymorphisms

A subset of bendiocarb-resistant and unexposed females were examined to determine whether resistance was associated with non-synonymous mutations in the coding sequence of either D7r2 or D7r4. Six variant amino acids were detected in the D7r4 coding region of four resistant individuals, however no association with resistance was detected (Additional file 1). Within three resistant individuals whose D7r2 coding region was sequenced, no amino acids varying from the *An. gambiae* reference genome were detected (Additional file 1).

To identify nucleic acid variants responsible for the elevated *D7r2* and *D7r4* expression levels observed in bendiocarb-resistant mosquitoes, two nearby genomic regions were examined for the presence of associated single nucleotide polymorphisms (SNPs). First, the *D7r2/D7r4* intergenic regions from resistant and unexposed mosquitoes displaying a wide range of expression levels were amplified and sequenced. This *D7r2*/*D7r4* intergenic region has previously been tested for its promoter activity in transgenic fruit flies, where it reproduced the tissue-specificity of *D7r4* expression [13]. Seven sequences derived from five individuals displaying low levels of *D7r4* expression (fold change 1–2 relative to unexposed controls) were compared to twenty-three sequences derived from twelve individuals displaying high levels of *D7r4* expression (fold change 4–216 relative to unexposed controls). The amplified sequences varied widely in size, ranging from 1.2 to 1.8 kilobase pairs (Additional file 2). Variant base pairs identified by alignment were counted for low and high-expressing individuals. To identify a variant base pair associated with high expression, these results were filtered for variants appearing in at least five high expression individuals, and absent from low expression individuals. This threshold was set based on the hypothesis that A) high expression is a dominant trait, and therefore could be present at a minimum in only half of high expression alleles; B) the variant bases conferring high expression levels may differ among the population, and therefore may be identical in only a fraction of high expression individuals. This filtering strategy did not identify any variants associated with expression of *D7r4*. These low and high expression sequence sets were then analysed for relative enrichment of known transcription factor binding motifs using the Analysis of Motif Enrichment (AME) tool [14]. A search of these sequences for motifs from the JASPAR CORE Insects database did not detect any significant enrichment of motifs in high expression sequences (Wilcoxon rank-sum test, *p* > 0.05).

Next, a region downstream of the short D7 cassette that encodes an apparently non-coding transcript was examined for expression-associated SNPs. It has been previously speculated that this transcript may have a role in regulation of D7 expression [12]. For this analysis, the sample set was expanded to include all *An. gambiae s.s.* (*n* = 28) from the 2015 Nagongera collections whose expression had been measured. Thirteen individuals displaying low levels of *D7r4* expression (fold change 0–2 relative to unexposed controls) were compared to fifteen individuals displaying high levels of *D7r4* expression (fold change 3–216 relative to unexposed controls). Following molecular cloning, one sequence per individual was included in the comparison. A cytosine/thymine SNP at genomic location 3R:8564156 displayed an association with both D7 expression level and bendiocarb resistance (Additional file 3). A real-time quantitative PCR using a locked nucleic acid probe was used to genotype the 28 individuals in the expanded sample set, 14 additional individuals from the 2015 Nagongera collections, and 40 individuals from the 2014 Nagongera collections. A statistically significant association between the 3R:8564156 SNP and bendiocarb resistance was detected, with 15/38 resistant and 7/44 unexposed individuals displaying the SNP (Fisher’s exact test, *p* = 0.03, Odds ratio = 3, *n* = 82). In contrast, this SNP was less prevalent in mosquitoes from the 2014 Kihihi collections, appearing in only 2/24 resistant and 2/25 unexposed individuals (*n* = 49). No mosquito homozygous for the non-reference nucleotide was identified.

### In silico modelling

**Fig. 4:**
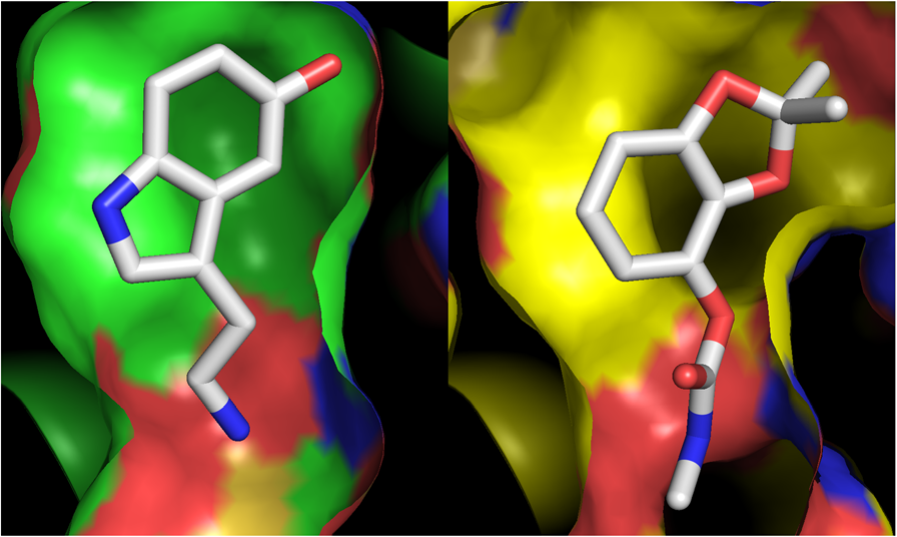
Comparison between serotonin binding to D7r4 protein and the predicted mode of binding of bendiocarb. Serotonin, as visualised in the crystal structure (PDB code 2qeh; [15]), is displayed on the left, and the ROSIE server [16, 17] predicted pose of bendiocarb for the same protein on the right, each in a stick representation. Ligand atoms are coloured white (carbon), red (oxygen) or blue (nitrogen). Solvent-accessible protein surfaces were calculated within PyMOL using the default solvent molecule radius of 1.4 Å. Surface contributed by carbon atoms is coloured green in the serotonin complex and yellow in the bendiocarb complex. In both, red and blue are used for surface contributed by oxygen or nitrogen atoms, respectively. The figure was made with PyMOL (pymol.org)

The crystal structure of D7r4 protein and a modelled structure of D7r2 were used to assess the possibility of direct binding of bendiocarb to these proteins. Having noted a strong chemical structural similarity between bendiocarb and serotonin, as visualised in complex with D7r4 [15], we confirmed that bendiocarb could be manually positioned in the D7r4 pocket in a similar binding conformation as serotonin, where it was well accommodated with minimal steric clashes. In order to strengthen this hypothesis more objectively, computational small molecule docking was done at the ROSIE server [16, 17], which indeed predicted a conformation similar to that derived manually (Fig. 4).

Although D7r2 and D7r4 are relatively distantly related, sharing only 32% sequence identity, they align with only a single one residue insertion in the former vs the latter. This, along with the conservation of most cavity-lining residues, ensures a comparatively reliable prediction of the cavity shape and size in D7r2, although not of the quality desirable for automated docking methods. Interestingly, D7r2 is predicted to have a distinctly larger cavity, the difference arising principally from the replacement of two residues, Phe 110 and Leu 43 in D7r4, with the smaller Val in both cases (Additional file 4).

### D7 family genes are overexpressed in multiple species of insecticide-resistant mosquitoes across Africa

A meta-analysis of published microarray data was performed to evaluate whether D7 expression is associated with insecticide resistance in other species of *Anopheles* mosquitoes. A PubMed and Google Scholar search for publications containing microarray data from insecticide-resistant *Anopheles* mosquitoes yielded 35 results. Fourteen of these publications described genome-wide microarrays performed on whole female mosquitoes selected with insecticides. Eight of the fourteen studies analysed documented a significant association between expression of D7 genes and insecticide resistance. Overexpression of both the short and long form D7 genes has been previously associated with resistance to two insecticide classes: carbamates (bendiocarb), and pyrethroids (etofenprox, permethrin, deltamethrin, lambda-cyhalothrin) in four mosquito species (*Anopheles funestus, Anopheles gambiae s.s, Anopheles coluzzi, Anopheles arabiensis*) (Table 2). All five known short form D7 proteins and two long form D7 proteins (D7L1 and 2) were associated with insecticide resistance. In most instances, overexpression of D7 was observed, however underexpression of *D7r2* and a long form D7 were seen in etofenprox- and lambda-cyhalothrin-resistant *An. funestus* from Zambia [18]. The hypothesis that D7 expression is associated with insecticide resistance is also supported by longitudinal data. A deltamethrin-resistant population of *An. coluzzi* collected from Burkina Faso displayed overexpression of *D7L2* in 2011, and even higher expression in 2012, concomitant with an increase in insecticide resistance [19]. In this same study, a comparison of the highly-resistant VK7 strain and the moderately resistant Tengrela strain identified *D7L2* as the second mostly highly overexpressed gene in the more resistant strain. Furthermore, when permethrin-resistant *An. arabiensis* from Uganda were compared to the Dongola susceptible laboratory strain, *D7r2* was the most highly-overexpressed gene [20].

**Table 2:** Whole-genome microarray studies in which D7 expression was associated with insecticide resistance

Mosquito Species	Country of collection	Insecticide	D7 gene	Reference
*An. funestus*	Malawi	Bendiocarb, Permethrin	*D7r1*, *D7r2*^a^, *D7r3*^b^	[48]
*An. funestus*	Mozambique, Malawi	Permethrin	*D7r4*	[49]
*An. funestus*	Zambia	Deltamethrin	*D7r1*	[18]
*An. funestus*	Zambia	Etofenprox, Lambda-cyhalothrin	*D7r2*, long form D7^c^	[18]
*An. funestus*	Senegal	Lambda-cyhalothrin	*D7r1*	[49]
*An. coluzzi*	Burkina Faso, Cote d’Ivoire	Deltamethrin	*D7L2*	[19]
*An. gambiae s.s.*	Zambia	Deltamethrin	*D7r1, D7r2, D7r3, D7r5, D7L2*	[18]
*An. arabiensis*	Sudan	Permethrin	*D7r2, D7r4*	[50]
*An. arabiensis*	Uganda	Permethrin	*D7r2, D7r4, D7L1*	[20]
*An. arabiensis*	Zanzibar	Lambda-cyhalothrin	*D7r4*	[51]

^a^*D7r2* overexpressed in bendiocarb-resistant vs. unexposed control, overexpressed in pyrethroid-resistant vs. unexposed control, and underexpressed in unexposed control vs. susceptible colony

^b^*D7r3* overexpressed in bendiocarb-resistant vs. unexposed control, underexpressed in unexposed control vs. susceptible colony

^c^*D7r2* and long form D7 underexpressed in resistant vs. susceptible colony

## Discussion

Recent campaigns to control malaria transmission in Uganda through a combination of LLIN distribution and IRS have been highly effective in decreasing malaria metrics, however our sampling of mosquito larval populations indicates that a reservoir of bendiocarb-resistant *An. gambiae s.s.* persists [7]. Measures of human biting rate decreased significantly after initiation of the IRS programme in Nagongera, indicating that mosquito populations were suppressed [7]. Furthermore, surveillance using CDC light traps did not detect a change in the relative abundance of mosquito species. In contrast, our sampling of larval pools revealed a decrease in the frequency of *An. gambiae s.s.* mosquitoes following IRS, suggesting that this species was disproportionately affected, perhaps due to it being more anthrophophilic than *An. arabiensis* [21]*.*

It is likely that several different mutations circulate within this population of *An. gambiae s.s.* and collectively confer bendiocarb resistance, however one resistance-associated phenotype that was observed both before and after IRS deployment is overexpression of genes encoding the D7r2 and D7r4 salivary gland proteins. The overexpression of *apyrase*, *GSG7*, *gSG1a*, and *CPLCX2*, detected in Nagongera in 2014 but not in 2015, may have associated fitness costs that caused these phenotypes to be eliminated from the local mosquito population, or may have been false positives. Our results demonstrate an association between D7 overexpression and bendiocarb resistance in *An. gambiae s.s.* mosquitoes, however further studies are needed to confirm whether D7 overexpression is a causative factor in resistance, or instead closely associated with a resistance-conferring variant.

The hypothesis that D7 family proteins are directly involved in bendiocarb resistance is supported by four key observations: (1) Overexpression of D7r4 in resistant mosquitoes from both Nagongera and Kihihi (2) Overexpression of D7 genes in mosquitoes collected from Nagongera both pre- and post-IRS intervention (3) Association between a D7-adjacent SNP and both D7 overexpression and bendiocarb resistance (4) The ability of the D7r4 protein structure (and probably D7r2 too) to accommodate bendiocarb in the central binding pocket, in a fashion that resembles that observed experimentally for the chemically similar serotonin.

We hypothesize that D7 overexpression is symptomatic of a disruption in the tissue-specificity of D7 expression which allows these proteins to interact with insecticides in tissues other than the salivary glands. While expression of D7 genes has primarily been associated with the salivary glands, lower expression levels have been detected in tissues such as the malpighian tubules [22]. Our sequencing of the D7r2/D7r4 intergenic region, which has previously been shown to direct salivary gland-specific expression [13], revealed high sequence diversity. In future, we will measure D7 expression in the dissected tissues of bendiocarb-resistant mosquitoes to establish whether mutations that confer overexpression also alter tissue-specificity.

Importantly, the identification of a D7 overexpression-associated SNP provides evidence that the high level of D7 expression observed in resistant mosquitoes is not simply a reaction to insecticide exposure, but is associated with an identifiable genotype. Further studies will be needed to determine whether the apparently non-coding transcript downstream of the short D7 cassette regulates expression of these genes. Our ability to test for an association between this SNP and bendiocarb resistance was limited by the fact that unexposed controls contain a mixture of resistant and susceptible individuals. However, the increase in SNP frequency among resistant mosquitoes following implementation of bendiocarb IRS supports our hypothesis that this SNP is associated with both D7 overexpression and bendiocarb resistance. The 3R:8564156 SNP was found in approximately half of the D7-overexpressing individuals tested, indicating that other SNPs conferring this phenotype remain to be discovered. Additional putative candidate resistance polymorphisms within this locus are being investigated using the *Anopheles gambiae* 1000 genomes dataset available for this region [23]. Preliminary analyses have detected no selective sweeps in the region of the D7 genes, consistent with the hypothesis that multiple SNPs could confer D7-mediated insecticide resistance. Such resistance-associated SNPs could enable surveillance of mosquito populations and testing of archival DNA samples to measure the spread of this resistance mechanism.

Despite repeated appearances among the top microarray hits for genes overexpressed in resistant mosquitoes, the D7 protein family has never been functionally validated as an insecticide resistance candidate. The crystal structure of D7r4 revealed that the short form D7 proteins are structurally related to arthropod odorant-binding proteins, and exhibit a single binding site [15]. In a recent study, a D7 protein from the *Aedes aegypti* mosquito was observed to bind dengue virions and envelope protein, suggesting that this family of proteins may have a broader range of associated phenotypes than previously thought [24]. We hypothesize that if D7 proteins at least partially confer insecticide resistance, they are more likely to do so by binding and sequestering insecticide or insecticide metabolites rather than by any direct detoxification. This hypothesis is strongly supported by the structural compatibility of the D7r4 protein’s central binding pocket and bendiocarb. The D7r2 protein is likely to have a larger and differently shaped pocket, potentially suggesting that it has an overlapping but distinct specificity for bound ligands. Preliminary docking experiments suggest that the larger pocket found in D7r2 may permit binding of pyrethroids such as permethrin, however there is insufficient structural information for the D7r2 protein to make strong conclusions. Such promiscuous binding of insecticides has precedent in cytochrome P450 enzymes such as CYP6M2, which metabolizes type I and II pyrethroids as well at DDT [25, 26]. Furthermore, the ability to bind structurally diverse molecules has been demonstrated in proteins such as the bacterial chaperonin GroEL, which binds various hydrophobic substances within its large hydrophobic cavity [27]. The crystal structure of D7r4 revealed that its hydrophobic binding pocket has polar or charged side chains that can form hydrogen bonds with ligand functional groups in varied arrangements, resulting in a broad ligand specificity [15]. Isothermal titration calorimetry, which has been used to measure binding of D7 protein binding to biogenic amines, may be a suitable method for testing the ability of these proteins to bind bendiocarb and pyrethroids [10].

Silencing the expression of D7 proteins yielded only moderate decreases in D7 transcript, which could be due to both transcript abundance and the previously-documented inefficiency of RNAi in mosquito salivary glands [28, 29]. Overexpression of *D7r2* and *D7r4* from a transgene will provide a more robust tool for investigating the role of these proteins in insecticide resistance. In particular, this method would allow us to test the promoter activity of putative D7 regulatory sequences, and test whether the tissue-specificity of D7 expression impacts insecticide resistance.

Whether D7 proteins are directly responsible for insecticide resistance or simply closely-associated markers, their role in mosquito blood-feeding makes this overexpression phenotype relevant to malaria transmission. Real-time quantitative PCR analyses of mosquitoes collected post-IRS detected instances of extremely high levels of *D7r2* and *D7r4* expression. D7 proteins are estimated to compose at least 5–20% of salivary protein [10], thus resistant mosquitoes with even modest levels of overexpression would be expected to have large quantities of these proteins in their saliva. Increased D7 expression may alter the efficiency of blood-feeding, impacting the likelihood of parasite transmission. Previously, knockdown of another D7 gene, *D7L2,* was shown to decrease blood feeding capacity and increase probing time in *An. gambiae* mosquitoes [29]. Furthermore, it was recently reported that a genetically modified D7 knock out *Aedes aegypti* mosquito was significantly less susceptible to an avian malaria parasite, *Plasmodium gallinaceum* [30]. Exploration of blood feeding in D7-overexpressing transgenic mosquitoes will allow for a more precise measurement of feeding success, blood meal size, and probing time. Blood-feeding behaviour in resistant mosquitoes is an important characteristic to measure, as it impacts both their reproductive success and vector competence.

## Conclusions

The replicated association between expression of *D7* genes and bendiocarb resistance across multiple geographic locations and multiple collection years supports the hypothesis that D7 proteins confer insecticide resistance. Further study of the function of these proteins in insecticide resistance, and the impact of their overexpression on mosquito blood-feeding behaviour is needed. This study demonstrates the value of a whole-genome approach to detecting insecticide resistance, as it identified genes that have no known detoxification function and have never before been studied for their role in insecticide resistance. Screening for novel insecticide resistance candidates is necessary, as even the most well-characterized resistance mutations explain only a small fraction of a resistance phenotype [31].

Continued surveillance of insecticide resistance and investigation of the underlying mechanisms are a key component of vector control in malaria-endemic countries. The recent success of the IRS campaign in Nagongera underscores the value of insecticides in malaria control and the importance of preserving their efficacy.

## Methods

### Mosquito collection and insecticide resistance phenotyping

WHO tube bioassays were performed in January, March, and May 2015, as described previously [32]. Briefly, mosquito larvae were collected from various breeding sites using the dipping method, then transferred to an insectary in Nagongera for rearing. Adult mosquitoes were fed on a 10% sugar solution and identified as belonging to the *An. gambiae* species complex using morphological keys. Female mosquitoes were exposed to 0.1% bendiocarb or control papers according to standard WHO tube bioassay protocols [33].

### Whole-genome microarray

For all bendiocarb-resistant and unexposed mosquito samples, chelex-extracted DNA from individual legs was used as template for a species identification PCR [34]. Mosquitoes from the Kisumu laboratory strain were included as fully susceptible control samples. RNA was extracted from pools of five *An. gambiae s.s.* females using an RNAqueous®-4PCR Total RNA Isolation Kit (Ambion) according to the manufacturer’s protocol. For each sample type (Nagongera resistant and unexposed, Kihihi resistant and unexposed, Kisumu), four RNA pools were extracted. A Nanodrop spectrophotometer (Nanodrop Technologies) and an Agilent 2100 Bioanalyser (Agilent Technologies) were used to measure the quality and quantity of RNA samples. RNA labelling, as well as array hybridization, washing, scanning, and feature extraction were performed as previously described [25]. Five 8 × 15 K Agilent whole-transcriptome *An. gambiae* microarray chips (A-MEXP-2196) were used in an interwoven loop design to compare four biological replicates each of resistant, unexposed, and laboratory mosquitoes with maximal statistical power (Additional file 5) [25, 35]. Normalization of microarray data was performed using the Limma package within R software [36]. Analysis of signal intensities was then completed using the MAANOVA package [37]. Four of the forty hybridizations (Kihihi resistant #3 cy5/Kihihi unexposed #3 cy3, Kisumu #3 cy5/Nagongera resistant #4 cy3, Nagongera control #1 cy5/Kihihi resistant #2 cy3, Nagongera control #3 cy5/Kihihi resistant #4 cy3) were excluded from the analysis due to the poor quality of the hybridizations, which yielded aberrant Cy3/Cy5 spectra. Full microarray results are presented in Additional file 6. The results of these analyses were then filtered to identify probes that: 1. Displayed statistical significance (*P* value < 0.05) in an ANOVA *F*-test of the resistant vs. unexposed individuals comparison; 2. Displayed a log_2_ fold change greater than 0.3 in the resistant vs. unexposed individuals comparison; 3. Displayed the greatest fold change in the resistant vs. Kisumu individuals comparison (relative to resistant vs. unexposed and unexposed vs. Kisumu comparisons). All microarray data have been deposited in ArrayExpress (accession no. E-MTAB-6280).

### Ace1 TaqMan

DNA extracted from individual legs was used as template for a TaqMan assay to detect the G119S mutation in the Ace1 gene [38]. Each 10 μl reaction contained 1× Sensimix (Bioline), 1× primer/probe mix and 1 μl template DNA. An Agilent MX3005p real-time PCR machine was used to run the following program: 95 °C for 10 min followed by 40 cycles of 95 °C for 10 s and 60 °C for 45 s. Genotypes were called from endpoint HEX and FAM fluorescence using MxPro software.

### Real-time quantitative PCR

The expression of nine genes was evaluated using qPCR. Following species identification, RNA was extracted from individual females collected in January, March, and May 2015 using a Zymo Quick-RNA MiniPrep kit (Zymo Research) according to the manufacturer’s protocol.

Invitrogen SuperScript III Reverse Transcriptase (Invitrogen) and 200 nanograms of template RNA were used to perform cDNA synthesis reactions. Following cDNA purification with a QIAquick PCR Purification Kit (QIAGEN), triplicate qPCR reactions were prepared using SYBR Brilliant III (Agilent Technologies) and 300 nM each primer (Additional file 7). Primers were designed using the Primer-BLAST tool (http://www.ncbi.nlm.nih.gov/tools/primer-blast/) and evaluated for their efficiency over a ten-fold serial dilution. Primer efficiencies ranged between 89 and 110%, and were included in calculations of fold change. Amplification was performed and analysed using an Agilent Mx3005P QPCR System. The thermal profile was as follows: 1 cycle 95C for 3 min, 40 cycles of 95C for 10 s followed by 60C for 10 s. Gene expression relative to two housekeeping genes (rpS7 and GDPH) was calculated using the REST 2009 software tool [39]. Using this tool, the transcript abundance in each sample was calculated relative to the group of unexposed control samples.

### RNAi-induced gene silencing

Double-stranded RNAs targeting the *D7r2* and *D7r4* genes was synthesized using an Applied Biosystems MEGAscript RNAi kit according to the manufacturer’s protocol. The template for dsD7r2 and dsD7r4 synthesis was PCR-amplified from a mixed sample of Nagongera mosquito cDNA, then cloned using a Thermo scientific CloneJET PCR cloning kit. Kisumu strain mosquitoes age 2–3 days were drawn haphazardly from a colony cage. A Drummond nano-injector was used to inject 101 nL of solution containing 500 nanograms dsD7r2, 1 microgram dsD7r4, or an equivalent amount of dsGFP into the thorax of cold-anaesthetized mosquitoes. The first day post-injection, five males from the colony cage were added to each cage to provide an additional opportunity for mating. Five females were removed from each cage at 24, 48, 72, and 96 h post-injection for RNA extraction. The sequences of primers used in dsRNA synthesis and qPCR analyses of knockdown individuals are listed in Additional file 7. Expression of *D7r1–4* was evaluated using RNA extracted from pools of 5 carcasses.

### Sequencing of D7-coding and adjacent genomic regions

Leg DNA from individual females collected in March and May 2015 was used as template for amplification of the *D7r2*/*D7r4* intergenic region. Forward (5’ GCTACTGAAGGCTGGCAAGA 3′) and reverse (5’ CGGATCTCGCACAGTCTACT 3′) primers were designed to bind within the *D7r2* and *D7r4* coding sequences, respectively. A high-fidelity Taq polymerase, either Thermo Scientific Phusion Hot Start II High-Fidelity DNA polymerase or NEB Q5 High-Fidelity DNA polymerase, was used according to the manufacturer’s protocol. A Promega Wizard SV gel and PCR clean-up system was used to purify individual PCR amplification products. A Thermo Scientific CloneJET PCR cloning kit was used to clone select PCR amplification products prior to sequencing. Assembled sequences of PCR amplification products are presented in Additional file 2.

Leg DNA from individual females collected from Nagongera in 2015 was used as template for amplification of a region downstream of the short D7 cassette. The genomic region encoding a non-coding transcript, identified by Arca et al. as “Contig 709”, was amplified using the following primers: 709F (5’ ACCTATCCATCAGTTCCACCAC 3′) and 709R (5’ CAGGCCTAATCTGTGGCAGT 3′) [12]. PCR products were amplified and cloned as described as above. Assembled sequences of PCR amplification products are presented in Additional file 3.

Leg DNA from individual females collected from Nagongera in 2014 was used as template for amplification of the D7r2 and D7r4 coding sequences. The D7r4 coding region of four resistant and four unexposed females was amplified using the following primers: D7r4seqF (5’ GTAATTCTGAAGATCAAGGTGTG 3′) and D7r4seqR (5’ CGTGCCTTTGAACGCTACAT 3′) or D7r4seqF2 (5’ AGGTAATGGATCATGAAGTAAGTCT 3′) and D7r4seqR2 (5’ TGAACGCTACATCTGTTTTCA 3′). Additional sequences the D7r2/D7r4 intergenic region (described above) was used to assemble partial or full coding sequences. The D7r2 coding region of three resistant females was amplified using the following primers: D7r2seqF (5’ TCGCAGTATAAAAGGCAGTATCT 3′) and D7r2seqR (5’ TGCATCATTGTTCCTTTTGCT 3′). PCR products were amplified and cloned as described as above. Assembled sequences of PCR amplification products are presented in Additional file 1.

### D7 SNP genotyping

A real-time quantitative PCR using a locked nucleic acid probe was designed for genotyping individuals for the 3R:8564156 C/T SNP located in Contig 709. The PCR primers TM709F (5’ GCCCGCGTAATAGGGATTATGT 3′) and TM709R (5’ TTTTTCGCTGGGAAGTTGGTC 3′) were used in combination with the following probes: 709Alt (5’ CGCC+A + T + GGAGA 3′) labelled with FAM, 709Ref (5’ CGCC+A + C + GGAGA) labelled with HEX. Each 10 μl reaction contained 1× Sensimix (Bioline), 0.5 μM each primer, 0.5 μM each probe, and 1 μl template DNA. An Agilent MX3005p real-time PCR machine was used to run the following program: 95 °C for 10 min followed by 40 cycles of 92 °C for 15 s and 57 °C for 1 min. Genotypes were called from endpoint HEX and FAM fluorescence using MxPro software.

### In silico methods

Structure modelling of the D7r2 protein was carried out using the crystal structure of D7r4 protein bound to serotonin (PDB code 2qeh; [15]) as a template. HHpred [40, 41] was used to align the target and template, which shared 32% sequence identity, producing an alignment covering residues 3–146 of the D7r2 protein. MODELLER [42] was used to generate 40 models from this alignment and DOPE scores [43] used to select the best model.

PyMOL (pymol.org) was used for manual positioning of bendiocarb into D7 protein structures. Automated small molecule docking was then done at the ROSIE/Rosetta [16, 17] server. PDBSUM [44] was used to obtain a precalculated definition of the cavity shape and volume of the D7r4 crystal structure, and ProFunc [45] used for similar calculations on model structures.

### Meta-analysis of *Anopheles* genome-wide microarray data

To identify publications containing microarray data collected from insecticide-resistant *Anopheles* mosquitoes, a search was performed on the PubMed website (date of search: December 4, 2016) with the following search terms: Anopheles AND insecticide resistance AND microarray. These 32 results were filtered to select publications in which genome-wide microarrays were performed on whole female mosquitoes that had been selected with insecticides. Three microarrays were used in these publications: the 4 × 44 k (ArrayExpress accession number A-MEXP-2245) and the 8 × 60 k (A-MEXP-2374) microarrays were used to assay *An. funestus* mosquitoes, and the 8 × 15 k (A-MEXP-2196) microarray was used to assay *An. gambiae s.s.* and *An. arabiensis* mosquitoes [25, 46, 47]. Additional searches on Google Scholar using these microarray identification numbers yielded three publications not listed in the PubMed query. A list of the 35 PubMed and Google Scholar search results is provided in Additional file 8.

### Statistical analyses

GraphPad Prism version 5.04 for Windows statistical software (GraphPad Software) was used to perform Fisher’s exact, Mann-Whitney and Spearman tests for statistical significance. The lower and limits of the 95% confidence interval for a proportion were calculated using VassarStats (www.vassarstats.net).

## Additional files

Additional file 1: Partial and complete D7r2 and D7r4 coding sequences, from Nagongera mosquitoes. (DOCX 12 kb)

Additional file 2: FASTA sequences of amplified *D7r2*/*D7r4* intergenic regions, from Nagongera mosquitoes. (DOCX 22 kb)

Additional file 3: FASTA sequences of region encoding Contig 709 transcript, from Nagongera mosquitoes. (DOCX 20 kb)

Additional file 4: Comparison of the cavity binding serotonin in the D7r4 crystal structure (PDB code 2qeh; [15]) and the larger cavity predicted for modelled D7r2, largely due to the replacement of Phe110 and Leu43 with Val residues. The figure was made with PyMOL (pymol.org). (PNG 1766 kb)

Additional file 5: Diagram of hybridizations performed in the microarray analysis. (PDF 117 kb)

Additional file 6: Complete microarray results. (XLSX 7082 kb)

Additional file 7: Sequences of primers used in qPCR analyses and dsRNA synthesis. (XLSX 11 kb)

Additional file 8: Articles considered in the meta-analysis of *Anopheles* insecticide resistance microarrays. (DOCX 20 kb)

## Abbreviations

CDC: Center for disease control
dsRNA: Double-stranded RNA
ESP: Epithelial serine protease
IRS: Indoor residual spraying
LLINs: Long-lasting insecticidal nets
qPCR: Real-time quantitative PCR
SNP: Single nucleotide polymorphism
WHO: World Health Organization
