# Supplementary material for: Genome-wide transcriptional analyses in Anopheles mosquitoes reveal an unexpected association between salivary gland gene expression and insecticide resistance
Source: BMC Genomics. 2018 Mar 27;19:225. doi: 10.1186/s12864-018-4605-1 (PMC5870100; doi:10.1186/s12864-018-4605-1)
Supplement: Supplementary file 1 — Partial and complete D7r2 and D7r4 coding sequences, from Nagongera mosquitoes. (DOCX 12 kb) [file 12864_2018_4605_MOESM1_ESM.docx]

T=Nagongera resistant sample

TC=Nagongera unexposed control sample

D7r4 coding sequences

>T5

ATGAAACGGCAAGTGATTATCAGCTATTTTCTCGCAGTGTGCTTGTTAGCACTCGTGCAGGGTGAAACTGTGCAAGATTGTGAGAATAAGCTGCCACCGTCGCTGAAGAGTAGACTGTGCGAGATCCGGCGGTACGAGATCATCGAGGGACCCGAGATGGACAAGCACATTCATTGCGTGATGCGAGCACTCGACTTTGTCTATGAGGATGGTCGTGGAGATTACCATAAGCTGTACGATCCATTGAACATTATCGAGCTGGACAAAAGACACGATGTGAATCTTGAGAAGTGTATTGGCGAATGCGTACAAGTCCCGACAAGCGAGCGTGCTCACGTGTTCTACAAATGTCTGCTGAAATCAACCACCGGGCGCACGTTCAAGAAGGTGTTCGATCTGATGGAATTGAAAAAGGCTGGCAAAGTGCCACAGCATCAACGGTACACTGCAGAGTTTGTGCAAATCATGAAGGATTATGATAAGGCATTAAACTGCTGA

>T6

ATGATACGGCAAGTGATTATCAGCTATTTTCTCGCAGTGTGCTGCTTAGCACTTGTGCAGAGTGAAACTGTACAAGATTGTGAGAATAAGCTGCCACCGTCGCTGAAGAGTAGACTGTGCGAGATCCGGCGGTATGAGATCATCGAAGGACCGGAGATGGACAAGCACATTCATTGCGTGATGCGAGCACTTGACTTTGTCTATGAGGATGGTCGTGGAGATTACCATAAGCTGTACGATCCATTGAACATTATCGAGCTGGACAAAAGACACGATGTTAATCTTGAGAAGTGTATTGGCGAATGCGTACAAGTCCCGACAACCGAGCGTGCTCACGTGTTCTACAAGTGTCTGCTAAAATCAACCACCGGGCGCACGTTCAAGAAGGTGTTCGATCTGATGGAATTGAAAAAGGCTGGCAAAGTGCCACAGCATCAACGGTACACTGCAGAGTTTGTGCAAATCATGAAGGATTATGATAAGGCATTAAACTGCTGA

>T37

ATGAAACGGCAAGTGATTATCAGCTATTTTCTCGCAGTGTGCTGCTTAGCACTCGTGCAGGGTGAAACTGTGCAAGATTGTGAGAATAAGCTGCCACCGTCGCTGAAGAGTAGACTGTGCGAGATCCGGCGGTACGAGATCATCGAGGGACCGGAGATGGACAAGCACATTCATTGCGTGATGCGAGCACTCGACTTTGTATATGAGGATGGTCGTGGAGATTACCATAAGCTGTACGATCCATTGAACATTATCGAGCTGGACAAAAGACACGATGTGAATCTTGAGAAGTGTATTGGCGAATGCGTACAAGTCCCGACAAGCGAGCGTGCTCACGTGTTCTACAAATGTCTGCTGAAATCAACCACCGGGCGCACGTTCAAGAAGGTGTTCGATCTGATGGAATTGAAAAAGGCTGGCAAAGTGCCACAGCATCAACGGNACACTGCAGAGTTTGTGCAAATCATGAAGGATTATGATAAGGCATTAAACTGCTGA

>T47

TTATCAGCTATTTTCTCGCAGTGTGCTGCTTAGCACTCGTGCAGGGTGAAACTGTACAAGATTGTGAGAATAAGCTGCCACCGTCGCTGAAGAGTAGACTGTGCGAGATCCGGCGGTACGAGATCATCGAGGGACCGGAGATGGACAAGCACATTCATTGCGTGATGCGAGCACTCGACTTTGTCTATGAGGATGGTCGTGGAGATTACCATAAGCTGTACGATCCATTGAACATTATCGAGTTGGACAAAAGACACGATGTGAATCTTGAGAAGTGTATTGGCGAATGCGTACAAGTCCCGACAAGCGAGCGTGCTCACGTGTTCTACAAGTGTCTGCTGAAATCAACCACCGGGCGCACGTTCAAGAAGGTGTTCGATCTGATGGAATTGAAAAAGGCTGGCAAAGTGCCCCAACATCAACGGTACACTGCANAGTTTGTGCAAATCATGAAGGATTATGATAAGGCATNAAACTGCTGA

>TC11

ATGATACGGCAAGTGATTATCAGCTATTTTCTCACAGTGTGCTTGTTAGCACTCGTTCAGGGTGAAACTGTGCAAGATTGTGAGAATAAGCTGCCACCGTCGCTGAAGAGTAGACTGTGCGAGATCCGGCGGTACGAGATCATCGAGGGACCGGAGATGGACAAGCACATTCATTGCGTGATGCGAGCACTCGACTTTGTCTATGAGGATGGTCGTGGAGATTACCATAAGCTGTACGATCCATTGAACATTATCGAGCTGGACAAAAGACACGATGTGAATCTTGAGAAGTGTATTGGCGAATGCGTACAAGTCCCGACAAGCGAGCGTGCTCACGTGTTCTACAAATGTCTGCTGAAATCAACCACCGGGCGCACGTTCAAGAAGGTGTTCGATCTGATGGAATTGAAAAAGGCTGGCAAAGTGCCACAGCATCAACGGTACACTGCAGAGTTTGTGCAAATCATGAAGGATTATGATAAGGCATTAAACTGCTGA

>TC54

ATGAAACGGCAAGTGATTATCAGCTATTTTCTCGCAGTGTGCTGCTTAGCACTCGTGCAGGGTGAAACTGTGCAAGATTGTGAGAATAAGCTGCCACCGTCGCTGAAGAGTAGACTGTGTGAGATCCGCCGGTACGAGATCATCGAAGGACCGGAGATGGACAAGCACATTCATTGCGTGATGCGAGCACTCGACTTTGTCTATGAGGATGGTCGTGGCGATTACCATAAGCTGTACGATCCATTGAACATTATCGAGCTGGACAAAAGACACGATGTGAATCTTGAGAAGTGTATTGGCGAATGCGTACAAGTCCCGACAAGCGAGCGTGCTCACGTGTTCTACAAATGTCTGCTGAAATCAACCACCGGGCGCACGTTCAAGAAGGTGTTCGATCTGATGGAATTGAAAAAGGCTGGCAAAGTGCCACAGCATCAACGGTACACTGCAGAGTTTGTGCAAATCATGAAGGATTATGATAAGGCATTAAACTGCTGA

>TC6

ATGAAACGGCAAGTGATTATCAGCTATTTTCTCGCAGTGTGCTGCTTAGCACTCGTGCAGAGTGAAACTGTACAAGATTGTGAGAATAAGCTGCCACCGTCGCTGAAGAGTAGACTGTGTGAGATCCGCCGGTACGAGATCATCGAAGGACCGGAGATGGACAAGCACATTCTTTGCGTGATGCGAGCACTCGACTTTGTCTATGAGGATGGACGTGTCAATTACCTTAAGCTGAACGATCCATTGAACATTATCGAGCTGGACAATAAACACGATGTGAATCTTGAGAAGTGTATTGCC

>TC9

ATGAAACGGCAAGTGATTATCAGCTATTTTCTCGCAGTGTGCTGCTTAGCACTCGTGCAGGGTGAAACTGTGCAAGATTGTGAGAATAAGCTGCCACCGTCGCTGAAGAGTAGACTGTGCGAGATCCGGCGGTACGAGATCATCGAGGGACCGGAGATGGACAAGCACATTCATTGCGTGATGCGAGCACTCGACTTTGTCTATGAGGATGGTCGTGGAGATTACCATAAGCTGTACGATCCATTGAACATTATCGAGCTGGACAAAAGACACGATGTGAATCTTGAGAAGTGTATTGGCGAATGCGTACAAGTCCCGACAAGCGAGCGTGCTCACGTGTTCTACAAGTGTCTGCTGAAATCAACCACCGGGCGCACGTTCAAGAAGGTGTTCGATCTGATGGAATTGAAAAAGGCTGGCAAAGTGCCACAACATCAACGGTACACTGCAGAGTTTGTGCAAATCATGAAGGATTATGATAAGGCATTAAACTGCTGA

D7r2 coding sequences

>T39

ATGTTCAAGAAACTACTACTGAGCGTTGGACTGGTCTGGTGTTTGATCTCTTTGGGTCAGGCCCGAAAGGAGTCAACGGTGGAGGAGTGCGAGAAAAACATTGGCGATTCGCTGAAGGATCGTGTTTGCGAGCTACGCCAGTACACGCCCGTTAGCAGCGATGACATGGACAAGCACATGCAGTGCGTCCTGGAGGTGGTTGGATTTGTGGATGGAAATGGAGAAGTTAAGGAAAGCGTTCTGCTAGAATTGCTGCAGCGCGTCGACAGTGGCGTCAATCATGCGGCCAACATGAAGAAGTGTGTGACGGAAGCATCGACTTCGGGCAGTGACAAAAAAGCTAACACTTTCTACACGTGCTTTTTGGGTACGAGCTCATTGGCCGGGTTTAAGAATGCGGTCGACTACAACGAGCTACTGAAGGCTGGCAAGATGCAGACGAGCGATCCGTTCGATATGAACCGCGTGGCAGCGCTGATCAAGGAAATCGATGATGGTTTGTGCTAG

>T37

ATGTTCAAGAAACTACTACTGAGCGTTGGACTGGTCTGGTGTTTGATCTCTTTGGGTCAGGCCCGAAAGGAGTCAACGGTGGAGGAGTGCGAGAAAAACATTGGCGATTCGTTGAAGGACCGTGTTTGCGAGCTACGCCAGTACACGCCCGTTAGCAGCGATGACATGGACAAGCACATGCAGTGCGTCCTGGAGGTGGTTGGATTTGTGGATGGAAATGGAGAAGTTAAGGAAAGCGTTCTGCTAGAATTGCTGCAGCGTGTCGACAGTGGCGTCAATCATGCGGCCAACATGAAGAAGTGTGTGACGGAAGCATCGACTTCGGGCAGTGACAAGAAAGCCAACACCTTCTACACGTGCTTTTTGGGNACGAGTTCATTGGCCGGGTTTAAGAATGCGGTCGACTACAACGAGCTACTGAAAGCTGGCAAGATGCAGACGAGCGATCCGTTCGATATGAACCGCGTGGCAGCGCTGATCAAGGAAATCGATGATGGTTTGTGCTAG

>T5

ATGTTCAAGAAACTACTACTGAGCGTTGGACTGGTCTGGTGTTTGATCTCTTTGGGTCAGGCCCGAAAGGAGTCAACGGTGGAGGAGTGCGAGAAAAACATTGGCGATTCGTTGAAGGACCGTGTTTGCGAGCTACGCCAGTACACGCCCGTTAGCAGCGATGACATGGACAAGCACATGCAGTGCGTCCTGGAGGTGGTTGGATTTGTGGATGGAAATGGAGAAGTTAAGGAAAGCGTTCTGCTAGAATTGCTGCAGCGCGTCGACAGTGGCGTCAATCATGCGGCCAACATGAAGAAGTGTGTGACGGAAGCATCGACTTCGGGCAGTGACAAGAAAGCCAACACCTTCTACACGTGCTTTTTGGGTACGAGCTCATTGGCCGGGTTTAAGAATGCGGTCGACTACAACGAGCTACTGAAGGCTGGCAAGATGCAGACTAGCGATCCGTTCGATATGAACCGTGTGGCAGCGCTGATCAAGGAAATCGATGATGGTTTGTGCTAG
