# Supplementary material for: Genome-wide transcriptional analyses in Anopheles mosquitoes reveal an unexpected association between salivary gland gene expression and insecticide resistance
Source: BMC Genomics. 2018 Mar 27;19:225. doi: 10.1186/s12864-018-4605-1 (PMC5870100; doi:10.1186/s12864-018-4605-1)
Supplement: Supplementary file 2 — FASTA sequences of amplified D7r2/D7r4 intergenic regions, from Nagongera mosquitoes. (DOCX 22 kb) [file 12864_2018_4605_MOESM2_ESM.docx]

T=Nagongera resistant sample

TC=Nagongera unexposed control sample

TC14-T17 low expression, TC50-T2 high expression

| sample | Fold change D7r4 expression vs. unexposed controls | sequences |
| --- | --- | --- |
| TC14 | 1 | 2 |
| TC32 | 1 | 1 |
| TC55 | 1 | 1 |
| T13 | 2 | 2 |
| T17 | 2 | 1 |
| TC50 | 4 | 2 |
| T14 | 5 | 2 |
| T4 | 5 | 2 |
| T10 | 6 | 2 |
| T16 | 7 | 2 |
| T18 | 8 | 2 |
| T11 | 8 | 2 |
| T7 | 9 | 2 |
| TC8 | 10 | 2 |
| T12 | 12 | 2 |
| T1 | 34 | 2 |
| T2 | 216 | 1 |

>T11S

CGTGTGGCTGCGCTGATCAAGGAAATCGATGATGGTTTGTGCTAGTAGAT

TGATTGAACATGTTTAAAGCAAAAGGAACAATGATGCAATACAAATAAAA

TATAGAGCAAGCAAAACAAATTATGGACTATTTGTGTGTTAAGTTGTGCG

TGACTTTGGGAAATGAGGGTCAGTGGATAAGAAAGATTGTGAAATTTGGG

TTCTTGAAAGAAGATCGTTAGCTATGGAGTTACCAGATGACGTAACTTGT

TGGGTACCACAAGATAGAACTTGAACCATAATTTCAAGATGCCTGGACTG

ATTCATATGTCTGCTCTTGTAACATTAATAGTACAACCCTCATCTTCATT

ACCTTAATGTCAAATTAAACTTCATCGATCGCGTTTAACACCATATGAAC

AACCTATGTCGGATCATTCAAGTTAATGTGTATTGGGCAGTCTTGTGGTA

TAGCTGTCAACTCGCTCGACTTAACAACATGACCGTCGTGGGTTCAAGCC

TCATATGGAACGCTCTACGTAGCAAGGAGTGAGTATTCAGCTGTGTGATA

ATTAACAATAAGTATCGAACGCCTACATAAACCGGTTAAAATAGGGTTTT

TATTAAAGGATTTGTGATTTTTCTTTATATTATTAAGCTAGCTGGCTTGA

CAAATTCTGTACTCAACTACTTTTTACTGTGATATGATAAACGAGTGTAT

AATAGATTCCATATAATGAAAAAGAATAAAAATTTAAGTCACAGATATCC

GGAAAGCAGTGTTTTCTGATGCTATTTTTAATTCTTTACGGTTTATAGAA

TATTACAAATAAAACATCATGTATGATGGTGTTCAGACAGTAGTTTTCTA

ACACTAATGATTAGAACAGCTATTTAAGTTCAATATACTATATATGTTTT

TATATTTAAATAACACGTTTGCTCACCTTTGCAAATATTACACGATAAAT

GCTATATCGATACAACGCAACTGGTTTTCCAATCCGATTGTCAATGGGTA

AACAAGATACTAGAATATATAAGCATAAATTACTACCGATCAGCCAAACA

ACAATCCTGAAGATCAAGGTGTGACATCATGAAACGGCAAGTGATTATCA

GCTATTTTCTCGCAGTGTGCTGCTTAGCACTCGTGCAGGTAATTTATCGT

CAAGTATGTGCTTTGTTAGAAATTGCAAATACAATTATTTTCCCGCAGGG

TGAAACTGTACAAGAT

>T13S

GTGGCAGCGCTGATCANGAAATCGATGATGGTTTGTGCTAGTAGATTGAT

TGAACTTGTTTAAAGCAAAAGGAACAATGATGCAATACAAATAAAATATA

GAGCAAGCAAAACAAATTATGGACTTTTTGTGTGTTAAGTTGTGCGTGAC

TTTGGGAAATGAGGGTCAGTGGATAAGAAAGATTGTGAAATTTGGGTTCT

TGAAAGAAGATCGTTAGCTATGGAGTTACCAGATGACGTAACTTGTTGGG

TACCACAAGATAGAACTTGAACCATAATTTCAAGATGCCTGGACTGATTC

ATATGTCTGCTCTTGTAACATTAATAGTACAACCCTCATCTTCATTACCT

TAATGTCAAATTAAACTTCATCGATCGCGTTTAACACCATATGAACAACC

TATGTCGGATCATTCAAGTTAATGTGTATTGGGCAGTCTTGTGGTATAGC

TGTCAACTCGCTCGACTTAACAACATGACCGTCGTGGGTTCAAGCCTCAT

ATGGAACGCTCTACGTAGCAAGGAGTGAGTATTCAGCTGTGTGATAATTA

ACAATAAGTATCGAACGCCTACATAAACCGGTTAAAATAAGGTTTTTTTT

AAAGGATTTGTGATTTTTCTTTATGTTATTAAGCTAGCTGGCTTGACAAA

TTCTGTACTCAACTACTGTTTACTGTGATACGATAAACCTTCGAGTGTAT

AATAGATTCCATATAATAAAAAAGAATAAAAATTTAAGTCACAGATATCC

AGAAAGCAGTGTTTTCTGATGCTATTTTTAATTCTTTACGGTTTATAGAA

TATTAAAAAAATAAAACATCATGTAGGATGGTGTTCAGACAGCAGTTTTC

TAACACTAATGATTAGAACAGCTGTTTAAGTTCAATATACTATATATGTT

TTTATATTTAAATAACACGTTTGCTCACCTCTGCAAATATTACACGATAA

ATGCTATATTGATACAAGGCAAATGGTTTTCCAATCCGATTGTCAATGGG

TAAACAAGATACAAGAATATATAAGTATAAATTACTACTGATCATTTAAA

CAATAATTCTGAAGATCAAGGTGTGACATCATGATACGGCAAGTGATTAT

TAGCTATTTTCTCGCAGTGTGCTTGCTAGCACTCGTGCAGGTAATTTATC

GTCAAGTATGTGCTTTGCTAGAAATTACAAATACANTTATTTTCCCGCAG

AGTGAAACTGTACAAGATGG

>T12L

TGACGTGTGGCAGCGCTGATCAAGGAAATCGATGATGGTTTGTGCTAGTA

GATTGGATGAACATGTTTAAAGCAAAAGGAACAATGATGCAATACAAATA

AAATATAGAGCAAGCAAAACAAATTATGGACTTTTTGTGTGTTAAGTTGC

GCGTGACTTTGGGAAATGAGGGAAGTGGATAAGAAAGATTGTGAAATTTG

GGTTCTTGAAAGAAGATCGTTAGCTATGGAGTTACCAGATGACGTAACTT

GGTGGGTTCCACAAGATAGAACTTGAACCATAATTTCAAGATGCCTGGAC

TGATTCATATGTTAGCTCTCACAAGATTAATAGTACAACTCTCATCTTCA

TTACCTCAATGTCCATTTAAACTTCATCGATCGCGTTTAACACCATATAA

ACAACCTATGTCGGATAATTCAAGTTAATATGTATTGGGCAGTTTTTGTG

GTACAGTTGTCAACTCGCTTGATTTAACAACATGCCCATCATGGGTTCAA

GCCTTATCTGGACCGCTCTACGATTTGTGGTTTCTTTATATAATTAAGCT

AGCTGGCTTGACAAATTCTGTACTCAACTACTTTTTACTGTGATACGATA

AAGTAGTCGAGTGTATAATATATTCCATATAATAAAAAAGAATAAAAATT

TAAGTCACAGCTATCCAGAAAGCAGTGTTTTCTGATGATATTTTTAATTC

TTTACGGTTTATAGAATATTGCAAATAAAACATCATGTAGGATGGCGTTC

AGACAGCAGTTTTCTAACACTAATGATTAGAACAGCTGTTGAAGTTCAAT

ATACTATATATGTTTTTATATTTAAATAACACGTTTGCTCACCTCTGCAA

ATATTACACGATAAATGCTATATTGATACAAGGTAAGTGGTTTTCCAATC

CGATTGTCAATGGGTAAACAAGATACTAGAATATATAAGTATAAATTACT

ACTGATCAGTTAAACAATAATTCTGAAGAGCAAGGTGTGACATCATGATA

CGGCAAGTGATTATCAGCTATTTTCTCACAGTGTGCTTGTTAGCACTCGT

TCAGGTAATGGATCATGAAGTAAGTCTTTATTAGAAATTACAAATACAAT

TATTTTCATGCAGGGTGAGACTGTGCAAGATGTGAGAATAAGCTGCCACC

G

>TC14S

GTGGCAGCGCTGATCAAGGAAATCGATGATGGTTTGTGCTAGTAGATTGG

ATGAACATGTTTAAAGCAAAAGGAACAATGATGCAATACAAATAAAATAT

AGAGCAAGCAAAACAAATTATGGACTTTTTGTGTGTTAAGTTGTGCGTGA

CTTTGGGAAATGAGGGAAGTGGATAAGAAAGATTGTGAAATTTGGGTTCT

TGAAAGAAGATCGTTAGCTATGGAGTTACCAGATGACGTAGCTTGTTGGG

TTCCACAAGATAGAACTTGAACCATAATTTCAAGATGCCTGGACTGATTC

ATATGTTAGCTCTCACAAGATTAATAGTACAACTCTCATCTTCATTACCT

CAATGTCCATTTAAACTTCATTGATCGCGTTTAACACCATATAAACAACC

TATGTCGGATAATTCAAGTTAATATGTATTGGGCAGTTTTGTGGTACAGT

TATCAACTCGCTTGATTTAACAACATGCCCATCATGGGTTCAAGCCTTAT

CTGGACCGCTCTACGATTTGTGGTTTCTTTATATAATTAAGCTAGCTGGC

TTGACAAATTCTGTACTCAACTACTTTTTACTGTGATACGATAAAGTAGT

CGAGTGTATAATATATTCCATATAATAAAAAAGAATAAAAATTTAAGTCA

CAGCTATCCAGAAAACAGTGTTTTCTGATGATATTTTTAATTCTTTACGG

TTTATAGAATATTGCAAATAAAACATCATGTAGGATGGCGTTCAGACAGC

AGTTTTCTAACACTAATGATTAGAACAGCTGTTGAAGTTCAATATACTAT

ATATGTTTTTATATTTAAATAACACGTTTGCTCACCTCTGCAAATATTAC

ACGATAAATGCTATATTGATACAAGGCAAGTGGTTTTCCAATCCGATTGT

CAATGGGTAAACAAGATACTAGAATATATAAGTATAAATTACTACTGATC

AGTTAAACAATAATTCTGAAGAGCAAGGTGTGACATCATGATACGGCAAG

TGATTATCAGCTATTTTCTCACAGTGTGCTTGTTAGCACTCGTTCAGGTA

ATGGATCATGAAGTAAGTCTTTATTAGAAATTACAAATACAATTATTTTC

ATGCAGGGTGAGACTGTGCAAAATGTGAGAATAAGCTGCCACCG

>TC8S

TGTGGCAGCGCTGATCAAGGAAATCGATGATGGTTTGTGCTAGTAGATTG

GATGAACATGTTTAAAGCAAAAGGAACAATGATGCAATACAAATAAAATA

TAGAGCAAGCAAAACAAATTATGGACTTTTTGTGTGTTAAGTTGCGCGTG

ACTTTGGGAAATGAGGGAAGTGGATAAGAAAGATTGTGAAATTTGGGTTC

TTGAAAGAAGATCGTTAGCTATGGAGTTACCAGATGACGTAACTTGTTGG

GTTCCACAAGATAGAACTTGAACCATAATTTCAAGATGCCTGGACTGATT

CATATGTAAGCTCTCACAAGATTAATAGTACAACTCTCATCTTCATTACC

TCAATGTCCATTTAAACTTCATCGATCGCGTTTAACACCATATAAACAAC

CTATGTCGGATAATTCAAGTTAATATGTATTGGGCAGTTTTTGTGGTACA

GTTGTCAACTCGCTTGATTTAACAACATGCCCATCATGGGTTCAAGCCTT

ATCTGGACCGCTCTACGATTTGTGGTTTCTTTATATAATTAAGCTAGCTG

GCTTGACAAATTCTGTACTCAACTACTTTTTACTGTGATACGATAAAGTA

GTCGAGTGTATAATATATTCCATATAATAAAAAAGAATAAAAATTTTAGT

CACAGCTATCCAGAAAGCAGTGTTTTCTGATGATATTTTTAATTCTTTAC

GGTTTATAGAATATTGCAAATAAAACATCATGTAGGATGGCGTTCAGACA

GCAGTTTTCTAACACTAATGATTAGAACAGCTGTTTAAGTTCAATATACT

ATATATGTTTTTATATTTAAATAACACGTTTGCTCACCTCTGCAAATATT

ACACGATAAATGCTATATTGATACAAGGCAAGTGGTTTTCCAATCCGATT

GTCAATGGGTAAACAAGATACTAGAATATATAAGTATAAATTACTACTGA

TCAGTTAAACAATAATTCTGAAGAGCAAGGTGTGACATCATGATACGGCA

AGTGATTATCAGCTATTTTCTCACAGTGTGCTTGTTAGCACTCGTTCAGG

TAATGGATCATGAAGTAAGTCTTTATTAGAAATTACAAATACAATTATTT

TCATGCAGAGTGAGACTGTGCAAG

>T4S

GCAGATGCAGACGAGCGATCCGTCGATATGAACCGTGTGGCAGCGCTGAT

CAAGGAAATCGATGATGGTTTGTGCTAGTAGATTGAATGAACATGTTTAA

AGCAAAAGGAACAATGATGCAATACAAATAAAATATAGAGCAAGCAAAAT

CAATTATGGACTTTTTGTGTGTAAAGTTGCGCGTGACTTTGGGAAATGAG

GGTCAGTGGATAAGTAAGATTGTGATATTTGGGTTCTTGAAAGAAGATCG

TTAGCTATGGAGTTGCCAGATGACGTAACTTGTTGGGTTCCACAAAATAG

AACTTGAATCATTTCAAGATGCCTGGACTGATTCATATGTTAGCTCTCAC

AAGATTAATAGTACAACTCTCATCTTCATTACCTCAATGTCCATTTAAAC

TTCATCGATCGCGTTTAACACCATATAAACAACCTATGTCGGATAATTCA

AGTTAATATGTATTGGGCAGTTTTGTGGTACAGTTGTCAACTCGCTTGAT

TTAACAACATGCCCATCATGGGTTCAAGCCTTATCTGGACCGCTCTACGA

TTTGTGGTTTCTTTATATTATTAAGCTAGCTGGCTTGACAAATTCTGTAC

TCAACTACTTTTTACTGTGATACGATAAAGTAGTCGAGTGTATAATATAT

TCCATATAATAAAAAAGAATAAAAATTTTAGTCACAGCTATCCAGAAAGC

AGTGTTTTCTGATGATATTTTTAATTCTTTACGGTTTATAGAATATTGCA

AATAAAACATCATGTAGGATGGCGTTCAGACAGCAGTTTTCTAACACTAA

TGATTAGAACAGCTGTTTAAGTTCAATATACTATATATGTTTTTATATTT

AAATAACACGTTTGCTCACCTCTGCAAATATTACACGATAAATGCTATAT

ACAAGGCAAGTGGTTTTCCAATCCGATTGTCAATGGGTAAACAAGATACT

AGAATATATAAGTATAAATTACTACTGATCAGTTAAACAATAATTCTGAA

GAGCAAGGTGTGACATCATGATACGGCAAGTGATTATCAGCTATTTTCTC

ACAGTGTGCTTGTTAGCACTCGTTCAGGTAATGGATCATGAAGTAAGTCT

TTATTAGAAATTACAAATACAATTATTTTCATGCAGAGTGAGACTGTGCA

AGA

>T2

GCTACTGAAGGCTGGCAAGATGCAGACGAGCGATCCGTTCGATATGAACC

GTGTGGCAGCGCTGATCAAGGAAATCGATGATGGTTTGTGCTAGTAGATT

GGATGAACATGTTTAAAGCAAAAGGAACAATGATGCAATACAAATAAAAT

ATAGAGCAAGCAAAACAAATTATGGACTTTTTGTGTGTTAAGTTGCGCGT

GACTTTGGGAAATGAGGGTCAGTGGATAAGTAAGATTATGATATTTGGGT

TCTTGAAAGAAGATCGTTAGCTATGGAGTTGCCAGATGACGTAACTTGTT

GGGTGCCACAAAATAGAACTTGAATCATTTCAAGATGCCTGGACTGATTC

ATATGTTAGCTCTCACAAGATTAATAGTACAACTCTCATCTTCATTACGT

CAATGTCAATTTAAACTTCATTGATCGCGTTTAACACCATACAAACAACC

TATGTCGGATAATTCAAGTAATATGTATTGGGCAGTCTTGTGGTACAGCT

GTCAACTCGCTCGACTTAACAACATGCCCATCATGGGTTCAAGCCTCATA

TGGAACGCTCTACGTAGCAAGGAGTGAGTATTCAGCTGTGTGAAAATTAA

CAATAAGTATCGAACGCCTACATAAACCGGTTAAAATAAGGTTTTTTTTA

AAGGATTTGTGGTTTTTCTTTATATTATTAAGTTAGCTGGCTTGACAAAT

TCTGTACTCAACTACTTTTTACTGTGATGCGATAAACCTTCGAGTGTATA

ATATATTCCATATAATAAAAAAAAATAAAAATTTTAGTCACAGCTATCCA

GAAAGCAGTGTTTTCTGATGATATTTTTAATTCTTTACGGTTTATAGAAT

ATTGCAAATAAAACATCATGTAGGATGGCGTTCAGACAGCAGTTTTCTAA

CACTAATGATTAGAACAGCTGTTTAAGTTCAATATACTATATATGTTTTT

ATATTTAAATAACACGTTTGCTCACCTCTGCAAATATTACACGATAAATG

CTATATTGATACAAGGCAAGTGGTTTTCCAATCCGATTGTCAATGGGTAA

ACAAGATACTAGAATATATAAGTATAAATTACTACTGATCAGTTAAACAA

TAATTCTGAAGAGCAAGGTGTGACATCATGATACGGCAAGTGATTATCAG

CTATTTTCTCACAGTGTGCTTGTTAGCACTCGTTCAGGTAATGGATCATG

AAGTAAGTCTTTATTAGAAATTACAAATACAATTATTTTCATGCAGAGTG

AGACTGTGCAAGATTGTGAGAATAAGCTGCCACCGTCGCTGAAGAGTAGA

CTGTGCGAGATCCG

>TC32

TGACGTGTGGCAGCGCTGATCAAGGAAATCGATGATGGTTTGTGCTAGTA

GATTGGATGAACATGTTTAAAGCAAAAGGAACAATGATGCAATACAAATA

AAATATAGAGCAAGCAAAACAAATTATGGACTTTTTGTGTGTTAAGTTGC

GCGTGACTTTGGGAAATGAGGGTCAGTGGATAAGTAAGATTATGATATTT

GGGTTCTTGAAAGAAGATCGTTAGCTATGGAGTTGCCAGATGACGTAACT

TGTTGGGTGCCACAAAATAGAACTTGAATCATTTCAAGATGCCTGGACTG

ATTCATATGTTAGCTCTCACAAGATTAATAGTACAACTCTCATCTTCATT

ACGTCAATGTCAATTTAAACTTCATTGATCGCGTTTAACACCATACAAAC

AACCTATGTCGGATAATTCAAGTAATATGTATTGGGCAGTCTTGTGGTAC

AGCTGTCAACTCGCTCGACTTAACAACATGCCCATCATGGGTTCAAGCCT

CATATGGAACGCTCTACGTAGCAAGGAGTGAGTATTCAGCTGTGTGAAAA

TTAACAATAAGTATCGAACGCCTACATAAACCGGTTAAAATAAGGTTTTT

TTTAAAGGATTTGTGGTTTTTCTTTATATTATTAAGTTAGCTGGCTTGAC

AAATTCTGTACTCAACTACTTTTTACTGTGATGCGATAAACCTTCGAGTG

TATAATATATTCCATATAATAAAAAAAAATAAAAATTTTAGTCACAGCTA

TCCAGAAAGCAGTGTTTTCTGATGATATTTTTAATTCTTTACGGTTTATA

GAATATTGCAAATAAAACATCATGTAGGATGGCGTTCAGACAGCAGTTTT

CTAACACTAATGATTAGAACAGCTGTTTAAGTTCAATATACTATATATGT

TTTTATATTTAAATAACACGTTTGCTCACCTCTGCAAATATTACACGATA

AATGCTATATTGATACAAGGCAAGTGGTTTTCCAATCCGATTGTCAATGG

GTAAACAAGATACTAGAATATATAAGTATAAATTACTACTGATCAGTTAA

ACAATAATTCTGAAGAGCAAGGTGTGACATCATGATACGGCAAGTGATTA

TCAGCTATTTTCTCACAGTGTGCTTGTTAGCACTCGTTCAGGTAATGGAT

CATGAAGTAAGTCTTTATTAGAAATTACAAATACAATTATTTTCATGCAG

AGTGAGACTGTGCAAGAT

>T16S

TGACGCGTGGCAGCGCTGATCAAGGAAATCGATGATGGTTTGTGCTAGTA

GATTAGTTTAAACACATGTTTAAAGCAAAAGGAACAATGATGCAATACAA

ATAAAATATAGAGCAAGCAAAACAAATTATGGACTTTTTGTGTGTAAAGT

TGCGCGTGACTTTGGGAAATGAGGGTCAGTGGATAAGTAAGATTGTGATA

TTTGGGTTCTTGAAAGAAGATCGTTAGCTATGGAGTTGCCAGATGACGTA

ATTTGTTGGGTTCCACAAAATAGAACTTGAATCATTTCAAGATGCCTGGA

CTGATTCATATGTTAGCTCTCACAAGATTAATAGTACAACTCTCATCTTC

ATTACCTCAATGTCCATTTAAACTTCATCCATCGCGTTTAACACCATATA

AACAACCTATGTCGGATAATTCAAGTTAATATGTATTGGGCAGTTTTGTG

GTACATTTGTCAACTCGCTTGATTTAACAACATGCCCATCATGGGTTCAA

GCTTTATCTGGACCGCTCTACGATTTGTGGTTTCTTTATATTATTAAGCT

AGCTGGCTTGACAAATTCTGTACTCAACTAATTTTTACTGTGATACGATA

AAGTAGTCGAGTGTATAATATATTCCATATAATAAAAAAGAATAAAATTT

AAGTCACAGCTATCCAGAAAGCAGTGTTTTCTGATGATATTTTTAATTCT

TTACGGTTTATAGAATATTGCAAATAAAACATCATGTAGGATGGCGTTCA

GACAGCAGTTTTCTAACACTAATGATTAGAACAGCTGTTGAAGTTCAATA

TACTATATATGTTTTTATATTTAAATAACACGTTTGCTCACCTCTGCAAA

TATTACACGATAAATGCTATATTGATACAAGGCAAGTGGTTTTCCAATCC

GATTGTCAATGGGTAAACAAGATACTAGAATATATAAGTATAAATTACTA

CTGATCAGTTAAACAATAATTCTGAAGAGCAAGGTGTGACATCATGATAC

GGCAAGTGATTATCAGCTATTTTCTCACAGTGTGCTTGTTAGCACTCGTT

CAGGTAATGGATCATGAAGTAAGTCTTTATTAGAAATTACAAATACAATT

ATTTTCATGCAGGGNGAGACTGTGCAAG

>T7XL

GCTACTGAAGGCTGGCAAGATGCAGACGAGCGATCCGTTCGATATGAACC

GTGTGGCAGCGCTGATTAAGGAAATCGATGATGGTTTGTGCTAGTAGATT

GGTTGAACATGTTTAAAGCAAAAGGAACAATGATGCAATACAAATAATGT

ATAGAGCAAGCAAAACAGATTATGAACTTTTTTTGTCTTAAGTTGTGCGT

GACCTTAGAAATTGAGGGTCAGTGGATAGGAAAGATTGTGATATTTGGTT

GGGAAAGAAGATCGTTAGCTATGGAGTTGCCAGATGACGAAACTCTTTGG

GTTCCACAAGATAGAACTTGAATAATTTCAAGATGCCTGGACTGATTCAT

ATGTTAGCTCTCGCAAGATTAATAGTACAACTCTCATCTTCATTACCTCA

ATGTCCATTTAAACTTCATTGATCACGTATAACACCATATAAACAACCTA

TGTCGGATCATTCAAGTTAATGTGTATTGGGCAGTCTTGTGATACAGCTG

TCAACTCGCTCGACATAACAACATAACCGTCGTAGGTTCAAGCCTCATAT

GAACCGCCCAACGCAGCAAGGATTGAGTATCCTGCTGTGTGGCAAATAAA

CATAATGTAAACAAATAAACATAATGCAAGCATAATAATGATGTTTTAAT

AACAAGTCTCGAAAGCCTAGCTAGACCGCATAGGTCGCTACGCAAAATAG

AAAAATAAGGGTTTTTAAAAAGGATTTGAGGTTTTTCCTTATATTATTAA

GCTAGCTGGCTTCATAAATTCTGTACTCAATTACTTTTTACTGTGATATG

ATAAACCTTCGAGTGTATAATAGATTCCATATAATGAAAAAGAATAAAAA

ATTAAGTCACAGACATCCAGAACGTAGTGTTTTCTGATATTAGGTTTAAT

TCTTAAGGGTTTATAGAATATTACAAATGAAACATCGTGTAGGATGGCGT

TCAGATCGCAGCTTTCTTACACTAATGATTGGAACAGCTGTTTAATTTCA

ATATACCATATATTTTTTTATATTTAAACAACTTATTTGCTTACCTCTGC

AAATATTACACAATAAATTCAATATTGATACAATGCAAGTGGTTTTCCAA

TCCGATTGTTAAGGGGTAAACAAGATACTAGAATATATAAGCATAAATTA

CTACCGGTCAGCCAAACAACAATCCTGAAGATCAAGGTGTGACATCATGA

AACGGCAAGTGATTATCAGCTATTTTCTCGCAGTGTGCTTGTTAGCACTC

GTGCAGGTAATTTATCGTCAAGCATGTGCTTTGTTAGAAATTGCAAATAC

AATTATTTTCCCGCAGGGTGAAACTGTGCAAGATTGTGAGAATAAGCTGC

CACCGTCGCTGAAGAGTAGACTGTGCGAGATCCG

>TC14L

ACGTGTGGCAGCGCTGATTAAGGAAATCGATGATGGTTTGTGCTAGTAGA

TTGGTTGAACATGTTTAAAGCAAAAGGAACAATGATGCAATACAAATAAT

GTATAGAGCAAGCAAAACAGATTATGAACTTTTTTTGTCTTAAGTTGTGC

GTGACCTTAGAAATTGAGGGTCAGTGGATAGGAAAGATTGTGATATTTGG

TTGGGAAAGAAGATCGTTAGCTATGGAGTTGCCAGATGACGAAACTCTTT

GGGTTCCACAAGATAGAACTTGAATAATTTCAAGATGCCTGGACTGATTC

ATATGTTAGCTCTCGCAAGATTAATAGTACAACTCTCATCTTCATTACCT

CAATGTCCATTTAAACTTCATTGATCACGTATAACACCATATAAACAACC

TATGTCGGATCATTCAAGTTAATGTGTATTGGGCAGTCTTGTGATACAGC

TGTCAACTCGCTCGACATAACAACATAACCGTCGTAGGTTCAAGCCTCAT

ATGAACCGCCCAACGCAGCAAGGATTGAGTATCCTGCTGTGTGGCAAATA

AACATAATGTAAACAAATAAACATAATGCAAGCATAATAATGATGTTTTA

ATAACAAGTCTCGAAAGCCTAGCTAGACCGCATAGGTCGCTACGCAAAAT

AGAAAAATAAGGGTTTTTAAAAAGGATTTGAGGTTTTTCCTTATATTATT

AAGCTAGCTGGCTTCATAAATTCTGTACTCAATTACTTTTTACTGTGATA

TGATAAACCTTCGAGTGTATAATAGATTCCATATAATGAAAAAGAATAAA

AAATTAAGTCACAGACATCCAGAACGTAGTGTTTTCTGATATTAGGTTTA

ATTCTTAAGGGTTTATAGAATATTACAAATGAAACATCGTGTAGGATGGC

GTTCAGATCGCAGCTTTCTTACACTAATGATTGGAACAGCTGTTTAATTT

CAATATACCATATATTTTTTTATATTTAAACAACTTATTTGCTTACCTCT

GCAAATATTACACAATAAATTCAATATTGATACAATGCAAGTGGTTTTCC

AATCCGATTGTTAAGGGGTAAACAAGATACTAGAATATATAAGCATAAAT

TACTACCGGTCAGCCAAACAACAATCCTGAAGATCAAGGTGTGACATCAT

GAAACGGCAAGTGATTATCAGCTATTTTCTCGCAGTGTGCTTGTTAGCAC

TCGTGCAGGTAATTTATCGTCAAGCATGTGCTTTGTTAGAAATTGCAAAT

ACAATTATTTTCCCGCAGGGNGAAACTGTGCAAGA

>T10L

TGACGTGTGGCAGCGCTGATTAAGGAAATCGATGATGGTTTGTGCTAGTA

GATTGGTTGAACATGTTTAAAGCAAAAGGAACAATGATGCAATACAAATA

ATCTATAGAGCAAGCAAAACAGATTATGAACTTTTTTTGTGTTAAGTTGT

GCGTGACCTTAGAAATTGAGGGTCAGTGGATAGGAAAGATTGTGATATTT

GGTTGGGAAAGAAGATCGTTAGCTATGGAGTTGCCAGATGACGAAACTCT

TTGGGTTCCACAAGATAGAACTTGAATAATTTCAAGATGCCTGGACTGAT

TCATATGTTAGCTCTCGCAAGATTAATAGTACAACTCTCATCTTCATTAC

CTCAATGTCCATTTAAACTTCATTGATCACGTATAACACCATATAAACAA

CCTATGTCGGATCATTCAAGTTAATGTGTATTGGGCAGTCTTGTGATACA

GCTGTCAACTCGCTCGACATAACAACATAACCGTCGTAGGTTCAAGCCTC

ATATGAACCGCCCAACGCAGCAAGGATTGAGTATCCTGCTGTGTGGCAAA

TAAACATAATGTAAACAAATAAACATAATGCAAGCATAATAATGATGTTT

TAATAACAAGTCTCGAAAGCCTAGCTAGACCGCATAGGTCGCTACGCAAA

ATAGAAAAATAAGGGTTTTTAAAAAGGATTTGAGGTTTTTCCTTATATTA

TTAAGCTAGCTGGCTTCATAAATTCTGTACTCAATTACTTTTTACTGTGA

TATGATAAACCTTCGAGTGTATAATAGATTCCATATAATGAAAAAGAATA

AAAAATTAAGTCACAGACATCCAGAACGTAGTGTTTTCTGATATTAGGTT

TAATTCTTAAGGGTTTATAGAATATTACAAATGAAACATCGTGTAGGATG

GCGTTCAGATCGCAGCTTTCTTACACTAATGATTGGAACAGCTGTTTAAT

TTCAATATACCATATATTTTTTTATATTTAAACAACTTATTTGCTTACCT

CTGCAAATATTACACAATAAATTCAATATTGATACAATGCAAGTGGTTTT

CCAATCCGATTGTTAAGGGGTAAACAAGATACTAGAATATATAAGCATAA

ATTACTACCGGTCAGCCAAACAACAATCCTGAAGATCAAGGTGTGACATC

ATGAAACGGCAAGTGATTATCAGCTATTTTCTCGCAGTGTGCTTGTTAGC

ACTCGTGCAGGTAATTTATCGTCAAGCATGTGCTTTGTTAGAAATTGCAA

ATACAATTATTTTCCCGCAGGGNGAAACTGTGCAAG

>T13L

CGTGTGGCAGCGCTGATTAAGGAAATCGATGATGGTTTGTGCTAGTAGAT

TGGTTGAACATGTTTAAAGCAAAAGGAACAATGATGCAATACAAATAATC

TATAGAGCAAGCAAAACAGATTATGAACTTTTTTTGTGTTAAGTTGTGCG

TGACCTTAGAAATTGAGGGTCAGTGGATAGGAAAGATTGTGATATTTGGT

TGGGAAAGAAGATCGTTAGCTATGGAGTTGCCAGATGACGAAACTCTTTG

GGTTCCACAAGATAGAACTTGAATAATTTCAAGATGCCTGGACTGATTCA

TATGTTAGCTCTCGCAAGATTAATAGTACAACTCTCATCTTCATTACCTC

AATGTCCATTTAAACTTCATTGATCACGTATAACACCATATAAACAACCT

ATGTCGGATCATTCAAGTTAATGTGTATTGGGCAGTCTTGTGATACAGCT

GTCAACTCGCTCGACATAACAACATAACCGTCGTAGGTTCAAGCCTCATA

TGAACCGCCCAACGCAGCAAGGATTGAGTATCCTGCTGTGTGGCAAATAA

ACATAATGTAAACAAATAAACATAATGCAAGCATAATAATGATGTTTTAA

TAACAAGTCTCGAAAGCCTAGCTAGACCGCATAGGTCGCTACGCAAAATA

GAAAAATAAGGGTTTTTAAAAAGGATTTGAGGTTTTTCCTTATATTATTA

AGCTAGCTGGCTTCATAAATTCTGTACTCAATTACTTTTTTACTGTGATA

TGATAAACCTTCGAGTGTATAATAGATTCCATATAATGAAAAAGAATAAA

AAATTAAGTCACAGACATCCAGAACGTAGTGTTTTCTGATATTAGGTTTA

ATTCTTAAGGGTTTATAGAATATTACAAATGAAACATCGTGTAGGATGGC

GTTCAGATCGCAGCTTTCTTACACTAATGATTGGAACAGCTGTTTAATTT

CAATATACCATATATTTTTTTATATTTAAACAACTTATTTGCTTACCTCT

GCAAATATTACACAATAAATTCAATATTGATACAATGCAAGTGGTTTTCC

AATCCGATTGTTAAGGGGTAAACAAGATACTAGAATATATAAGCATAAAT

TACTACCGGTCAGCCAAACAACAATCCTGAAGATCAAGGTGTGACATCAT

GAAACGGCAAGTGATTATCAGCTATTTTCTCGCAGTGTGCTGCTTAGCAC

TCGTGCAGGTAATTTATCGTCAAGCATGTGCTTTGTTAGAAATTGCGAAT

ACAATTATTTTCCCGCAGGGTGAAACTGTGCAAGAT

>T18L

CGATGATGGTTTGTGCTAGTAGATTGGTTGAACATGTTTAAAGCAAAAGG

AACAATGATGCAATACAAATAATCTATAGAGCAAGCAAAACAGATTATGA

ACTTTTTTTGTGTTAAGTTGTGCGTGTCCTTAGAAATTGAGGGTCAGTGG

ATAGGAAAGATTGTGATATTTGGTTGGGAAAGAAGATCGTTAGTTATGGA

GTTGCCAGATGACGAAACTCTTTGGGTTCCACAAGATAGAACTTGAATAA

TTTCAAGATGCCTGGACTGATTCATATGTTAGCTCTCGCAAGATTAATAG

TACAACTCTCATCTTCATTACCTCAATGTCCATTTAAACTTCATTGATCA

CGTTTAACACCATATAAACAACCTATGTCGGATCATTCAAGTTAATGTGT

ATTGGGCAGTCTTGTGATACAGCTGTCAACTCGCTCGACATAACAACATA

ACCGTCGTAGGTTCAAGCCTCATATGAACCGCCCAACGCAGCAAGGATTG

AGTATCCTGCTGTGTGGCAAATAAACATAATGTAAACAAATAAACATAAT

GCAAGCATAATAATGTTGTTTTAATAACAAGTCTCGAAAGCCTAGCTAGA

CCGCATAGGTCGCTACGCAAAATAGAAAAATAAGGGTTTTTAAAAAGAAT

TTGAGGTTTTTCCTTATATTATTAAGCTAGCTGGCTTCATAAATTCTGTA

CTCAATTACTTTTTACTGTGATATGATAAACCTTCGAGTGTATAATAGAT

TCCATATAATGAAAAAGAATAAAAAATTAAGTCACAGACATTCAGAACGT

AGTGTTTTCTGATATTAGGTTTAATTCTTAAGGGTTTATAGAATATTACA

AATGAAACATCGTGTAGGATGGCGTTCAGATCGCAGCTTTCTTACACTAA

TGATTGGAACAGCTGTTTAATTTCAATATACCATATATTTTTTTATATTT

AAACAACTTATTTGCTTACCTCTGCAAATATTACACAATAAATTCAATAT

TGATACAATGCAAGTGGTTTTCTAATCCGATTGTTAAGGGGTAAACAAGA

TACTAGAATATATAAGCATAAATTACTACCGGTCAGCCAAACAACAATCC

TGAAGATCAAGGTGTGACATCATGAAACGGCAAGTGATTATCAGCTATTT

TCTCGCAGTGTGCTTGTTAGCACTCGTGCAGGTAATTTATCGTCAAGCAT

GTGCTTTGTTAGAAATTGCAAATACAATTATTTTCCCGCAGGGNGAAACT

GTGCAAGA

>T11L

ATGCAGACGAGCGATCCGTTCGATATGAACCGCGTGGCAGCGCTGATCAA

GGAAATCGATGATGGTTTGTGCTAGTAGATTGGTTGAACATGTTTAAAGC

AAAAGGAACAATGATGCAATACAAATAATCTATAGAGCAAGCAAAACAGA

TTATGAACTTTTTTTGTTTTAAGTTGTGCGTGACCTTAGAAATTGAGGGT

CAGTGGATAGGAAAGATTGTGATATTTGGGTGGGAAAGAAGATCGTTAGC

TATGGAGTTGCCAGATGACGAAACTCTTTGGGTTCCACAAGATAGAACTT

GAATAATTTCAAGATGCCTGGACTGATTCATATGTTAGCTCTCGCAAGAT

TAATAGTACAACTCTCATCTTCATTACCTCAATGTCCATTTAAACTTCAT

TGATCACGTTTAACACCATATAAACAACCTATGTCGGATCATTCAAGTTA

ATGTGTATTGGGCAGTCTTATGATACAGCTGTCAACTCGCTCGACATAAC

AACATAACCGTCGTAGGTTCAAGCCTCATATGAACCGCCCAACGCAGCAA

GGATTGAGTATCCTGCTGTGTGGCAAATAAACATAATGTAAACAAATAAA

CATAATGCAAGCATAATAATGATGTTTTAATAACAAGTCTCGAAAGCCTA

GCTAGACCGCATAGGTCGCTACGCAAAATAGAAAAATAAGGGTTAATTTG

AGGTTTTTCCTTATATTATTAAGCTAGCTGGCTTCATAAATTCTGTACTC

AATTACTTTTTACTGTGATATGATAATCCTTCGAGTGTATAATAGATTCC

ATATAATGAAAAAGAATAAAAAATTAAGTCACAGACATCCAGAACGTAGT

GTTTTCTGATATTAGGTTTAATTCTTAAGGGTTTATAGAATATTATAAAT

GAAACATCGTGTAGGATGGCGTTCAGATCGCAGCTTTCTTACACTAATGA

TTGGAACAGCTGTTTAATTTCAATATACCATATATTTTTTTATATTTAAA

CAACTTATTTGCTTACCTCTGCAAATATTACACAATAAATTCAATATTGA

TACAATGCAAGTGGTTTTCTAATCCGATTGTTAAGGGGTAAACAAGATAC

TAGAATATATAAGCATAAATTACTACCGGTCAGCCAAACAACAATCCTGA

AGATCAAGGTGTGACATCATGAAACGGCAAGTGATTATCAGCTATTTTCT

CGCAGTGTGCTTGTTAGCACTCGTGCAGGTAATTTATCGTCAAGCATGTG

CTTTGTTAGAAATTGCAAATACAATTATTTTCCCGCAGGGTGAAACTGTG

CAAGATTGTGAGAATAAGCTGCCACCGTCGCTGAAGAGTAGACTGTGCGA

GATCCG

>T1L

CGTGTGGCAGCGCTGATTAAGGAAATCGATGATGGTTTGTGCTAGTAGAT

TGGTTGAACATGTTTAAAGCAAAAGGAACAATGATGCAATACAAATAATC

TATAGAGCAAGCAAAACAGATTATGAACTTTTTTTGTGTTAAGTTGTGCG

TGACCTTAGAAATTGAGGGTCAGTGGATAGGAAAGATTGTGATATTTGGT

TGGGAAAGAAGATCGTTAGCTATGGAGTTGCCAGATTACGAAACTCTTTG

GGTTCCACAAGATAGAACTTGAATAATTTCAAGATGCCTGGACTGATTCA

TATGTTAGCTCTCGCAAGATTAATAGTACAACTCTCATCTTCATTACCTC

AATGTCCATTTAAACTTCATTGATCACGTATAACACCATATAAACAACCT

ATGTCGGATCATTCAAGTTAATGTGTATTGGGCAGTCTTGTGATACAGCT

GTCAACTCGCTCGACATAACAACATAACCGTCGTAGGTTCAAGCCTCATA

TGAACCGCCCAACGCAGCAAGGATTGAGTATCCTGCTGTGTGGCAAATAA

ACATAATGTAAACAAATAAACATAATGCAAGCATAATAATGATGTTTTAA

TAACAAGTCTCGAAAGCCTAGCTAGACCGCATAGGTCGCTACGCAAAATA

GAAAAATAAGGGTTTTTAAAAAGGATTTGAGGTTTTTCCTTATATTATTA

AGCTAGCTGGCTTCATAAATTCTGTACTCAATTACTTTTTACTGTGATAT

GATAAACCTTCGAGTGTATAATAGATTCCATATAATGAAAAAGAATAAAA

AATTAAGTCACAGACATCCAGGACGTAGTGTTTTCTGATATTAGGTTTAA

TTCTTAAGGGTTTATAGAATATTACAAATGAAACATCGTGTAGGATGGCG

TTCAGAACGCAGCTTTCTTACACTAATGATTGGAACAGCTGTTTAATTTC

AATATACCATATATTTTTTTATATTTAAAGAACACATTTGCTTACCTCTG

CAAATATTACACAATAAATTCAATATTGATACAAGGCAAGTGGTTTTCTA

ATCCGATTGTTAAGGGGTAAACAAGATACTAGAATATATAAGCATAAATT

ACTACCGGTCAGCCAAACAACAATCCTGAAGATCAAGGTGTGACATCATG

AAACGGCAAGTGATTATCAGCTATTTTCTCGCAGTGTGCTGCTTAGCACT

CGTGCAGGTAATTTATCGTCAAGCATGTGCTTTGTTAGAAATTGCAAATA

CAATTATTTTCCCGCAGGG

>T12S

ACGTGTGGCTGCACTGATCAAGGAAATCGATGATGGTTTGTGCTAGTAGA

TTGATTGAACATGTTTAAAGCAAAAGGAACAATGATGCAATACAAATAAT

CTATAGAGCAAGCAAAACAGATTATGAACTTTTTTTGTGTTAAGTTGTGC

GTGACCTTAGAAATTGAGGGTCAGTGGATAGGAAAGATTGTGATATTTGG

GTGGGAAAGAAGATCGTTAGCTATGGAGTTGCCAGATGACGAAACTCTTT

GGGTTCCACAAGATAGAACTTGAATAATTTCAAGATGCCTGGACTGATTC

ATATGTTAGCTCTCGCAAGATTAATAGTACAACTCTCATCTTCATTACCT

CAATGTCCATTTAAACTTCATTGATCACGTTTAACACCATATAAACAACC

TATGTCGGATCATTCAAGTTAATGTGTATTGGGCAGTCTTGTGATACAGC

TGTCAACTCGCTCGACATACCAACATAACCGTCGTAGGTTCAAGCCTCAT

ATGTACCGCCCAACGCAGCAAAGATTGAGTATCCTGCTGTGTGGCAAATA

AACATAATGTAAACAAATAAACATAATGCAAGCATAATAATGATGTTTTA

ATAACAAGTCTCGAAAGCCTAGCTAGACCGCATAGGTCGCTACGCAAAAT

AGAAAAATAAGGGTTTTTAAAAAGGATTTGAGGTTTTTCCTTATATTATT

AAGCTAGCTGGCTTCATAAATTCTGTACTCAATTACTTTTTACTGTGATA

TGATAAACCTTCGAGTGTATAATAGATTCCATATAATGAAAAAGAATAAA

AAATTAAGTCACAGACATCCAGAACGTAGTGTTTTCTGATATTAGGTTTA

ATTCTTAAGGGTTTATAGAATATTACAAATGAAACATCGTGTAGGATAGC

GTTCAGCTAGCAGCTTTCTAACACTAATGATTGGAACAGCTGTTTAATTT

CAATATACCATATATTTTTTTATATTTAAACAACACATTTGCTTACCTCT

GCAAATATTACTCAATAAATTCAATATTGATACAATGCAAGTGGTTTTCT

AATCCGATTGTTAAGGGGTAAACAAGATACTAGAATATATAAGCATAAAT

TACTACCGGTCAGCCAAACAACAATCCTGAAGATCAAGGTGTGACATCAT

GAAACGGCAAGTGATTATCAGCTATTTTCTCGCAGTGTGCTGCTTAGCAC

TCGTGCAGGTAATTTATCGTCAAGCATGTGCTTTGTTAGAAATTGCAAAT

ACAATTATTTTCCCGCAGAGNGAAACTGTACAAG

>T14L

ACGTGTGGCAGCGCTGATTAAGGAAATCGATGATGGTTTGTGCTAGTAGA

TTGGTTGAACATGTTTAAAGCAAAAGGAACAATGATGCAATACAAATAAT

CTATAGAGCAAGCAAAACAGATTATGAACTTTTTTTGTGTTAAGTTGTGC

GTGACCTTAGAAATTGAGGGTCAGTGGATAGGAAAGATTGTGATATTTGG

TTGGGAAAGAAGATCGTTAGCTATGGAGTTGCCAGATGACGAAACTCTTT

GGGTTCCACAAGATAGAACTTGAATAATTTCAAGATGCCTGGACTGATTC

ATATGTTAGCTCTCGCAAGATTAATAGTACAACTCTCATCTTCATTACCT

CAATGTCCATTTAAACTTCATTGATCACGTTTAACACCATATAAACAACC

TATGTCGGATCATTCAAGTTAATGTGTATTGGGCAGTCTTGTGATACAGC

TGTCAACTCGCTCGACATAACAACATAACCGTCGTAGGTTCAAGCCTCAT

ATGAACCGCCCAACGCAGCAAGGATTGAGTATCCTGCTGTGTGGCAAATA

AACATAATGTAAACAAATAAACATAATGCAAGCATAATAATGATGTTTTA

ATAACAAGTCTCGAAAGCCTAGCTAGACCGCATAGGTCGCTACGCAAAAT

AGAAAAATAAGGGTTTTTAAAAAGGATTTGAGGTTTTTCCTTATATTATT

AAGCTAGCTGGCTTCATAAATTCTGTACTCAATTACTTTTTACTGTGATA

TGATAAACCTTCGAGTGTATAATAGATTCCATATAATGAAAAAGAATAAA

AAATTAAGTCACAGACATCCAGAACGTAGTGTTTTCTGATATTAGGTTTA

ATTCTTAAGGGTTTATAGAATATTACAAATGAAACATCGTGTAGGATGGC

GTTCAGATCGCAGCTTTCTTACACTAATGATTGGAACAGCTGTTTAATTT

CAATATACCATATATTTTTTTTTATATTTAAACAACTTATTTGCTTACCT

CTGCAAATATTACACAATAAATTCAATATTGATACAATGCAAGTGGTTTT

CTAATCCGATTGTTAAGGGGTAAACAAGATACTAGAATATATAAGCATAA

ATTACTACCGGTCAGCCAAACAACAATCCTGAAGATCAAGGTGTGACATC

ATGATACGGCAAGTGATTATCAGCTATTTTCTCACAGTGTGCTTGTTAGC

ACTCGTTCAGGTAATGGATCATGAAGTAAGTCTTTATTAGAAATTACAAA

TACAATTATTTTCATGCAGGGTGAGAC

>T17

GTGTGGCAGCGCTGATTAAGGAAATCGATGATGGTTTGTGCTAGTAATTG

GTTGAACATGTTTAAAGCAAAAGGAACAATGATGCAATACAAATAAAATA

TAAAGCAAGCAAAACAAATTATGGACTTTTTGTGTGTTAAGTTGCGCGTG

ACTTTGGGAAATGAGGGTCAGTGGATAAGTAAGATTATGATATTTGGTTG

GGAAAGAAGATCGTTAGCTATGGAGTTGCCAGATGACAAAACTCTTTGGG

TTCCACAAGATAGAACTTGAATAATTTCAAGATGCCTGGACTGATTCATA

TGTTAGCTCTCGCAAGATTAATAGTACAACTCTCATCTTCATTACCTCAA

TGTCCATTTAAACTTCATTGATCACGTTTAACACCATATAAACAACCTAT

GTCGGATCATTCAAGTTAATGTGTATTGGGCAGTCTTGTGATACAGCTGT

CAACTCGCTCGACATAACAACATAACCGTCGTAGGTTCAAGCCTCATATG

AACCGCCCAACGCAGCAAGGATTGAGTATCCTGCTGTGTGGCAAATAAAC

ATAATGTAAACAAATAAACATAATGCAAGCATAATAATGATGTTTTAATA

ACAAGTCTCGAAAGCCTAGCTAGACCGCATAGGTCGCTACGCAAAACAGA

AAAATAAGGGTTTTTAAAAAGGATTTGAGGTTTTTCCTTATATTATTAAG

CTAGCTGGCTTCATAAATTCTGTACTCAATTACTTTTTACTGTGATATGA

TAAACCTTCGAGTGTATAATAGATTCCATATAATGAAAAAGAATAAAAAA

TTAAGTCACAGACATTCAGAACGTAGTGTTTTCTGATATTAGGTTTAATT

CTTAAGGGTTTATAGAATATTACAAATGAAACATCGTGTAGGATGGCGTT

CAGATCGCAGCTTTCTTACACTAATGATTGGAACAGCTGTTTAATTTCAA

TATACCATATATTTTTTTATATTTAAACAACTTATTTGCTTACCTCTGCA

AATATTACACAATAAATTCAATATTGATACAATGCAAGTGGTTTTCTAAT

CCGATTGTTAAGGGGTAAACAAGATACTAGAATATATAAGCATAAATTAC

TACCGGTCAGCCAAACAACAATCCTGAAGATCAAGGTGTGACATCATGAA

ACGGCAAGTGATTATCAGCTATTTTCTTGCAGTGTGCTTGTTAGCACTCG

TGCAGGTAATTTATCGTCAAGCATGTGCTTTGTTAGAAATTGCAAATACA

ATTA

>T10S

GACGTGTGGCAGCGCTGATCAAGGAAATCGATGATGGTTTGTGCTAGTAG

ATTGGTTTAAAAACATGTTTAAAGCAAAAGGAACAATGATGCAATACAAA

TAATCTATAGAGCAAGCAAAACAGATTATGGACTTTTTGTGTGTTAAGCT

GCGCGTGGCTTTGGGAAATGAGGGTCAGTGGATAAGAAGAATTATGATAT

TTGGATTCTTCAAAGAAGATCGTTTACTATGGAGCTGCCACATGACGAAT

CTCGTTGGGTTCCACACCTGAAACATTTCATGATGCCTGAACTGATTCAT

ATATTTGCTCTCGCATAATTAATAGTACAACTCTCATCTTTATTACCTAA

ATGTCCATTTATCGGTCTAACTTCATCGATCGCGTTTAACACCATATAAA

CAACCTATGTCGGATGATGCAAGTTAATGTGTATTGGGCAGTCTTGTGGT

ACAGCTCGTGGTCAACTCGCTCAACTAAACAACATGCCCGTCGTGGGTTC

AAGCCTCATATGGACCGCTCTACTTAGCAAGGAGTGAGTATTTAGCTGTG

TGATGATTAACAATAGGTATCGAACGCCTACATAGACCGGTTAAAAAAAG

GTTTTTTAAAGGGTTTGAGGTTTTTCCTTATATTATTAAACTAGCTGGCT

TTATACATTCTGTACTCAACTAAATTTTTCTGTGATATGATAAACCTTCA

AATGTATAATAGATTCCATATAATGAAAAAGAATAAAAATTTAAGCCACA

GACATCCAGGAAACAGTGTTTTCTGATGTTAGTTTTAATTCGTAAGGGTT

TATAGAATATTACAAATAAAACATCGTGTCGGATGGCGTTCAGATAGCAG

CTTTCTCACACGAATGATTAGAACAGCTGTTAAACTTCAATATACCATGT

ATGTTTTTATATTTAAACAACACATTTGCTTACCTCTGTAAATATTACTC

GATAATTGCAATATTGATACAAGGCAAGTGGTTTTCCAATCCGATTGTCA

ATGGGTAAACAAGATACTAGAATATATAAGCATAAATTACTACCGATCAG

CCAAACAACAATCCTGAAGATCAAGGTGTGACATCATGAAACGGCAAGTG

ATTATCAGCTATTTTCTCGCAGTGTGCTGCTTAGCACTCGTGCAGGTAAT

TTATCGTCAAGCATGTGCTTTGTTAGAAATTGCAAATACAATTATTTTCC

CGCAGAGTGAAAC

>T18S

GTGGCAGCGCTGATCAAGGAAATCGATGATGGTTTGTGCTAGTAGATTGG

TTTAAAAACATGTTTAAAGCAAAAGGAACAATGATGCAATACAAATAATC

TATAGAGCAAGCAAAACAGATTATGGACTTTTTGTGTGTTAAGCTGCGCG

TGGCTTTGGGAAATGAGGGTCAGTGGATAAGAAGAATTATGATATTTGGA

TTCTTCAAAGAAGATCGTTTACTATGGAGCTGCCACATGACGAATCTCGT

TGGGTTCCACACCTGAAACATTTCATGATGCCTGAACTGATTCATATATT

TGCTCTCGCATAATTAATAGTACAACTCTCATCTTTATTACCTAAATGTC

CATTTATCGGTCTAACTTCATCGATCGCGTTTAACACCATATAAACAACC

TATGTCGGATGATGCAAGTTAATGTGTATTGGGCAGTCTTGTGGTACAGC

TCGTGGTCAACTCGCTCAACTAAACAACATGCCCGTCGTGGGTTCAAGCC

TCATATGGACCGCTCTACTTAGCAAGGAGTGAGTATTTAGCTGTGTGATG

ATTAACAATAGGTATCGAACGCCTACATAGACCGGTTAAAAAAAGGTTTT

TTAAAGGGTTTGAGGTTTTTCCTTATATTATTAAACTAGCTGGCTTCATA

CATTCTGTACTCAACTAAATTTTTCTGTGATATGATAAACCTTCAAATGT

ATAATAGATTCCATATAATGAAAAAGAATAAAAATTTAAGCCACAGACAT

CCAGGAAACAGTGTTTTCTGATGTTAGTTTTAATTCGTAAGGGTTTATAG

AATATTACAAATAAAACATCGTGTAGGATGGCGTTCAGATAGCAGCTTTC

TCACACGAATGATTAGAACAGCTGTTAAACTTCAATATACCATGTATGTT

TTTATATTTAAACAACACATTTGCTTACCTCTGTAAATATTACTCGATAA

TTGCAATATTGATACAAGGCAAGTGGTTTTCCAATCCGATTGTCAATGGG

TAAACAAGATACTAGAATATATAAGCATAAATTACTACCGGTCAGCCAAA

CAACAATCCTGAAGATCAAGGTGTGACATCATGAAACGGCAAGTGATTAT

CAGCTATTTTCTCGCAGTGTGCTGCTTAGCACTCGTGCAGGTAATTTATC

GTCAAGCATGTGCTTTGTTAGAAATTGCGAATACAATTA

>T16L

TGTGGCAGCGCTGATTAAGGAAATCGATGATGGTTTGTGCTAGTAGATTG

GTTGAACATGTTTAAAGCAAAAGGAACAATGATGCAATACAAATAATCTA

TAGAGCAAGCAAAACAGATCATGGACTTTTTGTGTGTTAAGCTGCGCGTG

GCTTTGGGAAATGAGGGTCAGTGGATAAAAAGAATTATGATATTTGGATT

CTTCAAAGAAGATCGTTTACTATGGAGCTGCCACAAGACGAATCTCGTTG

GGTTCCACACCAGAAACATTTCATGATGCCTGGACTGATTCATATATTTG

CTCTCGCATAATTAATAGTACAACTCTCATCTTTATTACCTAAATGTCCA

TTTATCGGTCTAACTTAATCGATCGCGTTTAACACCATATAAACAACCTA

TGTCGGATGATGCAAGTTAATGTGTATTGGGCAGTCTTGTGGTACAGCTC

GTGGTCAACTCGCTCAACTAAACAACATACCCGTCGTGGGTTCAAGCCTC

TATATGGACCGCTCTACTTAGCAAAGAGTGAGTATTTAGCTGTGTGATGA

TTAACAATAGGTATCGAACGCCTACATAGACCGGTTAAAAAAAGGTTTTT

TAAAGGGTTTGAGGTTTTTCCTTATATTATTAAACTAGCTGGCTTCATAC

ATTCTGTACTCAACTAAATTTTTCTGTGATATGATAAACCTTCAAATGTA

TAATAGATTCCATATAATTAAAAAGAATAAAAAATTAAGCCACAGACATC

CAGGAAACAGTGTTTTCTGATGTTAGTTTTAATTCATAAGGGTTTATAGA

ATATTACAAATAAAACATCGTGTAGGATGGCGTTCAGATAGCAGCTTTCT

CACACGAATGATTAGAACAGCTGTTAAACTTCAATATACCATGTATGTTT

TTATATTTAAACAACACATTTGCTTACCTCTGTAAATATTACTCGATAAT

TGCAATATTGATACAAGGCAAGTGGTTTTCCAATCCGATTGTCAATGGGT

AAACAAGATACTAGAATATATAAGCATAAATTACTACCGATCAGCCAAAC

AACAATCCTGAAGATCAAGGTGTGACATCATGAAACGGCAAGTGATTATC

AGCTATTTTCTCGCAGTGTGCTGCTTAGCACTCGTGCAGGTAATTTATCG

TCAAGCATGTGCTTTGTTAGAAATTGCAAATACAATTATTTCCCGCAGGG

NGAAACTGTGCAAGA

>TC50L

GCTACTGAAGGCTGGCAAGATGCAGACGAGCGATCCGTTCGATATGAACC

GTGTGGCAGCGCTGATTAAGGAAATCGATGATGGTTTGTGCTAGTAGATT

GGTTGAACATGTTTAAAGCAAAAGGAACAATGATGCAATACAAATAATCT

ATAGAGCAAGCAAAACAGATCATGGACTTTTTGTGTGTTAAGCTGCGCGT

GGCTTTGGGAAATGAGGGTCAGTGGATAAAAAGAATTATGATATTTGGAT

TCTTCAAAGAAGATCGTTTACTATGGAGCTGCCACAAGACGAATCTCGTT

GGGTTCCACACCAGAAACATTTCATGATGCCTGGACTGATTCATATATTT

GCTCTCGCATAATTAATAGTACAACTCTCATCTTTATTACCTAAATGTCC

ATTTATCGGTCTAACTTAATCGATCGCGTTTAACACCATATAAACAACCT

ATGTCGGATGATGCAAGTTAATGTGTATTGGGCAGTCTTGTGGTACAGCT

CGTGGTCAACTCGCTCAACTAAACAACATGCCCGTCGTGGGTTCAAGCCT

CTATATGGACCGCTCTACTTAGCTAGGAGTGAGTATTTAGCTGTGTGATG

ATTAACAATAGGTATCGAACGCCTACATAGACCGGTTAAAAAAAGGTTTT

TTAAAGGGTTTGAGGTTTTTCCTTATATTATTAAACTAGCTGGCTTCATA

CATTCTGTACTCAACTAAATTTTTCTGTGATATGATAAACCTTCAAATGT

ATAATAGATTCCATATAATTAAAAAGAATAAAAAATTAAGCCACAGACAT

CCAGGAAACAGTGTTTTCTGATGTTAGTTTTAATTCATAAGGGTTTATAG

AATATTACAAATAAAACATCGTGTAGGATGGCGTTCAGATAGCAGCTTTC

TCACACGAATGATTAGAACAGCTGTTAAACTTCAATATACCATGTATGTT

TTTATATTTAAACAACACATTTGCTTACCTCTGTAAATATTACTCGATAA

TTGCAATATTGATACAAGGCAAGTGGTTTTCCAATCCGATTGTCAATGGG

TAAACAAGATACTAGAATATATAAGCATAAATTACTACCGATCAGCCAAA

CAACAATCCTGAAGATCAAGGTGTGACATCATGAAACGGCAAGTGATTAT

CAGCTATTTTCTCGCAGTGTGCTGCTTAGCACTCGTGCAGGTAATTTATC

GTCAAGCATGTGCTTTGTTAGAAATTGCAAATACAATTATTTTCCCGCAG

GGTGAAACTGTGCAAGATTGTGAGAATAAGCTGCCACCGTCGCTGAAGAG

TAGACTGTGCGAGATCCG

>TC55

GTGGCAGCGCTGATCAAGGAAATCGATGATGGTTTGTGCTAGTAGATTGG

TTGAACATGTTTAAAGCAAAAGGAACAATGATGCAATACAAATAATCTAT

AGAGCAAGCAAAACAGATCATGGACTTTTTGTGTGTTAAGCTGCGCGTGG

CTTTGGGAAATGAGGGTCAGTGGATAAAAAGAATTATGATATTTGGATTC

TTCAAAGAAGATCGTTTACTATGGAGCTGCCACAAGACGAATCTCGTTGG

GTTCCACACCAGAAACATTTCATGATGCCTGGACTGATTCATATATTTGC

TCTCGCATAATTAATAGTACAACTCTCATCTTTATTACCTAAATGTCCAT

TTATCGGTCTAACTTAATCGATCGCGTTTAACACCATATAAACAACCTAT

GTCGGATGATGCAAGTTAATGTGTATTGGGCAGTCTTGTGGTACAGCTCG

TGGTCAACTCGCTCAACTAAACAACATGCCCGTCGTGGGTTCAAGCCTCT

ATATGGACCGCTCTACTTAGCAAGGAGTGAGTATTTAGCTGTGTGATGAT

TAACAATAGGTATCGAACGCCTACATAGACCGGTTAAAAAAAGGTTTTTT

AAAGGGTTTGAGGTTTTTCCTTATATTATTAAACTAGCTGGCTTCATACA

TTCTGTACTCAACTAAATTTTTCTGTGATATGATAAACCTTCAAATGTAT

AATAGATTCCATATAATTAAAAAGAATAAAAAATTAAGCCACAGACATCC

AGGAAACAGTGTTTTCTGATGTTAGTTTTAATTCATAAGGGTTTATAGAA

TATTACAAATAAAACATCGTGTAGGATGGCGTTCAGATAGCAGCTTTCTC

ACACGAATGATTAGAACAGCTGTTAAACTTCAATATACCATGTATGTTTT

TATATTTAAACAACACATTTGCTTACCTCTGTAAATATTACTCGATAATT

GCAATATTGATACAAGGCAAGTGGTTTTCCAATCCGATTGTCAATGGGTA

AACAAGATACTAGAATATATAAGCATAAATTACTACCGATCAGCCAACCA

ACAATCCTGAAGATCAAGGTGTGACATCATGAAACGGCAAGTGATTATCA

GCTATTTTCTCGCAGTGTGCTGCTTAGCACTCGTGCAGGTAATTTATCGT

CAAGCATGTGCTTTGTTAGAAATTGCAAATACAATTATTTTCCCGCAGGG

NGAAACTGTGCAAGAT

>T1S

CGTGTGGCAGCGCTGATCAAGGAAATCGATGATGGTTTGTGCTAGTAGAT

TGGTTGAACATGTTTAAAGCAAAAGGAACAATGATGCAATACAAATAATC

TATAGAGCAAGCAAAACAGATCATGGACTTTTTGTGTGTTAAGCTGCGCG

TGGCTTTGGGAAATGAGGGTCAGTGGATAAAAAGAATTATGATATTTGGA

TTCTTCAAAGAAGATCGTTTACTATGGAGCTGCCACAAGACGAATCTCGT

TGGGTTCCACACCAGAAACATTTCATGATGCCTGGACTGATTCATATATT

TGCTCTCGCATAATTAATAGTACAACTCTCATCTTTATTACCTAAATGTC

CATTTATCGGTCTAACTTAATCGATCGCGTTTAACACCATATAAACAACC

TATGTCGGATGATGCAAGTTAATGTGTATTGGGCAGTCTTGTGGTACAGC

TCGTGGTCAACTCGCTCAACTAAACAACATGCCCGTCGTGGGTTCAAGCC

TCTATATGGACCGCTCTACTTAGCAAAGAGTGAGTATTTTGCTGTGTGAT

GATTAACAATAGGTATCGAACGCCTACATAGACCGGTTAAAAAAGGTTTT

TTAAAGGGTTTGAGGTTTTTCCTTATATTATTAAACTAGCTGGCATCATA

CATTCTGTACTCAACTAAATTTTTCTGTGATATGATAAACCTTCAAATGT

ATAATAGATTCCATATAATTAAAAAGAATAAAAATTTAAGCCACAGACAT

CCAGGAAACAGTGTTTTCTGATGTTAGTTTTAATTCGTAAGGGTTTATAG

AATATTACAAATAAAACATCGTGTAGGATGGCGTTCAGATAGCAGCTTTC

TCACACGAATGATTAGAACAGCTGTTAAACTTCAATATACCATGTATGTT

TTTATATTTAAACAACACATTTGCTTACCTCTGTAAATATTACTCGATAA

TTGCAATATTGATACAAGACAAGTGGTTTTCCAATCCGATTGTCAATGGG

TAAACAAGATACTAGAATATATAAGCATAAATTACTACCGATCAGCCAAA

CAACAATCCTGAAGATCAAGGTGTGACATCATGAAACGGCAAGTGATTAT

CAGCTATTTTCTCGCAGTGTGCTTGTTAGCACTCGTGCAGGTAATTTATC

GTCAAGCATGTGCTTTGTTAGAAATTGCCAATACAATTATTTTCCCGCAG

GGTGAAACTGTGCAAGA

>TC8L

CGTGTGGCAGCGCTGATTAAGGAAATCGATGATGGTTTGTGCTAGTAGAT

TGGTTGAACATGTTTAAAGCAAAAGGAACAATGATGCAATACAAATAATC

TATAGAGCAAGCAAAACAGATCATGGACTTTTTGTGTGTTAAGCTGCGCG

TGGCTTTGGGAAATGAGGGTCAGTGGATAAAAAGAATTATGATATTTGGA

TTCTTCAAAGAAGATCGTTTACTATGGAGCTGCCACAAGACGAATCTCGT

TGGGTTCCACACCAGAAACATTTCATGATGCCTGGACTGATTCATATATT

TGCTCTCGCATAATTAATAGTACAACTCTCATCTTTATTACCTAAATGTC

CATTTATCGGTCTAACTTAATCGATCGCGTTTAACACCATATAAACAACC

TATGTCGGATGATGCAAGTTAATGTGTATTGGGCAGTCTTGTGGTACAGC

TCGTGGTCAACTCTCTCAACTAAACAACATGCCCGTCGTGGGTTCAAGCC

TCTATATGGACCGCTCTACTTAGCAAGGAGTGAGTATTTAGCTGTGTGAT

GATTAACAATAGGTATCGAACGCCTACATAGACCGGTTAAAAAAAGGTTT

TTTAAAGGGTTTGAGGTTTTTCCTTATATTATTAAACTAGCTGGCTTCAT

ACATTCTGTACTCAACTAAATTTTTCTGTGATATGATAAACCTTCAAATG

TATAATAGATTCCATATAATTAAAAAGAATAAAAAATTAAGCCACAGACA

TCCAGAACGTAGTGTTTTCTGATATTAGGTTTAATTCTTAAGGGTTTATA

GAATATTACAAATGAAACATCGTGTAGGATAGCGTTCAGCTAGCAGCTTT

CTAACACTAATGATTGAAACAGCTGTTTAATTTCAATATACCATATATTT

TTTTATATTTAAAGAACACATTTGCTTACCTCTGCAAATATTACACAATA

AATTCAATATTGATACAAGGCAAGTGGTTTTCTAATCCGATTGTTAAGGG

GTAAACAAGATACTAGAATATATAAGCATAAATTACTACCGGTCAGCCAA

ACAACAATCCTGAAGATCAAGGTGTGACATCATGAAACGGCAAGTGATTA

TCAGCTATTTTCTCGCAGTGTGCTGCTTAGCACTCGTGCAGGTAATTTAT

CGTCAAGCATGTGCTTTGTTAGAAATTGCAAATACAATTATTTTCCCGCA

GGGTGAAACTGTGCAAGA

>T14S

ACGTGTGGCAGCGCTGATCAAGGAAATCGATGATGGTTTGTGCTAGTAGA

TTGGTTGAACATGTTTAAAGCAAAAGGAACAATGATGCAATACAAATAAT

CTATAGAGCAAGCAAAACAGATTATGGACTTTTTGTGTGTTAAGCTGCGC

GTGGCTTTGGGAAATGAGGGTCAGTGGATAAGAAGAATTATGATATTTGG

ATTCTTCAAAGAAGATCGTTTACTATGGAGCTGCCACATGACGAATCTCG

TTGGGTTCCACACCTGAAACATTTCATGATGCCTGGACTGATTCATATAT

TTGCTCTCGCATAATTAATAGTACAACTCTCATCTTTATTACCTAAATGT

CCATTTATCGATCGAACTTCATCGATCGCGTTTAACACCATATAAACAAC

CTATGTCGGATGATGCAAGTTAATGTGTATTGGGCAGTCTTGTGGTACAG

CTCGTGGTCAACTCGCTCAACTAAACAACATGCCCGTCGTGGGTTCAAGC

CTCATATGGACCGCTCTACTTAGCAAGGAGTGAGTATTTAGCTGTGTGAT

GATTAACAATAGGTATCGAACGCCTGCATAGACCGGTTAAAAAAAGGTTT

TTTAAAGGGTTTGAGGTTTTTCCTTATATTATTAAACTAGCTGGCTTCAT

ACATTCTGTACTCAACTAAATTTTTCTGTGATATGATAAACCTTCAAATG

TATAATAGATTCCATATAATGAAAAAGAATAAAAATTTAAAATTTAAAAA

GAATAAATATTACAAATAAAACATCGTGTAGGATGGCGTTCAGATGGCAG

CTTTCTCACACGAATGATTAGAACAGCTGTTAAACTTCAATATACCATGT

ATGTTTTTATATTTAAACAACACATTTGCTTACCTCTGTAAATATTACTC

GATAATTGCAATATTGATACAAGGCAAGTGGCTTTCCAATCCGATTGTCA

ATGGGTAAACAAGATACTAGAATATATAAGCATAAATTACTACCGATCAG

CCAAACAACAATCCTGAAGATCAAGGTGTGACATCATGATACGGCAAGTG

ATTATCAGCTATTTTCTCACAGTGTGCTTGTTAGCACTCGTTCAGGTAAT

GGATCAGGAAGTAAGTCCTTATTAGAAATTACAAATTACAATTATTTTCA

AGCAGGGTGAGACTGTGCAAGA

>T7M

GCTACTGAAGGCTGGCAAGATGCAGACGAGCGATCCGTTCGATATGAACC

GTGTGGCAGCGCTGATCAAGGAAATCGATGATGGTTTGTGCTAATAGTAG

ATTGGTTGAACATATAGCAAAAGCAAAATAAGCAAAAGGAACAATGATGC

AATACAAATAATCTATAGAGCAAGCAAAACAGATTATGAACTTTTTGTGT

GTTAAGTTGTGCGTGACCTTAGAAAATGAGGGTCAGTGGATACGAAAGAT

TGTGATATTTGGGTTCTTGAAAGAAGATCGTTAGCTATGGAGTTGCCAGA

TGACGTAACTTGTTGGATTCCACAAAATAGAACTTGAAACATGTCAAGAT

GCCTGGACTGATTCATATGTTTATACATGTAAGATTAATAGTACAACTAT

CATCTTCATTACCTTTAAGTCCTTTTAAAATTCATCAATCCCGTTTAACA

CCATATAAACAACCTAGAAAAAAAAAAATAGAAAAATAAGGGTTTTTAAA

AAGGATTTGAGGTTTTTCCTTATATTATTAAGCTAGCTGGCTTCATAAAT

TCTGTACTCAATTACTTTTTACTGTGATATGATAAACCTTCGAATGTATA

ATAGATTCCATATAATGAAAAAGAATAAAAAATTAAGTCACAGACATCCA

GAACGTAGTGTTTTCTGATATTAGGTTTAATTCTTAAGGGTTTATAGAAT

ATTACAAATGAAACATCGTGTAGGATGGCGTTCAGCTAGCAGCTTTCTTA

CACTAATGATTGGAACAGCTGTTTAATTTCAATATACCATATATTTTTTT

TATATTTAAACAACACATTTGCTTACCTCTGCAAATATTACACAATAAAT

TCAATATTGATACAATGCAAGTGGTTTTCCAATCCGATTGTTAATGGTTA

AACAAGATACTAGAATATATAAGCATAAATTACTACCGATCAGCCAAACA

ACAATCCTGAAGATCAAGGTGTGACATCATGAAACGGCAAGTGATTATCA

GCTATTTTCTCGCAGTGTGCTGCTTAGCACTCGTGCAGGTAATTTATCGT

CAAGCATGTGCTTTGTTAGAAATTGCAAATACAATTATTTTCATGCAGGG

TGAGACTGTGCAAGATTGTGAGAATAAGCTGCCACCGTCGCTGAAGAGTA

GACTGTGCGAGATCCG

>TC50S

CGCTGATCAAGGAAATCGATGATGGTTTGTGCTAATAGTAGATTGGTTGA

ACATATAGCAAAAGCAAAATAAGCAAAAGGAACAATGATGCAATACAAAT

AATCTATAGAGCAAGCAAAACAGATTATGAACTTTTTGTGTGTTAAGTTG

TGCGTGACCTTAGAAAATGAGGGTCAGTGGATACGAAAGATTGTGATATT

TGGGTTCTTGAAAGAAGATCGTTAGCTATGGAGTTGCCAGATGACGTAAC

TTGTTGGATTCCACAAAATAGAACTTGAAACATGTCAAGATGCCTGGACT

GATTCATATGTTTATACATGTAAGATTAATAGTACAACTATCATCTTCAT

TACCTTTAAGTCCTTTTAAAATTCATCAATCCCGTTTAACACCATATAAA

CAACCTAGAAAAAAAAAAATAGAAAAATAAGGGTTTTTAAAAAGGATTTG

AGGTTTTTCCTTATATTATTAAGCTAGCTGGCTTCATAAATTCTGTACTC

AATTACTTTTTACTGTGATATGATAAACCTTCGAATGTATAATAGATTCC

ATGTAATGAAAAAGAATAAAAAATTAAGTCACAGACATCCAGAACGTAGT

GTTTTCTGATATTAGGTTTAATTCTTAAGGGTTTATAGAATATTACAAAT

GAAACATCGTGTAGGATGGCGTTCAGCTAGCAGCTTTCTTACACTAATGA

TTGGAACAGCTGTTTAATTTCAATATACCATATATTTTTTTTATATTTAA

ACAACACATTTGCTTACCTCTGCAAATATTACACAATAAATTCAATATTG

ATACAATGCAAGTGGTTTTCCAATCCGATTGTTAATGGTTAAACAAGATA

CTAGAATATATAAGCATAAATTACTACCGATCAGCCAAACAACAATCCTG

AAGATCAAGGTGTGACATCATGAAACGGCAAGTGATTATCAGCTATTTTC

TCGCAGTGTGCTGCTTAGCACTCGTGCAGGTAATTTATCGTCAAGCATGT

GCTTTGTTAGAAATTGCAAATACAATTATTTTCATGCAGGGTGAGACTGT

GCAAGATG

>T4L

ATGCTACTGAAGGCTGGCAAGATGCAGACGAGCGATCCGTTCGATATGAA

CCGTGTGGCTGCGCTGATCAAGGAAATCGATGATGGTTTGTGCTAGTAGA

TTGATTGAACATGTTTAAAGCAAAAGGAACAATGATGCAATACAAATAAT

CTATAGAGCAAGCAAAACAGATTATGGACTTTTTGTGTGTAAAGTTGTGC

GTGACTTTGGGAAATGAGGGTCAGTGGATGAGAAAGATTGTGAAATTTGG

GTTCTTGAAAGAAGATCGTTAGCTATGGAGTATCCAGATGACGAAATTCG

TTGGGTGCTACAAGATAGAACTCGAATCATTTCAAGATGCCTGGACTGAT

TCATATGTTTGCTCTTGCAAGATTAATGATGATGATGATGATAAGTCCCA

CCTCTTACCTCAACACAGGTTTGAGAAGGACGAAAGTATCTATAAGTTAA

TTCTACTCTAATAGTTGTTATGAAATAAAAACGAGCAAGTGGTGGCTTCG

GAAAACTCTCAGAGGAACCGTCCGAGTCATACACACACCCAAGATGACCA

ACATAGTTTCATCAAGAAAAAACGGGTCATACTCCATCGAATACAATAGT

ATTCCCTAGCATCCTTTACGTAGTCCGCCAAGAACCAATTCATGGATCCG

GCGCACCACTTATCAGGACACAACTGCGACATGCATATGGCCAGTTCCAC

TAGTGCCAACCGCTGACTCCAGGGTCTCTGGCACCAACGGCTGGCTCCAG

GGCACTACCTTCCTAAGCAGGAGAATTTAATGTATCCCTTACGTAGCTCG

CCAAGAACCAACTAGTGGATCCGGCGCACTACTTATCGGAACACAACTGC

AACATGCATATGGCCAGTTCCACTAGTGCCAATGGTTGGCTCTAGGGCAC

TACCTTCCGAAGCAGGTGAACCTAATGTATCTGGAATTGGCCACTTACAA

TCATCAATGAGTGAATCCTGTTGTATTAATAGTACACTCTCATCTTCATT

ACCNTTAATGTCCATTTAAACTTCATCGATCGCGTTTAACACCATAGAAA

CAACCGATGACAGATTATTCAAGTTAATGTGTATTGGGAGGTCTTGTGGT

ACAGCTGTCAACTCGCTCGACTTAACAACATGCCCGTCGTGGGTTCAAGC

CTCATATGGACCGCTCTATGTAGCAAGGAGTGAGTATCCTGTTGTGTGGC

ACATAAATATAATGATGCTGTGTTAATAATAAGTCTCGAAAGCCTAGCTA

GACTGCATAAGTCGCAACGCCAAATAGAAAAATAAGGTTTTTTTAAAGGA

TTCGAGATGTTTCTTTATATTTAAGCAAGCTGGCTTAATAAATTTTGTTT

ACATCAACTACTATTTACTGTGATAAGATAAACCTTCGAGTGTATAATAT

ATTCGATATAATGAAAACAGAATAAAAATTTAAGTCACAGACATCCAGAA

AGCAGTGTTTTCTGATGTTTGTTTTAATTCGTAAGGGTTTATAGAATATT

TCAAATAAAACATCATGTAGGATGGCGTTCAGACAGCAGTTTTCTAACAC

TAATGATTAGAACAGCTGTTTAAGTTCAATATACTATATATGTTTTTATA

TTTAAATAACACGTTTGCTCACCTCTGCAAATATTACACGATAAATGCTA

TATACAAGGCAAGTGGTTTTCCAATCCGATTGTCAATGGGTAAACAAGAT

ACTAGAATATATAAGTATAAATTACTACTGATCAGTTAAACAATAATTCT

GAAGAGCAAGGTGTGACATCATGATACGGCAAGTGATTATCAGCTATTTT

CTCACAGTGTGCTTGTTAGCACTCGTTCAGGTAATGGATCATGAAGTAAG

TCTTTATTAGAAATTACAAATTACAATTATTTTCATGCAGAGTGAGACTG

TGCAAGATTGTGAGAATAAGCTGCCACCGTCGCTGAAGAGTAGACTGTGC

GAGATCCATCT
