# Supplementary material for: Genome-wide transcriptional analyses in Anopheles mosquitoes reveal an unexpected association between salivary gland gene expression and insecticide resistance
Source: BMC Genomics. 2018 Mar 27;19:225. doi: 10.1186/s12864-018-4605-1 (PMC5870100; doi:10.1186/s12864-018-4605-1)
Supplement: Supplementary file 3 — FASTA sequences of region encoding Contig 709 transcript, from Nagongera mosquitoes. (DOCX 20 kb) [file 12864_2018_4605_MOESM3_ESM.docx]

T=Nagongera resistant sample

TC=Nagongera unexposed control sample

TC5J-TC1J low expression, T8-T2 high expression

| sample | Fold change D7r4 expression vs. unexposed controls | 3R:8564156 Genotype |
| --- | --- | --- |
| TC5J | 0.0 | CC |
| TC6J | 0.1 | CC |
| TC14 | 0.8 | CC |
| TC32 | 0.9 | CC |
| TC55 | 1.1 | CC |
| TC7J | 1.5 | CC |
| T3J | 1.6 | CC |
| T4J | 1.8 | CC |
| T13 | 1.9 | CT |
| T17 | 2.1 | CC |
| T8J | 2.2 | CC |
| TC58 | 2.2 | CC |
| TC1J | 2.3 | CC |
| T8 | 3.2 | CT |
| TC50 | 3.6 | CC |
| T2J | 4.0 | CC |
| T14 | 4.5 | CC |
| T4 | 4.9 | CT |
| T3 | 5.4 | CT |
| T10 | 6.2 | CC |
| T16 | 6.6 | CT |
| T18 | 7.6 | CT |
| T11 | 8.3 | CC |
| T7 | 9.2 | CT |
| TC8 | 9.7 | CC |
| T12 | 11.6 | CT |
| T1 | 34.1 | CC |
| T2 | 216.4 | CC |

>TC7J

AAAAAAATGCAGAATNAAGTNGTCAAGAGGAACATATGAATATTTAGCTC

GTAGTTTTCATGAGAGTCGATTGCCAAGAAAACCCACGCCACCTACAACG

GTCCTGATGAGGTGGTTAGCATAGTTCTTAATATAAGTTTTAATATNCAN

NNGAAAATCTTGAGAGAATAAAAGAAGAACATCGATTTTCCATGGCAGCT

GAGAATATTGTAGGAGATCTTCTAGAAAGATACCTATCCATCAGTTCCAC

CATGTCTCTTGCTGCGCTCTGCCACACTATTTGCCATATTCTTGTACTGT

TTCGTGCTGTCGTTGTGCTGTACTACGGAATCTGTACGTCACAATCGGAT

TAAATAAACTGACCCGGATTATCTATCTATCCTTTTCTTCTCTACAGATC

GTAATGCGCAAAACCCCAGCCCCGATCAACGCAGACTCCCATTAACGGCG

AACGAGAGTGTCGATGAGCTGTAAATGACGATCGAAACGACATAAACGCA

GACGCGCTATAAAACCTCAGCCTCGAAAACCGCACCTTGCAGGCAAAAGG

CCCCCGAAGGCACACATAAAACCAGGGTCCCCAAGCCCGCGTAATAGGGA

TTATGTTACACATACGCTTGCACACAAAGGGCCCCAAAATGTCAAAGTTA

CACACAGTGTGTCTCCGTGGCGCTATCTCCGTGACCAACTTCCCAGCGAA

AAGCCACCAACCCTCAGCAGGTGAATTGAAAAAAAGGGAAAACTGAGCCG

CTTCAACAAAACAAGTGCTGCGATGGGTTCGTTGGCTGCCCTTTTTCTCC

GTACCCGAATGCACCTTTTGCTGCTGGACAATGGCTAATTTATTTCAACC

TAAACGCTCTGCTCTGCTCGTAAATTTGTTTAATAGCGTTAATTAAGGTG

AAATAAAATATTAGTTAATTTCAGCTCTAGCACCTCTTCGACCACCAATT

CGTGTGCGATAAATTAGCGATTTTTATGTGATCTCCCACTCCCCCCAAAA

ACTGCCACAGATTAGGCCTGATCTTGCNGAAAAACTCGAGCC

>T16

ATTTAGCTCGTAGTTTTCATGAGAGTCGATTGCCAAGAAACCCACGCCAC

CTACAACGGTTCCTGATGAGGTGGTTAGCATAGTTCTTAATATAAGTTTT

AATATACAGCCNGAAAAATCTTGAGAGAATAAAAGAAGAACATCGATTTT

CCATGGCAGCTGAGAATATTGTAGGAGATCTTCTAGAAAGATACCTATCC

ATCAGTTCCACCATGTCTCTTGCTGCGCTCTGCCACACAATTTGCCATAT

TCTTGTACTGTTTCGTGCTGTCGTTGTGCTGTACTACGGAATCTGTACGT

CACAATCGGATTAAATAAACTGACCCGGATTATCTACCTATCCTTTTCTT

CTCTTCAGATCGTAATGCGCAAAACCCCAGCCCCGATCAACGCAGACTCC

CATTAACTGCGAACGAGAGTGTCGATGAGCTGTAAATGACGATCGAAACG

ACATAAATGCAGACGCGCTATAAAACCTCAGCCTCAAAAACCGCACCTTG

CAGGCAAAAGGCCCCCGAAGGCACACATAAAACCAGAGTCTCCAAGCCCG

CGTAATAGGGATTATGTTACACATACGCTTGCACACAGAGGGCCCCAAAA

TGTCAAAGTTACACACAGTAACTGTGTGTCTCCATGGCGCTATCTCCGTG

ACCAACTTCCCAGCGAAAAACCAAACCCCAGCAGGTGAATTGAAAAAAAG

GGAAAACTGAGCTGCTTCAACAAAACAAGTGCTGCGATGGGTTCGTTGGC

TGCCCTTTTTCTCCGTACCCGAATGCACCTTTAGCTGCTGGACAATGGCT

AATTTATTTCAACCTAAACGCTCTGCTCTGCTCGTAAATTTGTTTAATAG

CGTTAATTAAGGTGAAATAAAATATTAGTTAATTTCAGCTCTAGCACCTC

TTCGACCACCAATTCGTGTGCGATAAATTAGCGATTTTTATGTGATCTCC

CACTCCCCCCAAAAACTGCCACAGATTAGGCCTGATC

>T11

CGGTTCCTGATGAGGTGGTTAGCATAGTTNTTAATATAAGTTTTAATATA

CAGNNGAANATCTTGAGAGAATAAAAGAAGAACATCGATTTTCCATGGCA

GCTGAGAATATTGTAGGAGATCTTCTAGAAAGATACCTATCCATCAGNTC

CACCATGTCTCTTGCTGCGCTCTGCCACACTATTTGCCATATTCTTGTAC

TGTTTCGTGCTGTCGTTGTGCTGTACTACGGAATCTGTACGTCACAATCG

GATTAAATAAACTGACCCGGATTATCTACCTATCCTTTTCTTCTCTACAG

ATCGTAATGCGCAAAACCCCAGCCCCGATCAACGCACACTCCCATTAACT

GCGAACGAGAGTGTCGATAAGCTGTAAATGACGTTCGAAACGACATAAAT

GCAGACGCGCTATAAAACCTCAGCCTCAAAAACCGCACCTTGCAGGCAAA

AGGCCCCTGAAGGCACACATAAAACCGTAGTCCCAAAGCCCGCGTAATAG

GGATTATGTTACACATACGCTTGCACACAGAGGGCTCCAAAATGTCAAAG

TTACACGCGTGTGGAGTGTCTCCGTGGCGCTATCTCCGTGACCAACTTCC

CAGCGAAAAGCCACCAACCCCCAGCAGGTGAATTAAAAAAAGGGAAAACT

GAGCCGCTTCAACAAAACAAGTGCTGCGATGGTTCGTTGGCTGCCCTTTT

TCTCCGTACCCAAATGCACCTTTAGCTGCCGGACAATGGCTAATTTATTT

CAACCTAAACGCTCTGCTCTGCTCGTAAATTTGTTTAATAGCGTTAATTA

AGGTGAAATAAAATATTAGTTAATTTCAGCTCTAGCACCTCTCCGACCAC

CAATTCGTGTGCGATAAATTAGCGATTTTTATGTGATCTCCCACTATCCC

CCAAAAACTGCCACAGATTAGGCCTGATC

>T1

GATGAGGTGGTTAGCATAGTTNTTAATATAAGTTTTAATATACAGNNGAA

AATCTTGAGAGAATAAAAGAAGAACATCGATTTTCCATGGCAGCTGAGAA

TATTGTAGGAGATCTTCTAGAAAGATACCTATCCATCAGTTCCACCATGT

CTCTTGCTGCGCTCTGCCACACAATTTGCCATATTCTTGTACTGTTTCGT

GCTGTCGTTGTGCTGTACTACGGAATCTGTACGTCACAATCGGATTAAAT

AAACTGACCCGGATTATCTACCTATCCTTTTCTTCTCTACAGATCGTAAT

GCGCAAAACCCCAGCCCCGATCAACGCAGACTCCCATTAACGGCGAACGA

GAGTGTCGATGAGCTGTAAATGACGTTCGAAACGACATAAATGCAGACGC

GCTATAAAACCTCAGCCTCAAAAACCGCACCTTGCAGGCAAAAGGCCCCC

GAAGGCACACATAAAACCGTGGTCCCCAAGCCCGCGTAATAGGGATTATG

TTACCCATACGCTTGCACACAGAGGGCCCCAAAATGTCAAAGTTACACGC

GTGTGTCTCCGTGGCGCTATCTCCGTCACCAACTTCCCAGCGAAAAACCA

CCAACCCCCAGCAGGTGAATTAAAAAAAGGGAAAACTGAGCCGCTTCAAC

AAAACAAGTGCTGCGATGGTTCGTTGGCTGCCCTTTTTCTCCGTACCCAA

ATGCACCTTTAGCTGCCGGACAATGGCTAATTTATTTCAACCTAAACGCT

CTGCTCTGCTCGTAAATTTGTTTAATAGCGTTAATTAAGGTGAAATAAAA

TATTAGTTAATTTCAGCTCTAGCACCTCTTCGACCACCAATTCGTGTGCG

ATAAATTAGCGATTTTTATGTGATCTCCCACTCTCCCCCGACAAAAAAAA

AAACGCACTGCCACAGATTAGGCCTGATC

>TC1J

GAAAATCTTGAGAGAATAAAAGAAGAACATGGTTTTCCAGGCAGTGAGAA

TATGTAGGAGATNTTCTAGAAAAGATACCTATCCATCAGTTCCACCATGT

CTCTTGNTGCGNTCTGCCACACAATTTGCCATATTCTTGTACTGTTTCGT

GCNGTCGTTGTGCTGTACTACGGAATCTGTACGTCACAATNGGATTAAAT

AAACTGACCCGGATTATCTACCTATCCTTTTCTTCTCTACAGATCGTAAT

GCGCAAAACCCCAGCCCCGATCAACGCAGACTCCCATTAACTGCGAACGA

GAGTGTCGATGAGCTGTAAATGACGATCGAAACGACATAAAATGCAGACG

CGCTATAAAACCTCAGCCTCAAAAACCGCACCTTGCAGGCAAAAGGCCCC

CAAAGGCACACATAAAACCGTGGTACCAAAGCCCGCGTAATAGGGATTAT

GTTACCCATACGCTTGCACACAGAGGGCCCCAAAATGTCAAAGTTACACG

CGTGTGGAGTGTCTCCGTGGCGCTATCTCCGTGACCAACTTCCCAGCGAA

AAGCCACCAACCCCCAGCAGGTGAATTAAAAAAAGGGAAAACTGAGCCGC

TTCAACAAAACAAGTGCTGCGATGGTTCGTTGGCTGCCCTTTTTCTCCGT

ACCCAAATGCACCTTTAGCTGCCGGACAATGGCTAATTTATTTCAACCTA

AACGCTCTGCTCTGCTCGTAAATTTGTTTAATAGCGTTAATTAAGGTGAA

ATAAAATATTAGTTAATTTCAGCTCTAGCACCTCTTCGACCACCAATTCG

TGTGCGATAAATTAGCGATTTTTATGTGATCTCCCACTCTCCCCCGACAA

AAAAAAACGCACTGCCACAGATTAGGCCTGATCTTGCNGAAAAACTCGAG

CC

>T17

GGCTCGAGTTTTTCNGCAAGATACCTATCCATCAGTTCCACCATGTCTCT

TGCTGCGCTCTGCCACACAATTTGCCATATTCTTGTACTGTTTCGTGCTG

TCGTTGTGCTGTACTACGGAATCTGTACGTCACAATCGGATTAAATAAAC

TGACCCGGATTATCTATCTATCCTTTTCTTCTCTACAGATCGTAATGCGC

AAAACCCCAGCCCCGATCAACGCAGACTCCCATTAACGGCGAACGATAGT

GTCGATGAGCTGTAAATGACGATCGAAACGACATAAATGCAGACGCGCTA

TAAAACCTCAGCCTCAAAAACCGCACCTTGCAGGCAAAAGGCCCCCGAAG

GCACACATAAAACCAGGGTCTCCAAGCCCGCGTAATAGGGATTATGTTAC

ACATACGCTTGCACACAGAGGGCCCCAAAATGTCAAAGTTACACACAGTG

TGTCTCCGTGGCGCTATCTCCGTGACCAACTTCCCAGCGAAAAACCAACC

CCCAGCAGGTGAATTGAAAAAAAGGGAAAACTGAGCCTCTTCAACAAAAC

AAGTGCTGCGATGGGTTCGTTGGCTGCCCTTTTTCTCCGTACCCGAATGC

ACCTTTAACTGCTGGACAATGGCTAATTTATTTCAACCTAAACGCTCTGC

TCTGCTCGTAAATTTGTTTAATAGCGTTAATTAAGGTGAAATAAAATATT

AGTTAATTTCAGCTCTAGCACCTCTTCGACCACCAATTCGTGTGCGATAA

ATTAGCGATTTTTATGTGATCTCCCACTCTCCCCCGACAAAAAAAACGCA

CTGCCACAGATTANGCCTGATCTTTCTAGAAGATCTCCTACAATATTCTC

AGCTGCCATGGAAAATCGATGTTCTTCTTTTATTCTCTCAAGATTTTCNG

GCTGTATATTAAAACTTATATTAAGAACTATGCTAACCACCTCATC

>TC14

GGCTCGAGTTTTTCNGCAAGATACCTATCCATCAGTTCCACCATGTCTCT

TGCTGCGCTCTGCCACACAATTTGCCATATTCTTGTACTGTTTCGTGCTG

TCGTTGTGCTGTACTACGGAATCTGTACGTCACAATCGGATTAAATAAAC

TGACCCGGATTATCTACCTATCCTTTTCTTCTCTTCAGATCGTAATGCGC

AAAACCCCAGCCCCGATCAACGCAGACTCCCATTAATTGCGAACGAGAGT

GTCGATGAGCTGTAAATGACGATCGAAACGACATAAATGCAGACGCGCTA

TAAAACCTCAGCCTCAAAAACCGCACCTTGCAGGCAAAAGGCCCCCGAAG

GCACACATAAAACCGGGGTTCCAAAGCCCGCGTAATAGGGATTATGTTAC

ACATACGCTTGCACACAGAGGGCTCCAAAATGTCAAAGTTACACGCGTGT

GTCTCCGTGGCGCTATCTCCGTGACCAACTTCCCAGCGAAAAGCCACCAA

CCCCCAGCAGGTGAATTGAAAAAAAGGGAAAACTGAGCCGCTTCAACAAA

ACAAGTGCTGCGATGGGTCGTTGGCTGCCCTTTTTCTCCGTACCCAAATG

CACCTTTAGCTGCCGGACAATGGCTAATTTATTTCAACCTAAACGCTCTG

CTCTGCTCGTAAATTTGTTTAATAGCGTTAATTAAGGTGAAATAAAATAT

TAGTTAATTTCAGCTCTAGCACCTCTCCGACCACCAATTCGTGTGCGATA

AATTAGCGATTTTTATGTGATCTCCCACTATCCCCCAAAAACTGCCACAG

ATTANGCCTGATCTTTCTAGAAGATCTCCTACAATATTCTCAGCTGCCAT

GGAAAATCGATGTTCTTCTTTTATTCTCTCNAGATTTTCNNCTGTATATT

AAAACTTATATTAAGAACTATGCTAACCACCTCATCNNNACCGTTGTAGG

TGGCGTGGGTTTTCTTGGCAATCGACTCTCATGAAAACTACGAGCTAAAT

ATTCAATATG

>TC55

GGCTCGAGTTTTTCNGCAAGATACCTATCCATCAGTTCCACCATGTCTCT

TGCTGCGCTCTGCCACACTATTTGCCATATTCTTGTACTGTTTCGTGCTG

TCGTTGTGCTGTACTACGGAATCTGTACGTCACAATCGGATTAAATAAAC

TGACTCGGATTATCTATCTATCCTTTTCTTCTCTACAGATCGTAATGCGC

AAAACCCCAGCCCCGATCAACGCAGACTCCCATTAACTGCGAACGAGAGT

GTCGATGAGCTGTAAATGACGATCGAAACGACATAAAATGCAGACGCGCT

ATAAAACCTCAGCCTCAAAAACCGCACCTTGCAGGCAAAAGGCCCCCGAA

GGCACACATAAAACCGTGGTCCCAAAGCCCGCGTAATAGGGATTATGTTA

CACATACGCTTGCACACAGAGGGCTCCAAAATGTCAAAGTTACACGCGTG

TGTCTTCGTGGCGCTATCTCCGTGACCAACTTCCCAGCAAAAAACCAAAC

CCCAGCAGGTGAATTGAAAAAAAGGGAAAACTGAGCTGCTTCAACAAAAC

AAGTGCTGCGATGGGTTCGTTGGCTGCCCTTTTTCTCCGTACCCGAATGC

ACCTTTAGCTGCTGGACAATGGCTAATTTATTTCAACCTAAACGCTCTGC

TCTGCTCGTAAATTTGTTTAATAGCGTTAATTAAGGTGAAATAAAATATT

AGTTAATTTCAGCTCTAGCACCTCCCCGACCACCAATTCGTGTGCGATAA

ATTAGCGATTTTTATGTGATCTCCCACTCCCCCTCCGACAAAAAAAACGC

ACTGCCACAGATTANGCCTGATCTTTCTAGAAGATCTCCTACAATATTCT

CAGCTGCCATGGAAAATCGATGTTCTTCTTTTATTCTCTCAAGATTTTCA

GGCTGTATATTAAAACTTATATTAANAACTATGCTAACCACCTCATC

>T4

GGCTCGAGTTTTTCNGCAAGATACCTATCCATCAGTTCCACCATGTCTCT

TGCTGCGCTCTGCCACACAATTTGCCATATTCTTGTACTGTTTCGTGCTG

TCGTTGTGCTGTACTACGGAATCTGTACGTCACAATCGGATTAAATAAAC

TGACCCGGATTATCTACCTATCCTTTTCTTCTCTACAGATCGTAATGCGC

AAAACCCCAGCCCCGATCAACGCAGACTCCCATTAACGGCGAACGAGAGT

GTCGATGAGCTGTAAATGACGATCGAAACGACATAAATGCAGACGCGCTA

TAAAACCTCAGCCTCAAAAACCGCACCTTGCAGGCAAAAGGCCCCCGAAG

GCACACATAAAACCGGGGTCCCAAAGCCCGCGTAATAGGGATTATGTTAC

ACATACGCTTGCACACAGAGGGCCCCAAAATGTCAAAGTTACACGCGTGT

GGAGTGTCTCCATGGCGCTATCTCCGTGACCAACTTCCCAGCGAAAAGCC

ACCAACCCCCAGCAGGTGAATTTTAAAAAAAGGGAAAACTGAGCCGCTTC

AACAAAACAAGTGCTGCGATGGTTCGTTGGCTGCCCTTTTTTTCCGTACC

CAAATGCACCTTTAGCTGCCGGACAATGGCTAATTTATTTCAACCTAAAC

GCTCTGCTCTGCTCGTAAATTTGTTTAATAGCGTTAATTAAGGTGAAATA

AAATATTAGTTAATTTCAGCTCTAGCACCTCTTCGACCACCAATTCGTCT

GCGATAAATTAGCGATTTTTATGTGATCTCCCACTNNNAAAAAAACGCAC

TGCCACAGATTANGCCTGATCTTTCTAGAAGATCTCCTACAATATTCTCA

GCTGCCATGGAAAATCGATGTTCTTCTTTTATTCTCTCAAGATTTTCNGG

CTGTATATTAAAACTTATATTAAGAACTATGCTAACCACCTCATC

>TC50

GGCTCGAGTTTTTCNGCAAGATACCTATCCNTCAGTTCCACCATGTCTCT

TGCTGCGCTCTGCCACACTATTTGCCATATTCTTGTACTGTTTCGTGCTG

TCGTTGTGCTGTACTACGGAATCTGTACGTCACAATCGGATTAAATAAAC

TGACCCGGATTATCTACCTATCCTTTTCTTCTCTACAGATCGTAATGCGC

AAAACCCCAGCCCCGATCAACGCACACTCCCATTAACGGCGAACGAGAGT

GTCGATGAGCTGTAAATGACGATCGAAACGACATAAATGCAGACGCGCTA

TAAAACCTCAGCCTCCAAAAAACCGCACCTTGCAGGCAAAAGGCCCCCGA

AGGCACACATAAAACCGTGGTCCCAATGCCCGCGTAATAGGGATTATGTT

ACCCATACGCTTGCACACAGAGGGCTCCAAAATGTCAAAGTTACACGCGT

GTGTCTCCGTGGCGCTATCTCTGTGACCAACTTCCCAGCGAAAAGCCACC

AACCCCCAGCAGGTGAATTAAAAAAAGGGAAAACTGAGCCGCTTCAACAA

AACAAGTGCTGCGATGGGTTCGTTGGCTGCCCTTTTTCTCCGTACTCGAA

TGCACCTTTAGCTGCTGGACAATGGCTAATTTATTTCAACCTAAACGCTC

TGCTCTGCTCGTAAATTTGTTTAATAGCGTTAATTAAGGTGAAATAAAAT

ATTAGTTAATTTCAGCTCTAGCACCTCTCCGACCACCAATTCGTGTGCGA

TAAATTAGCGATTTTTATGTGATCTCCCACTCCCCCCAAAAACTGCCACA

GATTANGCCTGATCTTTCTAGAAGATCTCCTACAATATTCTCAGCTGCCA

TGGAAAATCGATGTTCTTCTTTTATTCTCTCAAGATTTTCNGGCTGTATA

TTAAAACTTATATTAAGAACTATGCTAACCACCTCATCNNNANCCGTTGT

AGNTGGCGTGGGTTTTCTTGGCCAATCGACTCTCATGAAAACTACGAGCT

AAATTA

>T2J

GGCTCGAGTTTTTCNGCAAGATACCTATCCATCAGTTCCACCATGTCTCT

TGCTGCGCTCTGCCACACAATTTGCCATATTCTTGTACTGTTTCGTGCTG

TCGTTGTACTGTACTACGGAATCTGTACGTCACAATCGGATTAAATAAAC

TGACCCGGATCATCTACCTATCCTTTTCTTCTCTTCAGATCGTAATGCGC

AAAACCCCAGCCCCGATCAACGCAGACTCCCATTAACTGCGAACCAGAGT

GTCGATGAGCTGTAAATGACGTTCGAAACGACATAAATGCAGACGCGCTA

TAAAACCTCAGCCTCAAAAACCGCACCTTGCAGGCAAAAGGCCCCCGAAG

GCACACATAAAACCGTGATCCCAAAGCCCGCGTAATAGGGATTATGTTAC

ACATACGCTTGCACACAGAGGGCTCCAAAATGTCAAAGTTACACGCGTGT

GTCTCCGTGGCGCTATCTCCGTGACCAACTTCCCAGCGAAAAACCAAACC

CCAGCAGGTGAATAAAAAAGGGAAAACTGAGCCGCTTCAACAAAACAAGT

GCTGCGATGGGTCGTTGGCTGCCCTTTTTCTCCGTACCCGAATGCACCTT

TAGCTGCTGGACAATGGCTAATTTATTTCAACCTAAACGCTCTGCTCTGC

TCGTAAATTTGTTTAATAGCGTTAATTAAGGTGAAATAAAATATTAGTTA

ATTTCAGCTCTAGCACCTCTTCGACCACCAATTCGTGTGCGATAAATTAG

CGATTTTTATGTGATCTCCCACTCCCCCCAAAAACTGCCACAGATTNNCC

TGATCTTTCTAGAAGATCTCCTACAATATTCTCAGCTGCCATGGAAAATC

GATGTTCTTCTTTTATTCTCTCAAGANTTTCNNCTGTATATTAAAACTTA

TATTAAGAACTATGCTAACCACCTCATCNNNACCGTTGTANGTGGCGTGG

GTTTTCTTGGCAATCGACTCTCATGAAACTACGAGCTAATATTCATATGT

TCCTCTTGACCAACTTTANTCTGCATTTTTTTGACGAGGTTTA

>T3

GGCTCGAGTTTTTCNGCAAGATACCTATCCATCAGTTCCACCATGTCTCT

TGCTGCGCTCTGCCACACAATTTGCCATATTCTTGTACTGTTTCGTGCTG

TCGTTGTGCTGTACTACGGAATCTGTACGTCACAATCGGATTAAATAAAC

TGACCCGGATTATCTATCTATCCTTTTCTTCTCTACAGATCGTAATGCGC

AAAACCCCAGCCCCGATCAACGCAGACTCCCATTAACGGCGAACGAGAGT

GTCGATGAGCTGTAAATGACGTTCGAAACGACATAAATGCAGACGCGCTA

TAAAACCTCAGCCTCAAAAACCGCACCTTGCAGGCAAAAGGCCCCCGAAG

GCACACATAAAACCAGGGTCCCCAAGCCCGCGTAATAGGGATTATGTTAC

ACATACGCTTGCACACAGAGGGCCCCAAAATGTCAAAGTTACACGCGTGT

GGAGTGTCTCCATGGCGCTATCTCCGTGACCAACTTCCCAGCGAAAAGCC

ACCAACCCCCAGCAGGTGAATTAAAAAAAAGGGAAAACTGAGCTGCTTCA

ACAAAACAAGTGCTGCGATGGGTCGTTGGCTGCCCTTTTTGTCCGTACCC

GAATGCACCTTTAGCTGCTGGACAATGGCTAATTTATTTCAACCTAAACG

CTCTGCTCTGCTCGTAAATTTGTTTAATAGCGTTAATTAAGGTGAAATAA

AATATTAGTTAATTTCAGCTCTAGCACCTCTTCGACCACCAATTCGTGTG

CGATAAATTAGCGATTTTTATGTGATCTCCCACTCCCCCCAAAAACTGCC

ACAGATTAGGCCTGATCTTTCTAGAAGATCTCCTACAATATTCTCAGCTG

CCATGGAAAATCGATGTTCTTCTTTTATTCTCTCAAGATTTTCANNTGTA

TATTAAAACTTATATTAANAACTATGCTAACCACCTCATCAGGNACCGTT

GTAGGTGGCGTGGGTTTTCTTGGCAATCNNCTCTCATGAAACTACNANCT

AAATATTCATATGTTCCTCTTGACCAACTTTATTCTGCATTTTTTTGACG

AGTTTA

>T4J

GGCTCGAGTTTTTCNGCAAGATACCTATCCATCAGTTCCACCATGTCTCT

TGCTGCGCTCTGCCACACAATTTGCCATATTCTTGTACTGTTTCGTGCTG

TCGTTTTGCTGTACTACGGAATCTGTACGTCACAATCGGATTAAATAAAC

TGACCCGGATTATCTACCTATCCTTTTCTTCTCTACAGATCGTAATGCGC

AAAACCCCAGCCCCGATCAACGCAGACTCCCATTAACTGCGAACGAGAGT

GTCGATGAGCTGTAAATGACGTTCGAAACGACATAAATGCAGACGCGCTA

TAAAACTTCAGCCTCAAAAACCGCACCTTACAGGCAAAAGGCCCCCGAAG

GCACACATAAAACCAGGGTCTCCAAGCCCGCGTAATAGGGATTATGTTAC

ACATACGCTTGCACACAGAGGGCTCCAAAATGTCAAAGTTACACGCGTGT

GTCTCCGTGGCGCTATCTCCGTGACCAACTTCCCAGCGAAAAACCAAACC

CCAGCAGGTGAATTAAAAAAAGGGAAAACTGAGCCGCTTCAACAAAACAA

GTGCTGCGATGGGTTCGTTGGCTGCCCTTTTTCTCCGTACTCGAATGCAC

CTTTAGCTGCTGGACAATGGCTAATTTATTTCAACCTAAACGCTCTGCTC

TGCTCGTAAATTTGTTTAATAGCGTTAATTAAGGTGAAATAAAATATTAG

TTAATTTCAGCTCTAGCACCTCTCCGACCACCAATTCGTGTGCGATAAAT

TAGCGATTTTTATGTGATCTCCCACTCCCCCCAAAAACTGCCACAGATTA

NGCCTGATCTTTCTAGAAGATCTCCTACAATATTCTCAGCTGCCATGGAA

AATCGATGTTCTTCTTTTATTCTCTCAAGATTTTCACGCTGTATATTAAA

ACTTATATTAAGAACTATGCTAACCACCTCATCNNAACCGTTGTANTGGC

GTGGGTTTTCTTGGCAATCGACTCTCATGAAAACTACGAGCTAATATTCA

ATATGTTCCTCTTGACCAACTTTATTCTGCATTTTTTTGACGAGTTTA

>T8J

GGCTCGAGTTTTTCNGCAAGATACCTATCCATCAGTTCCACCATGTCTCT

TGCTGCGCTCTGCCACACAATTTGCCATATTCTTGTACTGTTTCGTGCTG

TCGTTGTGCTGTACTACGGAATCTGTACGTCACAATCGGATTAAATAAAC

TGACTCGGATTATCTATCTATCCTTTTCTTCTCTACAGATCGTAATGCGC

AAAACCCCAGCCCCGATCAACGCAGACTCCCATTAACTGCGAACGAGAGT

GTCGATGAGCTGTAAATGACGATCGAAACGACATAAAATGCAGACGCGCT

ATAAAACCTCAGCCTCAAAAACCGCACCTTGCAGGCAAAAGGCCCCCGAA

GGCACACATAAAACCGTGGTCCCAAAGCCCGCGTAATAGGGATTATGTTA

CACATACGCTTGCACACAGAGGGCTCCAAAATGTCAAAGTTACACGCGTG

TGTCTTCGTGGCGCTATCTCCGTGACCAACTTCCCAGCAAAAAACCAAAC

CACAGCAGGTGAATTAAAAAAAGGGAAAACTGAGCTGCTTCAACAAAACA

AGTGCTGCGATGGGTTCGTTGGCTGCCCTTTTTCTCCGTACCCGAATGCA

CCTTTAGCTGCTGGACAATGGCTAATTTATTTCAACCTAAACGCTCTGCT

CTGCTCGTAAATTTGTTTAATAGCGTTAATTAAGGTGAAATAAAATATTA

GTTAATTTCAGCTCTAGCACCTCCCCGACCACCAATTCGTGTGCGATAAA

TTAGCGATTTTTATGTGATCTCCCACTATCCCCCAAAAACTGCCACAGAT

TANGCCTGATCTTTCTAGAAGATCTCCTACAATATTCTCAGCTGCCATGG

AAAATCGATGTTCTTCTTTTATTCTCTCAAGATTTTCNNNTGTATATTAA

AACTTATATTAAGAACTATGCTAACCACCTCATCNNAACCGTTGTAGNNG

GCGTGGGTTTTCTTGGCAATCGACTCTCATGAAAACTACGAGCTAATATT

CAATATGTTCCTCTTGACCAACTTTATTCTGCATTTTTTTGACGAG

>T8

GGCTCGAGTTTTTCNGCAAGATACCTATCCATCAGTTCCACCATGTCTCT

TGCTGCGCTCTGCCACACAATTTGCCATATTCTTGTACTGTTTCGTGCTG

TCGTTGTGCTGTACTACGGAATCTGTACGTCACAATCGGATTAAATAAAC

TGACCCGGATTATCTACCTATCCTTTTCTTCTCTACAGATCGTAATGCGC

AAAACCCCAGCCCCGATCAACGCAGACTCCCATTAACGGCGAACGAGAGT

GTCGATGAGCTGTAAATGACGATCGAAACGACATAAATGCAGACGCGCTA

TAAAACCTCAGCCTCAAAAACCGCACCTTGCAGGCAAAAGGCCCCCGAAG

GCACACATAAAACCAGGGTCTCCAAGCTCGCGTAATAGGGATTATGTTAC

ACATACGCTTGAACACAGAGGGCCCCAAAATGTCAAAGTTACACACAGTA

ACTGTGTGTCTCCATGGCGCTATCTCCGTGACCAACTTCCCAGCGAAAAA

CCAAACCCCAGCAGGTGAATTGAAAAAAAGGGAAAACTGAGCTGCTTCAA

CAAAACAAGTGCTGCGATGGGTTCGTTGGCTGCCCTTTTTCTCCGTACCC

GAATGCACCTTTAGCTGCTGGACAATGGCTAATTTATTTCAACCTAAACG

CTCTGCTCTGCTCGTAAATTTGTTTAATAGCGTTAATTAAGGTGAAATAA

AATATTAGTTAATTTCAGCTCTAGCACCTCTCCGACCACCAATTCGTGTG

CGATAAATTAGTGATTTTTATGTGATCTCCCACTCCCCAAAAAAACGCAC

TGCCACAGATTANGCCTGATCTTTCTAGAAGATCTCCTACAATATTCTCA

GCTGCCATGGAAAATCGATGTTCTTCTTTTATTCTCTCAAGATTTTCNNN

TGTATATTAAAACTTATATTAANAACTATGCTAACCACCTCATCAGAACC

GTTGTAGTGGCGTGNNTTTCTTGGCAATCGACTCTCATGAAAACTACGAG

CTAAATATTCAATATGTTCCTCTTGACCAACTTTATTCTGCATTTTTTTG

ACGAGNTTAGAGCAAGCTTC

>TC58

GGCTCGAGTTTTTCNGCAAGATACCTATCCATCAGTTCCACCATGTCTCT

TGCTGCGCTCTGCCACACAATTTGCCATATTCTTGTACTGTTTCGTGCTG

TCGTTTTGCTGTACTACGGAATCTGTACGTCACAATCGGATTAAATAAAC

TGACCCGGATTATCTACCTATCCTTTTCTTCTCTACAGATCGTAATGCGC

AAAACCCCAGCCCCGATCAACGCAGACTCCCATTAACTGCGAACGAGAGT

GTCGATGAGCTGTAAATGACGTTCGAAACGACATAAATGCAGACGCGCTA

TAAAACTTCAGCCTCAAAAACCGCACCTTACAGGCAAAAGGCCCCCGAAG

GCACACATAAAACCAGGGTCTCCAAGTCCGCGTAATAGGGATTATGTTAC

ACATACGCTTGCACACAGAGGGCTCCAAAATGTCAAAGTTACACGCGTGT

GTCTCCGTGGCGCTATCTCCGTGACCAACTTCCCAGCGAAAAACCAAACC

CCAGCAGGTGAATAAAAAAGGGAAAACTGAGCCGGTTCAACAAAACAAGT

GCTGCGATGGGTCGTTGGCTGCCCTTTTTCTCCGTACCCGAATGCACCTT

TAGCTGCTGGACAATGGCTAATTTATTTCAACCTAAACGCTCTGCTCTGC

TCGTAAATTTGTTTAATAGCGTTAATTAAGGTGAAATAAAATATTAGTTA

ATTTCAGCTCTAGCACCTCTTCGACCACCAATTCGTGTGCGATAAATTAG

CGATTTTTATGTGATCTACCACTCCCCCTCCGACAAAAAAAAACGCACTG

CCACAGATTANGCCTGATCTTTCTAGAAGATCTCCTACAATATTCTCAGC

TGCCATGGAAAATCGATGTTCTTCTTTTATTCTCTCAAGATTTTCNNNTG

TATATTAAAACTTATATTAAGAACTATGCTAACCACCTCATCAGAACCGT

TGTAGTGGCGTGGGTTTTCTTGGCATCGACTCTCATGAAAACTACGAGCT

AATATTCAATATGTTCCTCTTGACCAACTTTATCTGCATTTTTTTGACGA

GGTTAGAGCAAGCTTCNGGAAACTGAGACAGGAATTTTATTAAAATTTAA

ATTTGAAGAA

>TC5J

GGCTCGAGTTTTTCNGCAAGATACCTATCCATCAGTTCCACCATGTCTCT

TGCTGCGCTCTGCCACACAATTTGCCATATTCTTGTACTGTTTCGTGCTG

TCGTTGTGCTGTACTACGGAATCTGTACGTCACAATCGGATTAAATAAAC

TGACCCGGATTATCTATCTATCCTTTTCTTCTCTACAGATCGTAATGCGC

AAAACCCCAGCCCCGATCAACGCAGACTCCCATTAACGGCGAACGATAGT

GTCGATGAGCTGTAAATGACGATCGAAACGACATAAATGCAGACGCGCTA

TAAAACCTCAGCCTCAAAAACCGCACCTTGCAGGCAAAAGGCCCCCGAAG

GCACACATAAAACCAGGGTCTCCAAGCCCGCGTAATAGGGATTATGTTAC

ACATACGCTTGCACACAGAGGGCCCCAAAATGTCAAAGTTACACACAGTG

TGTCTCCGTGGCGCTATCTCCGTGACCAACTTCCCAGCGAAAAACCAACC

CCCAGCAGGTGAATTGAAAAAAAGGGAAAACTGAGCCTCTTCAACAAAAC

AAGTGCTGCGATGGGTTCGTTGGCTGCCCTTTTTCTCCGTACCCGAATGC

ACCTTTAACTGCTGGACAATGGCTAATTTATTTCAACCTAAACGCTCTGC

TCTGCTCGTAAATTTGTTTAATAGCGTTAATTAAGGTGAAATAAAATATT

AGTTAATTTCAGCTCTAGCACCTCTCCGACCACCAATTCGTGTGCGATAA

ATTAGCGATTTTTATGTGATCTCCCACTATCCCCCAAAAACTGCCACAGA

TTAGGCCTGATCTTTCTAGAAGATCTCCTACAATATTCTCAGCTGCCATG

GAAAATCGATGTTCTTCTTTTATTCTCTCAAGATTTTCNNCTGTATATTA

AAACTTATATTAAGAACTATGCTAACCACCTCATCNNAACCGTTGTAGGN

GGCGTGGGTTTTCTTGGCAATCGACTCTCATGAAAACTACGAGCTAAATA

TTCAATATGTTCCTCTTGACCAACTTTATTCTGCATTTTTTTGAACGAGG

TTTAGAGCAAGCTTCNNAAACTGAGACAGGAATTTTATTAAAAATTTAAA

TTT

>T14

GGCTCGAGTTTTTCNGCAGATACCTATCCATCAGTTCCACCATGTCTCTT

GCTGCGCTCTGCCACACAATTTGCCATATTCTTGTACTGTTTCGTGCTGT

CGTTGTGCTGTACTACGGAATCTGTACGTCACAATCGGATTAAATAAACT

GACCCGGATTATCTACCTATCCTTTTCTTCTCTACAGATCGTAATGCGCA

AAACCCCAGCCCCGATCAACGCAGACTCCCATTAACGGCGAACGAGAGTG

TCGATGAGCTGTAAATGACGATCGAAACGACATAAATGCAGACGCGCTAT

AAAACCTCAGCCTCGAAAACCGCACCTTGCAGGCAAAAGGCCCCCGAAGG

CACACATAAAACCGTGGTCCCCAAGCCCGCGTAATAGGGATTATGTTACC

CATACGCTTGCACACAGAGGGCCCCAAAATGTCAAAGTTACACGCGTGTG

TCTCCGTGGCGCTATCTATGTGACCAACTTCCCAGCGAAAAGCCACCAAC

CCCCAGCAGGTGGATTAAAAAAAGGGAAAACTGAGCCGCTTCAACAAAAC

AAGTGCTGCGATGGTTCGTTGGCTGCCCTTTTTCTCCGTACCCAAATGCA

CCTTTAGCTGCTGGACAATGGCTAATTTATTTCAACCTAAACGCTCTGCT

CTGCTCGTAAATTTGTTTAATAGCGTTAATTAAGGTGAAATAAAATATTA

GTTAATTTCAGCTCTAGCACCTCTTCGACCACCAATTCGTGTGCGATAAA

TTAGCGATTTTTATGTGATCTCCCACTCCCCCCAAAAACTGCCACAGATT

ANGCCTGATCTTTCTAGAAGATCTCCTACAATATTCTCAGCTGCCATGGA

AAATCGATGTTCTTCTTTTATTCTCTCAAGATTTTCNNCTGTATATTAAA

ACTTATATTAAGAACTATGCTAACCACCTCATCNGNACNGTTGTAGGTGG

CGTGGNTTTTCTTGGCAATCGACTCTCATGAAACTACGAGCTAATATTCA

NATGTTCCTCTTGACCA

>TC6J

TCGAGTTTTTCNGCAAGATACCTATCCATCAGTTCCACCATGTCTCTTGC

TGCGCTCTGCCACACAATTTGCCATATTCTTGTACTGTTTCGTGCTGTCG

TTGTGCTGTACTACGGAATCTGTACGTCACAATCGGATTAAATAAACTGA

CCCGGATTATCTACCTATCCTTTTCTTCTCTACAGATCGTAATGCACAAA

ACCCCAGCCCCGATCAACGCAGACTCCCATTAACTGCGAACGAGAGTGTC

GATGAGCTGTAAATGACGTTCGAAACGACATAAATGCAGACGCGCTATAA

AACCTCAGCCTCAAAAACCGCACCTTGCAGGCAAAAGGCCCCCGAAGGCA

CACATAAAACCAGAGTCTCCAAGCCCGCGTAATAGGGATTATGTTACACA

TACGCTTGCACACAGAGGGCCCCAAAATGTCAAAGTTACACGCGTGTGTC

TCCGTGGCGCTATCTCCGTGACCAACTTCCCATAGAAAAACCAACCCCCA

GCAGGTGAATTAAAAAAAAGGGAAAACTGAGCTGCTTCAACAAAACAAGT

GCTGCGATGGATCGTTGGCTGCCCTTTTTCTCCGTACCCGAATGCACCTT

TTGCTGCTGGACAATGGCTAATTTATTTCAACCTAAACGCTCTGCTCTGC

TCGTAAATTTGTTTAATAGCGTTAATTAAGGTGAAATAAAATATTAGTTA

ATTTCAGCTCTAGCACCTCTTCGACCACCAATTCGTGTGCGATAAATTAG

CGATTTTTATGTGATCTCCCACTCCCCAAAAAAACGCACTGCCACAGATT

ANGCCTGATCTTTCTAGAAGATCTCCTACAATATTCTCAGCTGCCATGGA

AAATCGATGTTCTTCTTTTATTCTCTCAAGATTTTCNNNTGTATATTAAA

ACTTATATTAAGAACTATGCTAACCACCTCATCNGAACCGTTGTAGTGGC

GTGGGTTTTCTTGGCAATCGACTCTCATGAAAACTACNAGCTAATATTCA

TATGTTCCTCTTGACCAACTTTATTCTGCATTTTTTTGACGAGTTTA

>T10

GCAAGATACCTATCCATCAGTTCCACCATGTCTCTTGCAGCGCTCTGCCA

CACAATTTGCCATATTCTTGTACTGTTTCGTGCTGTCGTTGTGCTGTACT

ACGGAATCTGTACGTCACAATCGGATTAAATAAACTGACCCGGATTATCT

ACCTATCCTTTTCTTCTCTACAGATCGTAATACGCAAAACCCAAGCCCCG

ATCAACGCAGACTCCCATTAACGGCGAACGAGAGTGTCGATGAGCTGTAA

ATGACGATTGAAACGACATAAATGCAGACGCGATATAAAACCTCAGCCTC

AAAAACCGCACCTTGCAGGCAAAAGGCCCCTGAAGGCACACATAAAACCG

TAGTCCCAAAGCCCGCGTAATAGGGATTATGTTACACATACGCTTGCACA

CAGAGGGCTCCAAAATGTCAAAGTTACACGCGTGTGTCTCCGTGGCGCTA

TCTCCGTGACCAACTTCCCAGCGAAAAGCCACCAACCCCCAGCAGGTGAA

TTAAAAAAAGGGAAAACTGAGCCGCTTCAACAAAACAAGTGCTGCGATGG

TTCGTTGGCTGCCCTTTTTCTCCGTACCCAAATGCACCTTTAGCTGCCGG

ACAATGGCTAATTTATTTCAACCTAAACGCTCTGCTCTGCTCGTAAATTT

GTTTAATAGCGTTAATTAAGGTGAAATAAAATATTAGTTAATTTCAGCTC

TAGCACCTCTCCGACCACCAATTCGTGTGCGATAAATTAGCGATTTTTAT

GTGATCTCCCACTATCCCCCAAAAACTGCCACAGATTANGCCTGATCTTT

CTAGAAGATCTCCTACAATATTCTCAGCTGCCATGGAAAATCGATGTTCT

TCTTTTATTCTCTCAAGATTTTCNGGCTGTATATTAAAACTTATATTAAG

AACTATGCTAACCACCTCATCNGGAACCGTTGTNNNNGGCGTGGGTTTTC

TTGGCAATCGACTCTCATGAAACTACNAGCTAAATATTCA

>TC32

GATACCTATCCATCAGTTCCACCATGTCTCTTGCTGCGCTCTGCCACACA

ATTTGCCATATTCTTGTACTGTTTCGTGCTGTCGTTGTGCTGTACTACGG

AATCTGTACGTCACAATCGGATTAAATAAACTGACCCGGATTATCTACCT

ATCCTTTTCTTCTCTACAGATCGTAATGCGCAAAACCCCAGCCCCGATCA

ACGCAGACTCCCATTAACTGCGAACGAGAGTGTCGATGAGCTGTAAATGA

CGTTCGAAACGACATAAATGCAGACGCGCTATAAAACCTCAGCCTCAAAA

ACCGCACCTTGCAGGCAAAAGGCCCCCGAAGGCACACATAAAACCGTGAT

CCCAAAGCCCGCGTAATAGGGATTATGTTACACATACGCTTGCACACAGA

GGGCTCCAAAATGTCAAAGTTACACGCGTGTGGAGTGTCTCCGTGGCGCT

ATCTCTGTGACCAACTTCCCAGCGAAAAACCAAACCCCAGCAGGTGAATA

AAAAAGGGAAAACTGAGCCGCTTCAACAAAACAAGTGCTGCTATGGGTTC

GTTGGCTGCCCTTTTTCTCCGTATCCGAATGCACCTTTAGCTGCTGGACA

ATGGCTAATTTATTTCAACCTAAACGCTCTGCTCTGCTCGTAAATTTGTT

TAATAGCGTTAATTAAGGTGAAATAAAATATTAGTTAATTTCAGCTCTAG

CACCTCTTCGACCACCAATTCGTGTGCGATAAATTAGCGATTTTTATGTG

ATCTCCCACTCCCCCCAAAAACTGCCACAGATTNNCCTGATCTTTCTAGA

AGATCTCCTACAATATTCTCAGCTGCCATGGAAAATCGATGTTCTTCTTT

TATTCTCTCAAGANTTTCNNCTGTATATTAAAACTTATATTAAGAACTAT

GCTAACCACCTCATCAGGAACCGTTGTANTGGCGTGNNTTTCTTGGCAAT

CGACTCTCATGAAACTACGAGCTAAATATTCA

>T18

GATACCTATCCATCAGTTCCACCATGTCTCTTGCTGCGCTCTGCCACACT

ATTTGCCATATTCTTGTACTGTTTCGTGCTGTCGTTGTGCTGTACTACGG

AATCTGTACGTCACAATCGGATTAAATAAACTGACCCGGATTATCTACCT

ATCCTTTTCTTCTCTACAGATCGTAATGCGCAAAACCCCAGCCCCGATCA

ACGCAGACTCCCATTAACTGCGAACGAGAGTGTCGATGAGCTGTAAATGA

CGATCGAAACGACATAAAATGCAGACGCGCTATAAAACCTCAGCCTCAAA

AACCGCACCTTGCAGGCAAAAGGCCCCCGAAGGCACACATAAAACCGTGA

TCCCAAAGCCCGCGTAATAGGGATTATGTTACACATACGCTTGCACACAG

AGGGCTCCAAAATGTCAAAGTTACACGCGTGTGGAGTGTCTCCATGGCGC

TATCTCCGTGACCAACTTCCCAGCGAAAAGCCACCAACCCCCAGCAGGTG

AATTAAAAAAAGGGAAAACTGAGCCGCTTCTACAAAACAAGTGCTGCGAT

GGGTTCGTTGGCTGCCCTTTTTCTCCGTACCCGAATGCACCTTTTGCTGC

TGGACAATGGCTAATTTATTTCAACCTAAACGCTCTGCTCTGCTCGTAAA

TTTGTTTAATAGCGTTAATTAAGGTGAAATAAAATATTAGTTAATTTCAG

CTCTAGCACCTCTTCGACCACCAATTCGTGTGCGATAAATTAGCGATTTT

TATGTGATCTCCCACTCCCCAAAAAAACGCACTGCCACAGATTNNCCTGA

TCTTTCTAGAAGATCTCCTACAATATTCTCAGCTGCCATGGAAAATCGAT

GTTCTTCTTTTATTCTCTCAAGATTTTCAGGCTGTATATTAAAACTTATA

TTAANAACTATGCTAACCACCTCATCNNNACCGTTGTNGGTGGCGTGGGT

TTTCTTGGCATCGACTCTCATGAAAACTAC

>T7

GATACCTATCCATCAGTTCCACCATGTCTCTTGCTGCGCTCTGCCACACA

ATTTGCCATATTCTTGTACTGTTTCGTGCTGTCGTTGTGCTGTACTACGG

AATCTGTACGTCACAATCGGATTAAATAAACTGACCCGGATTATCTACCT

ATCCTTTTCTTCTCTACAGATCGTAATGCGCAAAACCCCAGCCCCGATCA

ACGCAGACTCCCATTAACGGCGAACGAGAGTGTCGATGAGCTGTAAATGA

CGATCGAAACGACATAAATGCAGACGCGCTATAAAACCTCAGCCTCAAAA

ACCGCACCTTGCAGGCAAAAGGCCCCCGAAGGCACACATAAAACCAGGGT

CTCCAAGCCCGCGTAATAGGGATTATGTTACACATACGCTTGAACACAGA

GGGCCCCAAAATGTCAAAGTTACACACAGTAACTGTGTGTCTCCATGGCG

CTATCTCCGTGACCAACTTCCCAGCGAAAAACCAAACCCCAGCAGGTGAA

TTGAAAAAAAGGGAAAACTGAGCTGCTTCAACAAAACAAGTGCTGCGATG

GGTTCGTTGGCTGCCCTTTTTCTCCGTACCCGAATGCACCTTTAGCTGCT

GGACAATGGCTAATTTATTTCAACCTAAACGCTCTGCTCTGCTCGTAAAT

TTGTTTAATAGCGTTAATTAAGGTGAAATAAAATATTAGTTAATTTCAGC

TCTAGCACCTCTCCGACCACCAATTCGTGTGCGATAAATTAGTGATTTTT

ATGTGATCTCCCACTCCCCAAAAAAACGCACTGCCACAGATTANGCCTGA

TCTTTCTAGAAGATCTCCTACAATATTCTCAGCTGCCATGGAAAATCGAT

GTTCTTCTTTTATTCTCTCAAGATTTTCNNNTGTATATTAAAACTTATAT

TAAGAACTATGCTAACCACCTCATCAGGAACCGTTGTANNGGCGTGNTTT

TCTTGGCAATCGACTCTCATGAAAACTACGAGCTAAATATTC

>T12

ATACCTATCCATCAGTTCCACCATGTCTCTTGCTGCGCTCTGCCACACTA

TTTGCCATATTCTTGTACTGTTTCGTGCTGTCGTTGTGCTGTACTACGGA

ATCTGTACGTCACAATCGGATTAAATAAACTGACCCGGATTATCTACCTA

TCCTTTTCTTCTCTACAGATCGTAATGCGCAAAACCCCAGCCCCGATCAA

CGCACACTCCCATTAACTGCGAACGAGAGTGTCGATGAGCTGTAAATGAC

GTTCGAAACGACATAAATGCAGACGCGCTATAAAACCTCTGCCTCAAAAA

CCGCACCTTACAGGCAAAAGGCCCCCGAAGGCACACATAAAACCAGGGTC

TCCAAGCCCGCGTAATAGGGATTATGTTACACATACGCTTGCACACAGAG

GGCTCCAAAATGTCAAAGTTACACGCGTGTGGAATGTCTCCATGGCGCTA

TCTCCGTGACCAACTTCCCAGCGAAAAACCAACCCCCAGCAGGTGAATTG

AAAAAAGGGAAAACTGAGCCGCTTCAACAAAACAAGTGCTGCGATGGGTT

CGTTGGCTGCCCTTTTTCTCCGTACCCGAATGCACCTTTAGCTGCTGGAC

AATGGCTAATTTATTTCAACCTAAACGCTCTGCTCTGCTCGTAAATTTGT

TTAATAGCGTTAATTCAGGTGAAATAAAATATTAGTTAATTTCAGCTCTA

GCACCTCTCCGACCACCAATTCGTGTGCGATAAATTAGCGATTTTTATGT

GATCTCCCACTCCCCCCAAAAACTGCCACAGATTAGGCCTGATCTTTCTA

GAAGATCTCCTACAATATTCTCAGCTGCCATGGAAAATCGATGTTCTTCT

TTTNNNNNCTCAAGATTTTCNNGCTGTATATTAAAACTTATATTAAGAAC

TATGCTAACCACCTCATCAGGAACCGTTGT

>T2

ATACCTATCCATCAGTTCCACCATGTCTCTTGCTGCGCTCTGCCACACAA

TTTGCCATATTCTTGTACTGTTTCGTGCTGTCATTGTGCTGTACTACGGA

ATCTGTACGTCACAATCGGATTAAATAAACTGACCCGGATTATCTACCTA

TCCTTTTCTTCTCTACAGATCGTAATGCGCAAAACCCCAGCCCCGATCAA

CGCACACTCCCATTAACTGCGAACGAGAGTGTCGATGAGCTGTAAATGAC

GTTCGAAACGACATAAATGCAGACGCGCTATAAAACCTCAGCCTCAAAAA

CCGCACCTTGCAGGCAAAAGGCCCCCGAAGGCACACATAAAACCGTGATC

CCAAAGCCCGCGTAATAGGGATTATGTTACACATACGCTTGCACACAGAG

GGCTCCAAAATGTCAAAGTTACACGCGTGTGTCTCCGTGGCGCTATCTCC

GTGACCAACTTCCCAGCGAAAAACCACCAACCCCCAGCAGGTGAATTAAA

AAAAGGGAAAACTGAGCCGCTTCAACAAAACAAGTGCTGCGATGGGTCGT

TGGCTGCCCTTTTTCTCCGTACCCAAATGCACCTTTTGCTGCTGGACAAT

GGCTAATTTATTTCAACCTAAACGCTCTGCTCTGCTCGTAAATTTGTTTA

ATAGCGTTAATTAAGGTGAAATAAAATATTAGTTAATTTCAGCTCTAGCA

CCTCTTCGACCACCAATTCGTGTGCGATAAATTAGCGATTTTTATGTGAT

CTCCCACTCTCCCCCGACAAAAAAAAACGCACTGCCACAGATTANGCCTG

ATCTTTCTAGAAGATCTCCTACAATATTCTCAGCTGCCATGGAAAATCGA

TGTTCTTCTTTTATTCTCTCAAGATTTTCAGGCTGTATATTAAAACTTAT

ATTAAGAACTATGCTAACCACCTCATC

>TC8

ATACCTATCCATCAGTTCCACCATGTCTCTTGCTGCGCTCTGCCACACTA

TTTGCCATATTCTTGTACTCTTTCGTGCTGTCGTTGTGCTGTACTACGGA

ATCTGTACGTCACAATCGGATTAAATAAACTGACCCGGATTATCTACCTA

TCCTTTTCTTCTCTACAGATCGTAATGCGCAAAACCCCAGCCCCGATCAA

CGCAGACTCCCATTAACTGCGAACGAGAGTGTCGATGAGCTGTAAATGAC

GATCGAAACGACATAAATGCAGACGCGCTATAAAACCTCAGCCTCGAAAA

CCGCACCTTGCAGGCAAAAGGCCCCCGAAGGCACACATAAAACCGTGGTC

CCCAAGCCCGCGTAATAGGAATTATGTTACCCATACGCTTGCACACAGAG

GGCCCCAAAATGTCAAAGTTACACGCGTGTGTCTCCGTGGCGCTATCTCT

GTGACCAACTTCCCAGCGAAAAGCCACCAACCCCCAGCAGGTGAATTGAA

AAAAAGGGAAAACTGAGCTGCTTCAACAAAACAAGTGCTGCGATGGGTTC

GTTGGCTGCCCTTTTTCTCCGTACCCAAATGCACCTTTAGCTGCCGGACA

ATGGCTAATTTATTTCAACCTAAACGCTCTGCTCTGCTCGTAAATTTGTT

TAATAGCGTTAATTAAGGTGAAATAAAATATTAGTTAATTTCAGCTCTAG

CACCTCTTCGACCACCAATTCGTGTGCGATAAATTAGCGATTTTTATGTG

ATCTCCCACTCCCCCCAAAAACTGCCACAGATTANGCCTGATCTTTCTAG

AAGATCTCCTACAATATTCTCAGCTGCCATGGAAAATCGATGTTCTTCTT

TTATTCTCTCAAGATTTTCNNGCTGTATATTAAAACTTATATTAAGAACT

ATGCTAACCACCTCATCNNAACCGTTGTNNTGGCGTGGGTTTTCTTGGCA

ATCGACTCTCATGAAAACTACNAGCTAAATATTCA

>T13

ATCCTATCCATCAGTTCCACCATGTCTCTTGCTGCGCTCTGCCACACAAT

TTGCCATATTCTTGTACTGTTTCGTGCTGTCGTTGTGCTGTACTACGGAA

TCTGTACGTCACAATCGGATTAAATAAACTGACCCGGATTATCTACCTAT

CCTTTTCTTCTCTACAGATCGTAATACGCAAAACCCAAGCCCCGATCAAC

GCAGACTCCCATTAACGGCGAACGAGAGTGTCGATGAGCTGTAAATGACG

ATTGAAACGACATAAATGCAGACGCGATATAAAACCTCAGCCTCAAAAAC

CGCACCTTGCAGGCAAAAGGCCCCTGAAGGCACACATAAAACCGTAGTCC

CAAAGCCCGCGTAATAGGGATTATGTTACACATACGCTTGCACACAGAGG

GCTCCAAAATGTCAAAGTTACACGCGTGTGTCTCCGTGGCGCTATCTCCG

TGACCAACTTCCCAGCGAAAAGCCACCAACCCCCAGCAGGTGAATTAAAA

AAAGGGAAAACTGAGCCGCTTCAACAAAACAAGTGCTGCGATGGTTCGTT

GGCTGCCCTTTTTCTCCGTACCCAAATGCACCTTTAGCTGCCGGACAATG

GCTAATTTATTTCAACCTAAACGCTCTGCTCTGCTCGTAAATTTGTTTAA

TAGCGTTAATTAAGGTGAAATAAAATATTAGTTAATTTCAGCTCTAGCAC

CTCTCCGACCACCAATTCGTGTGCGATAAATTAGCGATTTTTATGTGATC

TCCCACTATCCCCCAAAAACTGCCACAGATTANGCCTGATCTTTCTAGAA

GATCTCCTACAATATTCTCAGCTGCCATGGAAAATCGATGTTCTTCTTTT

ATTCTCTCAAGANTTTCAGGCTGTATATTAAAACTTATATTAAGAACTAT

GCTAACCACCTCATCNGNACCGTTGTAGGTGGCGNGGGTTTTCTTGGCAA

TCGACTCTCATGAAACTACGANCTAATATTCAATATGT

>T3J

TACAGATCGTAATGCGCAAAACCCCAGCCCCGATCANNGCAGACTCCCAT

AACGGCGAACGAGAGTGTNGATGANCTGTAAATGACGTTCGAAANGACAT

AAATGCAGACGCGCTATAAAACCTCAGCCTCCAAAAAACCGCACCTTGCA

GGCAAAAGGCCCCCGAAGGCACACATAAAACCGTGGTCCCAATGCCCGCG

TAATAGGGATTATGTTACCCATACGCTTGCACACAGAGGGCTCCAAAATG

TCAAAGTTACACGCGTGTGTCTCCGTGGCGCTATCTCCGTGACCAACTTC

CCAGCGAAAAACCAAACCCCAGCAGGTGAATTGAAAAAAAGGGAAAACTG

AGCTGCTTCAACAAAACAAGTGCTGCGATGGGTTCGTTGGCTGCCCTTTT

TCTCCGTACCCGAATGCACCTTTAGCTGCTGGACAATGGCTAATTTATTT

CAACCTAAACGCTCTGCTCTGCTCGTAAATTTGTTTAATAGCGTTAATTA

AGGTGAAATAAAATATTAGTTAATTTCAGCTCTAGCACCTCTTCGACCAC

CAATTCGTGTGCGATAAATTAGCGATTTTTATGTGATCTCCCACTCTCCC

CCAACAAAAAAAAAACGCACTGCCACAGATTAGGCCTGATCTTGCNGAAA

AACTCGAGCCC
