# Supplementary figures and images for: Genome-wide transcriptional analyses in Anopheles mosquitoes reveal an unexpected association between salivary gland gene expression and insecticide resistance
Source: BMC Genomics. 2018 Mar 27;19:225. doi: 10.1186/s12864-018-4605-1 (PMC5870100; doi:10.1186/s12864-018-4605-1)

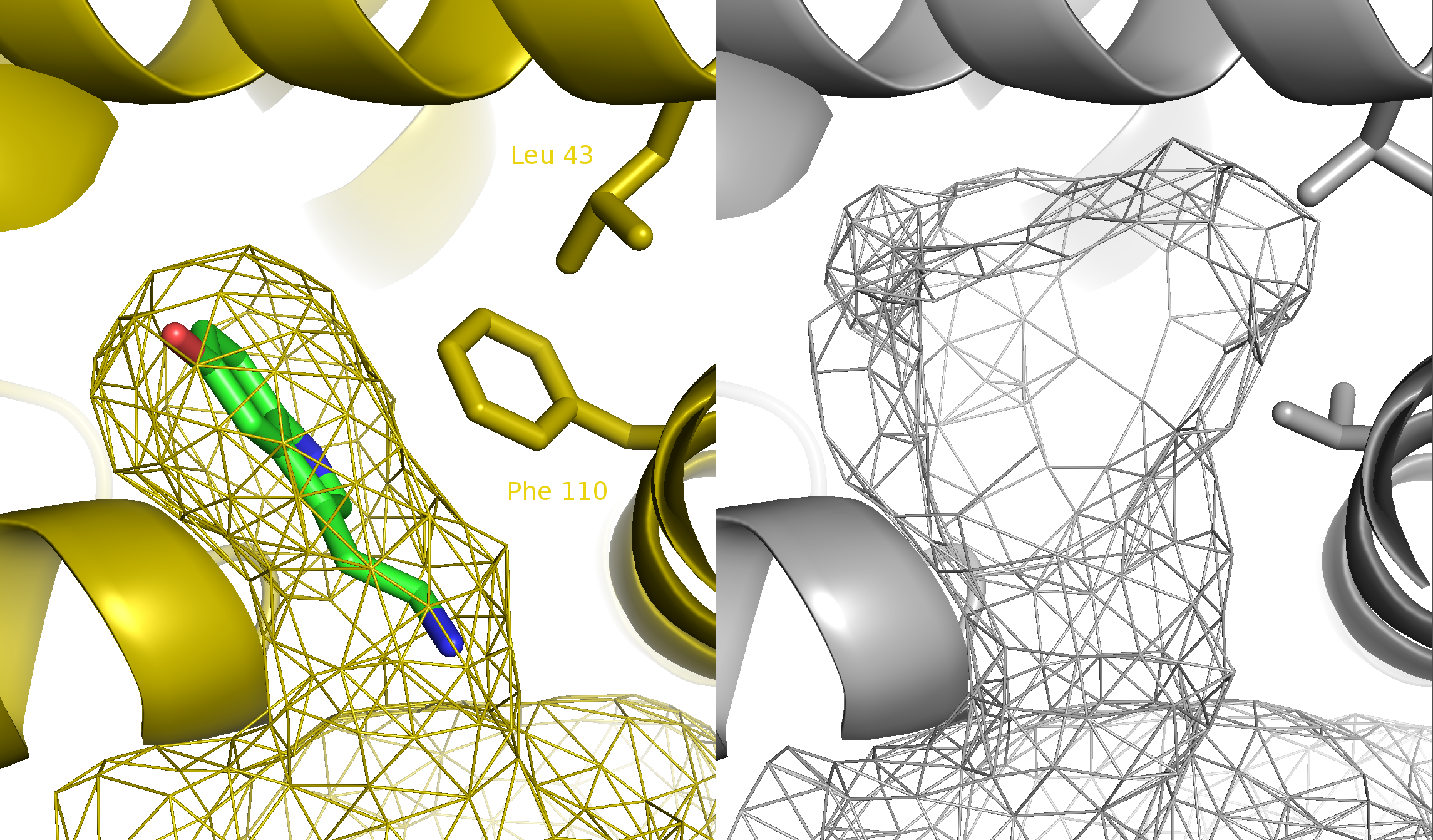

Supplement: Supplementary file 4 — Comparison of the cavity binding serotonin in the D7r4 crystal structure (PDB code 2qeh; [15]) and the larger cavity predicted for modelled D7r2, largely due to the replacement of Phe110 and Leu43 with Val residues. The figure was made with PyMOL (pymol.org). (PNG 1766 kb) [file 12864_2018_4605_MOESM4_ESM.png]
