# Supplementary material for: Genome-wide transcriptional analyses in Anopheles mosquitoes reveal an unexpected association between salivary gland gene expression and insecticide resistance
Source: BMC Genomics. 2018 Mar 27;19:225. doi: 10.1186/s12864-018-4605-1 (PMC5870100; doi:10.1186/s12864-018-4605-1)

	Kisumu Control	Nagongera exposed	Nagongera control	Kihihi exposed	Kihihi control
Replicate 1	0.1	1.1	2.1	3.1	4.1
Replicate 2	0.2	1.2	2.2	3.2	4.2
Replicate 3	0.3	1.3	2.3	3.3	4.3
Replicate 4	0.4	1.4	2.4	3.4	4.4

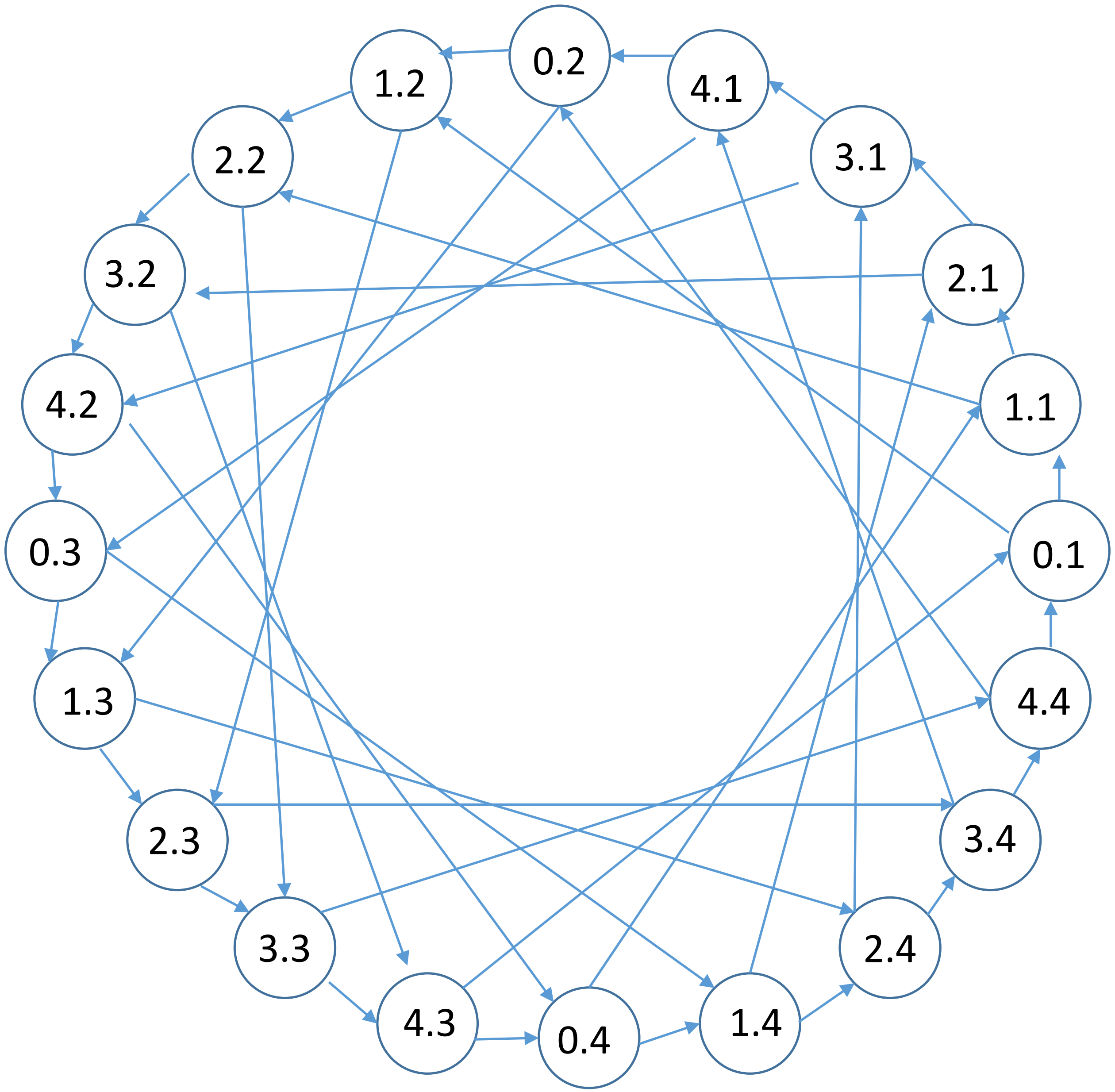

Supplement: Supplementary file 5 — Diagram of hybridizations performed in the microarray analysis. (PDF 117 kb) [file 12864_2018_4605_MOESM5_ESM.pdf]
