# Supplementary material for: Genome-wide transcriptional analyses in Anopheles mosquitoes reveal an unexpected association between salivary gland gene expression and insecticide resistance
Source: BMC Genomics. 2018 Mar 27;19:225. doi: 10.1186/s12864-018-4605-1 (PMC5870100; doi:10.1186/s12864-018-4605-1)
Supplement: Supplementary file 8 — Articles considered in the meta-analysis of Anopheles insecticide resistance microarrays. (DOCX 20 kb) [file 12864_2018_4605_MOESM8_ESM.docx]

Pubmed Search Results:

1: Antonio-Nkondjio C, Poupardin R, Tene BF, Kopya E, Costantini C, Awono-Ambene

P, Wondji CS. Investigation of mechanisms of bendiocarb resistance in Anopheles

gambiae populations from the city of Yaoundé, Cameroon. Malar J. 2016 Aug

22;15(1):424. doi: 10.1186/s12936-016-1483-3. PubMed PMID: 27549778; PubMed

Central PMCID: PMC4994282.

2: Samb B, Konate L, Irving H, Riveron JM, Dia I, Faye O, Wondji CS.

Investigating molecular basis of lambda-cyhalothrin resistance in an Anopheles

funestus population from Senegal. Parasit Vectors. 2016 Aug 12;9(1):449. doi:

10.1186/s13071-016-1735-7. PubMed PMID: 27519696; PubMed Central PMCID:

PMC4983014.

3: Ngufor C, N'Guessan R, Fagbohoun J, Subramaniam K, Odjo A, Fongnikin A,

Akogbeto M, Weetman D, Rowland M. Insecticide resistance profile of Anopheles

gambiae from a phase II field station in Cové, southern Benin: implications for

the evaluation of novel vector control products. Malar J. 2015 Nov 18;14:464.

doi: 10.1186/s12936-015-0981-z. PubMed PMID: 26581678; PubMed Central PMCID:

PMC4652434.

4: Platt N, Kwiatkowska RM, Irving H, Diabaté A, Dabire R, Wondji CS. Target-site

resistance mutations (kdr and RDL), but not metabolic resistance, negatively

impact male mating competiveness in the malaria vector Anopheles gambiae.

Heredity (Edinb). 2015 Sep;115(3):243-52. doi: 10.1038/hdy.2015.33. PubMed PMID:

25899013; PubMed Central PMCID: PMC4519523.

5: Toé KH, N'Falé S, Dabiré RK, Ranson H, Jones CM. The recent escalation in

strength of pyrethroid resistance in Anopheles coluzzi in West Africa is linked

to increased expression of multiple gene families. BMC Genomics. 2015 Mar

1;16:146. doi: 10.1186/s12864-015-1342-6. PubMed PMID: 25766412; PubMed Central

PMCID: PMC4352231.

6: Ingham VA, Jones CM, Pignatelli P, Balabanidou V, Vontas J, Wagstaff SC, Moore

JD, Ranson H. Dissecting the organ specificity of insecticide resistance

candidate genes in Anopheles gambiae: known and novel candidate genes. BMC

Genomics. 2014 Nov 25;15:1018. doi: 10.1186/1471-2164-15-1018. PubMed PMID:

25421852; PubMed Central PMCID: PMC4256904.

7: Nkya TE, Poupardin R, Laporte F, Akhouayri I, Mosha F, Magesa S, Kisinza W,

David JP. Impact of agriculture on the selection of insecticide resistance in the

malaria vector Anopheles gambiae: a multigenerational study in controlled

conditions. Parasit Vectors. 2014 Oct 16;7:480. doi: 10.1186/s13071-014-0480-z.

PubMed PMID: 25318645; PubMed Central PMCID: PMC4201709.

8: Wilding CS, Weetman D, Rippon EJ, Steen K, Mawejje HD, Barsukov I, Donnelly

MJ. Parallel evolution or purifying selection, not introgression, explains

similarity in the pyrethroid detoxification linked GSTE4 of Anopheles gambiae and

An. arabiensis. Mol Genet Genomics. 2015 Feb;290(1):201-15. doi:

10.1007/s00438-014-0910-9. PubMed PMID: 25213601; PubMed Central PMCID:

PMC4312195.

9: Matowo J, Jones CM, Kabula B, Ranson H, Steen K, Mosha F, Rowland M, Weetman

D. Genetic basis of pyrethroid resistance in a population of Anopheles

arabiensis, the primary malaria vector in Lower Moshi, north-eastern Tanzania.

Parasit Vectors. 2014 Jun 19;7:274. doi: 10.1186/1756-3305-7-274. PubMed PMID:

24946780; PubMed Central PMCID: PMC4082164.

10: Thomsen EK, Strode C, Hemmings K, Hughes AJ, Chanda E, Musapa M, Kamuliwo M,

Phiri FN, Muzia L, Chanda J, Kandyata A, Chirwa B, Poer K, Hemingway J, Wondji

CS, Ranson H, Coleman M. Underpinning sustainable vector control through informed

insecticide resistance management. PLoS One. 2014 Jun 16;9(6):e99822. doi:

10.1371/journal.pone.0099822. PubMed PMID: 24932861; PubMed Central PMCID:

PMC4059741.

11: Abdalla H, Wilding CS, Nardini L, Pignatelli P, Koekemoer LL, Ranson H,

Coetzee M. Insecticide resistance in Anopheles arabiensis in Sudan: temporal

trends and underlying mechanisms. Parasit Vectors. 2014 May 8;7:213. doi:

10.1186/1756-3305-7-213. PubMed PMID: 24886129; PubMed Central PMCID: PMC4026821.

12: Edi CV, Djogbénou L, Jenkins AM, Regna K, Muskavitch MA, Poupardin R, Jones

CM, Essandoh J, Kétoh GK, Paine MJ, Koudou BG, Donnelly MJ, Ranson H, Weetman D.

CYP6 P450 enzymes and ACE-1 duplication produce extreme and multiple insecticide

resistance in the malaria mosquito Anopheles gambiae. PLoS Genet. 2014 Mar

20;10(3):e1004236. doi: 10.1371/journal.pgen.1004236. PubMed PMID: 24651294;

PubMed Central PMCID: PMC3961184.

13: Nkya TE, Akhouayri I, Poupardin R, Batengana B, Mosha F, Magesa S, Kisinza W,

David JP. Insecticide resistance mechanisms associated with different

environments in the malaria vector Anopheles gambiae: a case study in Tanzania.

Malar J. 2014 Jan 25;13:28. doi: 10.1186/1475-2875-13-28. PubMed PMID: 24460952;

PubMed Central PMCID: PMC3913622.

14: Jones CM, Haji KA, Khatib BO, Bagi J, Mcha J, Devine GJ, Daley M, Kabula B,

Ali AS, Majambere S, Ranson H. The dynamics of pyrethroid resistance in Anopheles

arabiensis from Zanzibar and an assessment of the underlying genetic basis.

Parasit Vectors. 2013 Dec 6;6:343. doi: 10.1186/1756-3305-6-343. PubMed PMID:

24314005; PubMed Central PMCID: PMC3895773.

15: Nardini L, Christian RN, Coetzer N, Koekemoer LL. DDT and pyrethroid

resistance in Anopheles arabiensis from South Africa. Parasit Vectors. 2013 Aug

8;6(1):229. doi: 10.1186/1756-3305-6-229. PubMed PMID: 23924547; PubMed Central

PMCID: PMC3751093.

16: Hemingway J, Vontas J, Poupardin R, Raman J, Lines J, Schwabe C, Matias A,

Kleinschmidt I. Country-level operational implementation of the Global Plan for

Insecticide Resistance Management. Proc Natl Acad Sci U S A. 2013 Jun

4;110(23):9397-402. doi: 10.1073/pnas.1307656110. PubMed PMID: 23696658; PubMed

Central PMCID: PMC3677419.

17: Fossog Tene B, Poupardin R, Costantini C, Awono-Ambene P, Wondji CS, Ranson

H, Antonio-Nkondjio C. Resistance to DDT in an urban setting: common mechanisms

implicated in both M and S forms of Anopheles gambiae in the city of Yaoundé

Cameroon. PLoS One. 2013 Apr 23;8(4):e61408. doi: 10.1371/journal.pone.0061408.

PubMed PMID: 23626680; PubMed Central PMCID: PMC3634070.

18: Kwiatkowska RM, Platt N, Poupardin R, Irving H, Dabire RK, Mitchell S, Jones

CM, Diabaté A, Ranson H, Wondji CS. Dissecting the mechanisms responsible for the

multiple insecticide resistance phenotype in Anopheles gambiae s.s., M form, from

Vallée du Kou, Burkina Faso. Gene. 2013 Apr 25;519(1):98-106. doi:

10.1016/j.gene.2013.01.036. PubMed PMID: 23380570; PubMed Central PMCID:

PMC3611593.

19: Riveron JM, Irving H, Ndula M, Barnes KG, Ibrahim SS, Paine MJ, Wondji CS.

Directionally selected cytochrome P450 alleles are driving the spread of

pyrethroid resistance in the major malaria vector Anopheles funestus. Proc Natl

Acad Sci U S A. 2013 Jan 2;110(1):252-7. doi: 10.1073/pnas.1216705110. PubMed

PMID: 23248325; PubMed Central PMCID: PMC3538203.

20: Nardini L, Christian RN, Coetzer N, Ranson H, Coetzee M, Koekemoer LL.

Detoxification enzymes associated with insecticide resistance in laboratory

strains of Anopheles arabiensis of different geographic origin. Parasit Vectors.

2012 Jun 7;5:113. doi: 10.1186/1756-3305-5-113. PubMed PMID: 22676389; PubMed

Central PMCID: PMC3430573.

21: Mitchell SN, Stevenson BJ, Müller P, Wilding CS, Egyir-Yawson A, Field SG,

Hemingway J, Paine MJ, Ranson H, Donnelly MJ. Identification and validation of a

gene causing cross-resistance between insecticide classes in Anopheles gambiae

from Ghana. Proc Natl Acad Sci U S A. 2012 Apr 17;109(16):6147-52. doi:

10.1073/pnas.1203452109. PubMed PMID: 22460795; PubMed Central PMCID: PMC3341073.

22: Koekemoer LL, Spillings BL, Christian RN, Lo TC, Kaiser ML, Norton RA, Oliver

SV, Choi KS, Brooke BD, Hunt RH, Coetzee M. Multiple insecticide resistance in

Anopheles gambiae (Diptera: Culicidae) from Pointe Noire, Republic of the Congo.

Vector Borne Zoonotic Dis. 2011 Aug;11(8):1193-200. doi: 10.1089/vbz.2010.0192.

PubMed PMID: 21417925.

23: Gregory R, Darby AC, Irving H, Coulibaly MB, Hughes M, Koekemoer LL, Coetzee

M, Ranson H, Hemingway J, Hall N, Wondji CS. A de novo expression profiling of

Anopheles funestus, malaria vector in Africa, using 454 pyrosequencing. PLoS One.

2011 Feb 25;6(2):e17418. doi: 10.1371/journal.pone.0017418. PubMed PMID:

21364769; PubMed Central PMCID: PMC3045460.

24: Félix RC, Müller P, Ribeiro V, Ranson H, Silveira H. Plasmodium infection

alters Anopheles gambiae detoxification gene expression. BMC Genomics. 2010 May

19;11:312. doi: 10.1186/1471-2164-11-312. PubMed PMID: 20482856; PubMed Central

PMCID: PMC2885368.

25: Lawson D, Arensburger P, Atkinson P, Besansky NJ, Bruggner RV, Butler R,

Campbell KS, Christophides GK, Christley S, Dialynas E, Hammond M, Hill CA,

Konopinski N, Lobo NF, MacCallum RM, Madey G, Megy K, Meyer J, Redmond S,

Severson DW, Stinson EO, Topalis P, Birney E, Gelbart WM, Kafatos FC, Louis C,

Collins FH. VectorBase: a data resource for invertebrate vector genomics. Nucleic

Acids Res. 2009 Jan;37(Database issue):D583-7. doi: 10.1093/nar/gkn857. PubMed

PMID: 19028744; PubMed Central PMCID: PMC2686483.

26: Djouaka RF, Bakare AA, Coulibaly ON, Akogbeto MC, Ranson H, Hemingway J,

Strode C. Expression of the cytochrome P450s, CYP6P3 and CYP6M2 are significantly

elevated in multiple pyrethroid resistant populations of Anopheles gambiae s.s.

from Southern Benin and Nigeria. BMC Genomics. 2008 Nov 13;9:538. doi:

10.1186/1471-2164-9-538. PubMed PMID: 19014539; PubMed Central PMCID: PMC2588609.

27: Awolola TS, Oduola OA, Strode C, Koekemoer LL, Brooke B, Ranson H. Evidence

of multiple pyrethroid resistance mechanisms in the malaria vector Anopheles

gambiae sensu stricto from Nigeria. Trans R Soc Trop Med Hyg. 2009

Nov;103(11):1139-45. doi: 10.1016/j.trstmh.2008.08.021. PubMed PMID: 18829056.

28: Chiu TL, Wen Z, Rupasinghe SG, Schuler MA. Comparative molecular modeling of

Anopheles gambiae CYP6Z1, a mosquito P450 capable of metabolizing DDT. Proc Natl

Acad Sci U S A. 2008 Jul 1;105(26):8855-60. doi: 10.1073/pnas.0709249105. PubMed

PMID: 18577597; PubMed Central PMCID: PMC2449330.

29: Vontas J, David JP, Nikou D, Hemingway J, Christophides GK, Louis C, Ranson

H. Transcriptional analysis of insecticide resistance in Anopheles stephensi

using cross-species microarray hybridization. Insect Mol Biol. 2007

Jun;16(3):315-24. PubMed PMID: 17433071.

30: Müller P, Donnelly MJ, Ranson H. Transcription profiling of a recently

colonised pyrethroid resistant Anopheles gambiae strain from Ghana. BMC Genomics.

2007 Jan 29;8:36. PubMed PMID: 17261191; PubMed Central PMCID: PMC1797171.

31: Vontas J, Blass C, Koutsos AC, David JP, Kafatos FC, Louis C, Hemingway J,

Christophides GK, Ranson H. Gene expression in insecticide resistant and

susceptible Anopheles gambiae strains constitutively or after insecticide

exposure. Insect Mol Biol. 2005 Oct;14(5):509-21. PubMed PMID: 16164607.

32: David JP, Strode C, Vontas J, Nikou D, Vaughan A, Pignatelli PM, Louis C,

Hemingway J, Ranson H. The Anopheles gambiae detoxification chip: a highly

specific microarray to study metabolic-based insecticide resistance in malaria

vectors. Proc Natl Acad Sci U S A. 2005 Mar 15;102(11):4080-4. PubMed PMID:

15753317; PubMed Central PMCID: PMC554807.

Additional search results from Google Scholar:

Riveron JM, Ibrahim SS, Chanda E, Mzilahowa T, Cuamba N, Irving H, Barnes KG,

Ndula M, Wondji CS: The highly polymorphic CYP6M7 cytochrome P450 gene partners

with the directionally selected CYP6P9a and CYP6P9b genes to expand the

pyrethroid resistance front in the malaria vector Anopheles funestus in Africa.

BMC Genomics 2014, 15:817.

Ibrahim SS, Ndula M, Riveron JM, Irving H, Wondji CS: The P450 CYP6Z1 confers

carbamate/pyrethroid cross-resistance in a major African malaria vector beside a

novel carbamate-insensitive N485I acetylcholinesterase-1 mutation. Mol Ecol 2016,

25:3436-3452.

Riveron JM, Yunta C, Ibrahim SS, Djouaka R, Irving H, Menze BD, Ismail HM,

Hemingway J, Ranson H, Albert A, Wondji CS: A single mutation in the GSTe2 gene

allows tracking of metabolically based insecticide resistance in a major malaria

vector. Genome Biol 2014, 15:R27.
